# Single-cell analysis reveals cellular reprogramming in advanced colon cancer following FOLFOX-bevacizumab treatment

**DOI:** 10.3389/fonc.2023.1219642

**Published:** 2023-07-28

**Authors:** Meiling Yang, Ciqiu Yang, Dong Ma, Zijun Li, Wei Zhao, Dongyang Yang

**Affiliations:** ^1^ Guangdong Cardiovascular Institute, Guangdong Provincial People’s Hospital, Guangdong Academy of Medical Sciences, Guangzhou, China; ^2^ Medical Research Institute, Guangdong Provincial People’s Hospital (Guangdong Academy of Medical Sciences), Southern Medical University, Guangzhou, China; ^3^ Department of Breast Cancer, Guangdong Provincial People’s Hospital (Guangdong Academy of Medical Sciences), Southern Medical University, Guangzhou, China; ^4^ Medical Oncology, Guangdong Provincial People’s Hospital (Guangdong Academy of Medical Sciences), Southern Medical University, Guangzhou, China; ^5^ Guangdong Provincial Institute of Geriatrics, Guangdong Provincial People’s Hospital (Guangdong Academy of Medical Sciences), Southern Medical University, Guangzhou, China; ^6^ Key Laboratory of Stem Cells and Tissue Engineering (Sun Yat-Sen University), Ministry of Education, Guangzhou, China

**Keywords:** advanced colorectal cancer, single-cell transcriptomic analysis, FOLFOX, bevacizumab, VEGF

## Abstract

**Introduction:**

The combination of FOLFOX and bevacizumab (FOLFOX-Bev) is a promising treatment for advanced colorectal cancer (CRC). However, the response of the tumor microenvironment to FOLFOX-Bev is still largely unexplored.

**Methods:**

We conducted single-cell transcriptomic analysis of CRC samples derived from a patient before and after treatment to gain insights into the cellular changes associated with FOLFOX-Bev treatment.

**Results:**

We found that cancer cells with high proliferative, metastatic, and pro-angiogenic properties respond better to FOLFOX-Bev treatment. Moreover, FOLFOX-Bev enhances CD8^+^ T cell cytotoxicity, thereby boosting the anti-tumor immune response. Conversely, FOLFOX-Bev impairs the functionality of tumor-associated macrophages, plasma cells, and cancer-associated fibroblasts, leading to a decrease in VEGFB-mediated angiogenesis. Furthermore, FOLFOX-Bev treatment reset intercellular communication, which could potentially affect the function of non-cancer cells.

**Discussion:**

Our findings provide valuable insights into the molecular mechanisms underlying the response of advanced CRC to FOLFOX-Bev treatment and highlight potential targets for improving the efficacy of this treatment strategy.

## Introduction

Colorectal cancer (CRC) is one of the leading causes of cancer-related deaths worldwide (1), with metastasis being the primary cause of mortality among CRC patients (2). Despite advancements in treatment, the prognosis for advanced CRC remains poor, with a 5-year survival rate of only 12% (3). FOLFOX, which is a combination of three chemotherapeutic agents (oxaliplatin, leucovorin, and fluorouracil), has emerged as a standard therapeutic option for patients with metastatic CRC. Bevacizumab, a monoclonal antibody targeting vascular endothelial growth factor (VEGF), was approved by the FDA as a first-line therapy for metastatic CRC in February 2004 (4). Despite the clinical benefits seen with bevacizumab, not all patients with metastatic CRC respond equally to the treatment, and the underlying mechanism of this variability remains poorly understood.

Recently, it has become increasingly evident that CRC is a highly heterogeneous disease, with the tumor microenvironment (TME) playing a critical role in tumor progression and treatment response. To improve the efficacy of chemotherapy combined with bevacizumab for the treatment of metastatic CRC, it is essential to comprehend the intricate cellular types and their functional characteristics in the TME. Single-cell transcriptomic analyses greatly contribute to our understandings in CRC, including intra-tumor cell heterogeneity (5, 6), cancer cell development driven by oncogenic mitogen-activated protein kinase (7), immunosuppressive landscape fostered by oncogenic mutations (8), immune phenotypic linkage between CRC and liver metastasis (9), and alterations of the TME in CRC with chemotherapy (10) or CSF1R blockade (11). However, the response of the TME to chemotherapy combined with bevacizumab is still largely unexplored. Therefore, there is an urgent need to investigate the cellular and molecular mechanisms underlying the response of the TME to FOLFOX-Bev, which could optimize the treatment strategy for metastatic CRC.

The aim of this study was to investigate the impact of FOLFOX-Bev treatment on the TME in advanced CRC using single-cell RNA sequencing (scRNA-seq). Our results revealed important insights into the cellular and molecular changes occurring within the TME following FOLFOX-Bev treatment. Specifically, we observed that cancer cells with high proliferative, metastatic, and pro-angiogenic potential exhibited increased sensitivity to FOLFOX-Bev treatment. Furthermore, we found that FOLFOX-Bev treatment enhanced the cytotoxicity of CD8^+^ T cells while impairing the functions of tumor-associated macrophages (TAMs), plasma cells, and cancer-associated fibroblasts (CAFs). Additionally, we identified significant inhibition of pathways affecting intercellular communication following FOLFOX-Bev treatment. These findings contribute to our understanding of the TME’s response to FOLFOX-Bev treatment in metastatic CRC and have significant implications for the development of personalized treatment strategies.

## Materials and methods

### Tumor samples

This study was conducted with the approval of the institutional review boards of Guangdong Provincial People’s Hospital, and informed consent was obtained from the patient with advanced CRC, whose tumor biopsy samples were collected before and after FOLFOX-Bev therapy for scRNA-seq.

### Preparation of single-cell suspensions and scRNA-seq

The preparation of single-cell suspensions and scRNA-seq was conducted according to previously published methods (12, 13). In brief, fresh tumor biopsy tissue was dissociated, erythrocytes were lysed, and cell viability was tested. The resulting single-cell suspension was then subjected to scRNA-seq using the 10x Genomics Single Cell protocol.

### scRNA-seq data preprocessing

The preliminary sequencing results were processed into fastq files using Cell Ranger (v3.0.2). The fastq files were aligned to the human reference genome hg19, and filtering and counting of barcodes and UMI were performed. The Seurat package (version 4.0.4; http://satijalab.org/seurat/) was used to analyze the resulting gene expression matrix. Cells with low quality (gene numbers < 200 or > 6,000, and with a percentage of mitochondrial genes > 50%) were filtered out, and genes detected in less than 10 cells were excluded. The data from the two samples underwent normalization using the default “LogNormalize” method. To address batch effects, we utilized the FindIntegrationAnchors function, employing canonical correlation analysis to identify a set of anchors. These anchors were subsequently used with the IntegrateData function (14) to integrate the objects, thereby correcting for batch effects. Ultimately, a total of 7,343 cells were included for further analysis.

### Cell clustering, visualization and annotation

We obtained gene expression data from 7,343 cells and performed principal component analysis to identify principal components. The clustering analysis was conducted using the Louvain algorithm, which was implemented through the “FindClusters” function of the Seurat package. The identified clusters and sub-clusters were visualized using uniform manifold approximation and projection (UMAP) or t-distributed stochastic neighbor embedding (t-SNE) analysis. We annotated cell clusters based on canonical cellular markers from the literature (10, 15, 16).

### Identification of cancer cells

We identified cancer cells using epithelial cell marker genes (EPCAM, KRT19, KRT18) and inferred copy number variations (CNVs) profiles as previously described in our studies (12, 13). We performed InferCNV within each sample using immune cells, including T cells, B cells, and myeloid cells, as the reference group and cancer cells as the observation group.

### Pathway analysis

To investigate the biological state differences of cancer cells between the sensitive and insensitive subgroups, we performed gene set enrichment analysis (GSEA) based on Hallmark gene sets using the fgsea package. We identified differentially expressed genes upregulated in cancer-associated fibroblast (CAF) metabolism cells using the “FindMarkers” function and screened them based on the condition of p_value < 0.05 and avg_log2FC > 0.5. We then performed Gene Ontology (GO) analysis using the R toolkit ClusterProfiler (17).

### Cell cycle analysis

We performed cell cycle analysis of cancer cells using the “CellCycleScoring” function, which scored the probability of S phase and G2M phase of each cell based on marker genes directly related to S phase and G2M phase provided by the Seurat package. The corresponding score indicated the degree of high expression of the S phase (or G2M phase) gene set in this cell. When both S phase score and G2M score were less than 0, the cells were in the G1 phase.

### Definition of cell signature

To examine the functional differences between cell populations before and after FOLFOX-Bev treatment, we calculated scores for functional gene sets using the “AddModuleScore” function. The functional gene sets included glycolysis (GO:0045821), fatty acid metabolism (KEGG:hsa00071), oxidative phosphorylation (KEGG:hsa00190), CD8^+^ T cell activation (GO:0036037), cytotoxicity (GO:0001913), M1/M2 polarization, pro-/anti-inflammatory, and angiogenesis gene sets, which were derived from previous published papers (15, 18).

### Trajectory analysis

We performed pseudotime trajectory analysis using the Monocle package (v2.20.0) (19–21). Raw counts from the Seurat RNA assays were used as inputs to create an object for pseudotime trajectory analysis. Data dimensionality was reduced using the “reduceDimension” function with the parameter reduction_method = “DDRTree”. The pseudotime trajectory was visualized using the “plot_cell_trajectory” function. Genes of interest were visualized using the “plot_pseudotime_heatmap” function and clustered into subgroups based on their gene expression patterns.

### Cell-cell communication analysis

Cell-cell communication analysis was performed using the CellChat package (version 1.1.2; https://github.com/sqjin/CellChat/) (22) to identify significant ligand-receptor pairs between different cell types.

### Statistics

Statistical analyses were conducted using the “stat_compare_means” function of the ggpubr package with two-tailed Student’s t-test and Wilcoxon Rank Sum Test.

## Results

### scRNA-seq profiling of TME in advanced colon cancer before and after FOLFOX-Bev treatment

**Figure 1:**
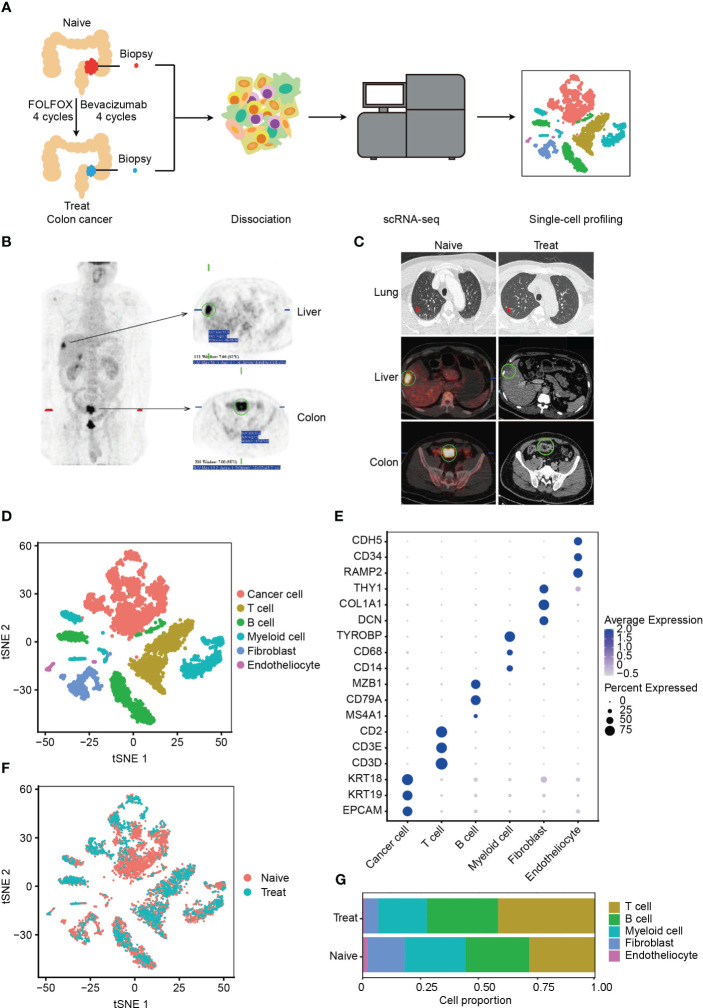
Single-cell RNA sequencing (scRNA-seq) profiling of the tumor microenvironment (TME) in advanced colon cancer before and after FOLFOX-Bev treatment. **(A)** Schematic diagram of the experimental design. Single-cell suspensions were obtained from advanced colon cancer before (Naïve) and after FOLFOX-Bev treatment (Treat), followed by scRNA-seq on the 10× Genomics platform. A total of 7,343 high-quality single cells were recovered. **(B)** Representative positron emission tomography (PET) pictures showing the distribution of tumors throughout the body for the advanced colon cancer patient before therapy. **(C)** Representative PET and computed tomography (CT) pictures for the advanced colon cancer patient before (Naïve) and after 4 cycles of FOLFOX-Bev treatment (Treat). **(D)** t-distributed stochastic neighbor embedding (t-SNE) plot displaying the indicated cell types in advanced colon cancer. **(E)** Dot plot illustrating the expression levels of marker genes in the indicated cell types. **(F)** t-SNE plot exhibiting the distribution of each cell type in advanced colon cancer before and after FOLFOX-Bev treatment. **(G)** Bar plots showing the cell proportions of each cell type in tumors before and after FOLFOX-Bev treatment.

To investigate the effects of FOLFOX-Bev treatment on the tumor microenvironment (TME) of advanced colon cancer, we collected biopsy samples from a patient with advanced colon cancer and multiple metastases (**Figure 1A**). Before treatment, positron emission tomography (PET) confirmed the diagnosis of sigmoid colon cancer with adjacent bladder wall involvement and multiple metastases to perienteric lymph nodes and liver, as well as multiple small nodules in both lungs (**Figure 1B**), indicating high metastatic potential. The patient received four cycles of FOLFOX chemotherapy and four cycles of bevacizumab, resulting in a partial response (**Figure 1C**). We obtained biopsy samples from the primary tumor before treatment (Naive) and after treatment (Treat) for further scRNA-seq analysis.

A total of 7,343 cells with 19844 genes passing the quality control stage were obtained for further analysis, and six distinct clusters were identified, corresponding to cancer cells (EPCAM, KRT19, KRT18), T cells (CD3D, CD3E, CD2), B cells (MS4A1, CD79A, MAB1), myeloid cells (CD14, CD68, TYROBP, LYZ), fibroblasts (DCN, COL1A1, THY1), and endothelial cells (RAMP2, CD34, CDH5) (**Figures 1D, E**, **S1A**). We confirmed that the cancer cells were copy number-unstable, with immune cells (including T cells, B cells, and myeloid cells) as control cells, using the inferCNV algorithm (**Figure S1B**).

We observed that all cell types were present in both Naive and Treat samples (**Figure 1F**), although the proportion of cell clusters varied (**Figure 1G**). Notably, the abundance of fibroblasts, and endothelial cells was reduced in the Treat sample, while the infiltration levels of T cells and B cells were relatively increased, suggesting that FOLFOX-Bev treatment may alter the TME of advanced colon cancers.

### Cancer cells with strong proliferation, metastasis and pro-angiogenesis are more sensitive to FOLFOX-Bev

**Figure 2:**
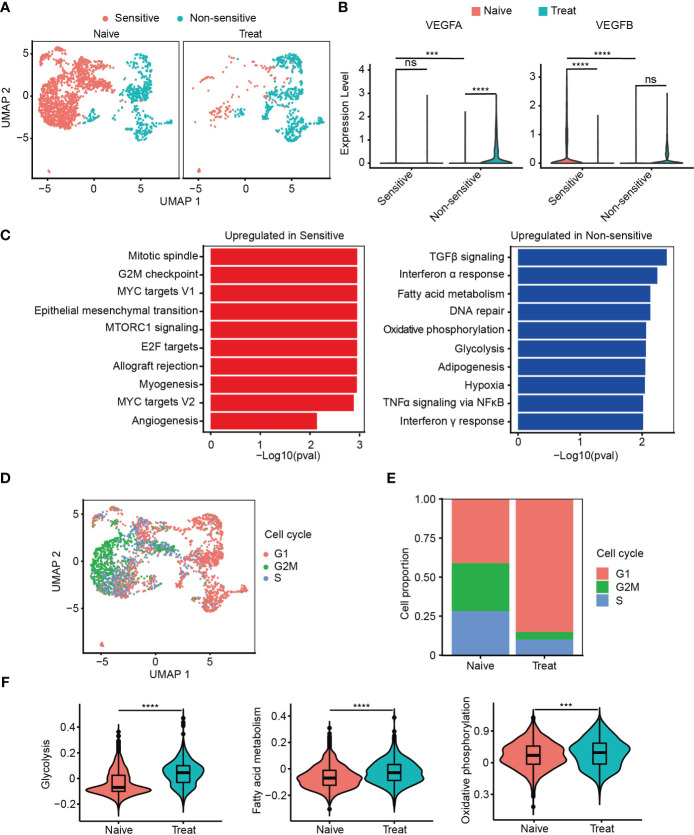
Cancer cells with strong proliferation, metastasis and pro-angiogenesis are more sensitive to FOLFOX-Bev. **(A)** Uniform manifold approximation and projection (UMAP) plot showing the distribution of cancer cell subpopulations in advanced colon cancer before (left) and after FOLFOX-Bev treatment (right). **(B)** Violin plot showing the expression levels of VEGFs in cancer cell subpopulations before (red) and after FOLFOX-Bev treatment (green). **(C)** Gene Set Enrichment Analysis (GSEA) of upregulated genes in sensitive or non-sensitive malignant cells, based on the HALLMARK gene set. **(D)** UMAP plot showing the distribution of cancer cells in different cell-cycle phases. **(E)** Bar plots depicting the cell proportion of cancer cells in different cell-cycle phases before and after FOLFOX-Bev treatment. **(F)** Violin plot showing the metabolic scores, including glycolysis, fatty acid metabolism, and oxidative phosphorylation, of cancer cells from the Naive (red) and Treat (green) samples. The p values were calculated using Student’s t-test. ***p ≤ 0.001; ****p ≤ 0.0001; ns, not significant (p ≥ 0.05).

Next, we classified cancer cells into two subgroups based on their sensitivity to FOLFOX-Bev treatment: a sensitive subgroup (Sensitive) and an insensitive subgroup (Non-sensitive) (**Figure 2A**). We then examined the expression of the pro-angiogenic factor VEGF (**Figure 2B**) and found that in the Naive sample, cancer cells mainly secreted VEGFB, which was predominantly expressed by the sensitive subset. However, its expression decreased in the Treat sample, while the insensitive subgroup upregulated the expression of VEGFA, indicating that FOLFOX-Bev treatment could influence the expression of VEGF.

Furthermore, we observed that the upregulated genes of the sensitive subgroup compared with the insensitive subgroup were significantly enriched in pathways related to proliferation (mitotic spindle, G2M checkpoint, MYC targets V1, E2F targets, MYC targets V2), metastasis (epithelial-mesenchymal transition), and angiogenesis (**Figure 2C**). This suggests that cancer cells with strong proliferation, metastasis, and pro-angiogenesis are more sensitive to FOLFOX-Bev treatment.

We then conducted cell cycle analysis and found that most of the cancer cells from the non-sensitive subgroup and the Treat sample were in the G1 phase, with a minor population in the G2M and S phases, compared to those from the sensitive subgroup and the Naive sample (**Figures 2D, E**). This suggests that FOLFOX-Bev treatment could induce G1 cell cycle arrest in cancer cells.

In addition, we observed that the upregulated genes of the non-sensitive subgroup were significantly enriched in energy metabolism pathways, including glycolysis, fatty acid metabolism, and oxidative phosphorylation (**Figure 2C**). We scored the energy metabolism signatures of cancer cells (**Figure 2F**), and found that glycolysis, fatty acid metabolism, and oxidative phosphorylation were significantly enhanced in the Treat sample, indicating that FOLFOX-Bev treatment could influence the energy metabolism of cancer cells. Overall, our results suggest that FOLFOX-Bev treatment could affect the expression of VEGF, cell cycle progression, and energy metabolism of cancer cells, and that cancer cells with high proliferation, metastasis, and pro-angiogenesis are more sensitive to this treatment.

### Impaired function of tumor-associated macrophages (TAMs) after FOLFOX-Bev treatment

**Figure 3:**
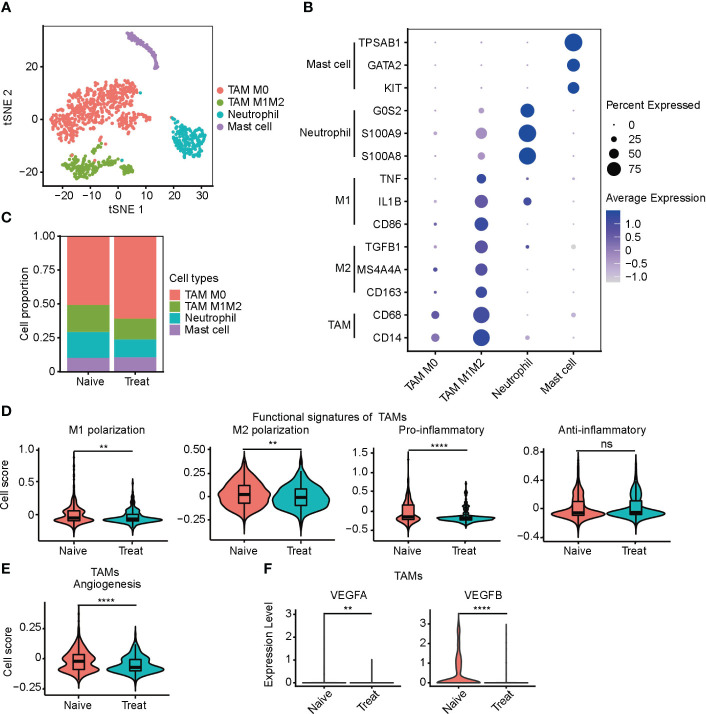
Impaired function of tumor-associated macrophages (TAMs) after FOLFOX-Bev treatment. **(A)** t-SNE plot depicting the subtypes of myeloid-derived cells in advanced colon cancer. **(B)** Dot plot showing the expression levels of marker genes in the myeloid cell subtypes. **(C)** Bar plots depicting the cell proportion of each myeloid cell subtype before and after FOLFOX-Bev treatment. **(D)** Violin plot showing the M1/M2 polarization, pro-inflammatory and anti-inflammatory scores of TAMs. The p values were calculated using a Wilcoxon Rank Sum Test. **(E)** Violin plot showing the angiogenesis score of TAMs. The p values were calculated using a Wilcoxon Rank Sum Test. **(F)** Violin plot showing the expression levels of VEGFs in TAMs before and after FOLFOX-Bev treatment. The statistical significance levels are denoted as follows: **p ≤ 0.01; ****p ≤ 0.0001; ns, not significant (p ≥ 0.05).

We conducted an unsupervised clustering analysis of myeloid cells and identified four distinct clusters, including two tumor-associated macrophages (TAMs) clusters, a neutrophil cluster, and a mast cell cluster (**Figure 3A**). TAM M0 cluster only expressed macrophage marker genes (CD14 and CD68) without expressing any polarization genes. In contrast, TAM M1M2 cluster expressed both M1 polarization genes (CD86, IL1B and TNF) and M2 polarization genes (CD163, MS4A4A and TGFB1). Neutrophils highly expressed neutrophil-specific genes (S100A8, S100A9, and G0S2), while mast cells highly expressed mast cell-specific genes (KIT, GATA2, and TPSAB1) (**Figure 3B**).

We observed that the infiltration level of TAMs, particularly the TAM M0 cluster, was significantly increased in the Treat samples (**Figure 3C**). To further examine the effect of FOLFOX-Bev treatment on the functional characteristics of TAMs, we calculated the M1/M2 polarization and pro-/anti-inflammatory scores of TAMs. Our analysis showed that the M1/M2 polarization and pro-inflammatory scores of TAMs decreased markedly after FOLFOX-Bev treatment (**Figure 3D**). Moreover, the angiogenic activity of TAMs was also suppressed by FOLFOX-Bev treatment, mainly indicated by a reduction in the expression of VEGFB (**Figures 3E, F**). Thus, TAMs appeared to be in a relatively quiescent state after FOLFOX-Bev treatment, with diminished functional activity.

### Enhanced cytotoxicity of CD8^+^ T cells after FOLFOX-Bev treatment

**Figure 4:**
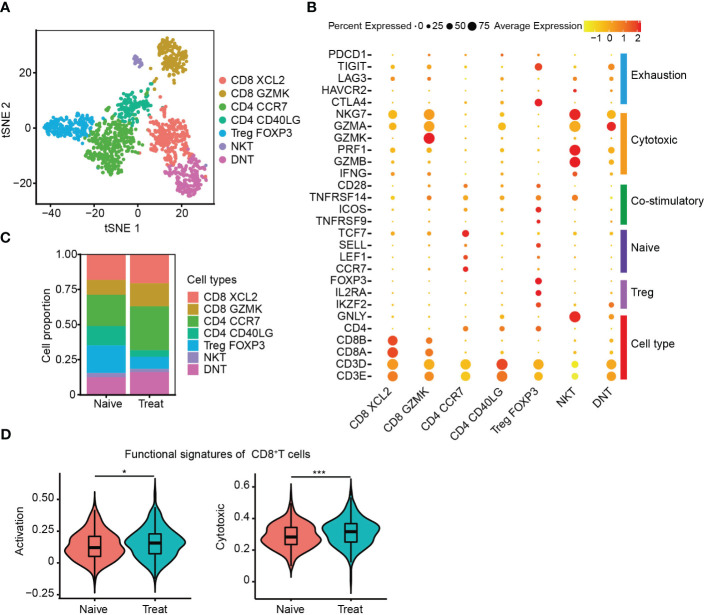
Enhanced cytotoxicity of CD8^+^ T cells after FOLFOX-Bev treatment. **(A)** t-SNE plot depicting the subtypes of T cells in advanced colon cancer. **(B)** Dot plot showing the gene sets of T-cell cytotoxicity, exhaustion, costimulation, naive T cells, and Treg cells. **(C)** Bar plots depicting the cell proportion of each T cell subtype before and after FOLFOX-Bev treatment. **(D)** Violin plot showing the activation and cytotoxic scores of CD8^+^ T cells. The p-values were calculated using a Student’s t-test. *p < 0.05; ***p ≤ 0.001.

The T cells were re-clustered into seven distinct groups (**Figure 4A**) based on their gene expression profiles, which included two CD8^+^ T cell clusters (CD8 XCL2 and CD8 GZMK) expressing cell-type genes CD3D, CD8A and CD8B, three CD4^+^ T cell clusters (CD4 CCR7, CD4 CD40LG and Treg FOXP3) expressing cell-type genes CD3D and CD4, one NKT subtype expressing cell-type genes CD3D and GNLY, and a double-negative T cell cluster called DNT, which was CD3^+^IKZF2^+^CD4^-^CD8^-^. CD4 CCR7 cells were a naive cluster with high expression of CCR7, LEF1 and TCF7, whereas Treg FOXP3 cells were an immunosuppressive cluster with high expression of FOXP3, IL2RA and IKZF2. The remaining clusters, including DNT, were cytotoxic, expressing cytotoxic genes such as NKG7, GZMA, GZMK, GZMB and PRF1, and were not cell cycle related (**Figures 4B**, **S2A-D**).

We then evaluated the distribution of T cell subpopulations and observed increased infiltration of CD8^+^ T cells and decreased infiltration of immunosuppressive Treg FOXP3 cells in the Treat sample (**Figure 4C**). CD8^+^ T cells are crucial for anti-tumor immunity, and therefore, we further examined the effect of FOLFOX-Bev treatment on their functional signatures. We extracted CD8^+^ T cells for further analysis and found that the activation and cytotoxic scores of CD8^+^ T cells were significantly elevated in the Treat sample compared to the Naïve sample (**Figure 4D**). These findings suggest that FOLFOX-Bev treatment might enhance anti-tumor immunity mediated by CD8^+^ T cells.

### Decreased angiogenic function of plasma cells after FOLFOX-Bev treatment

**Figure 5:**
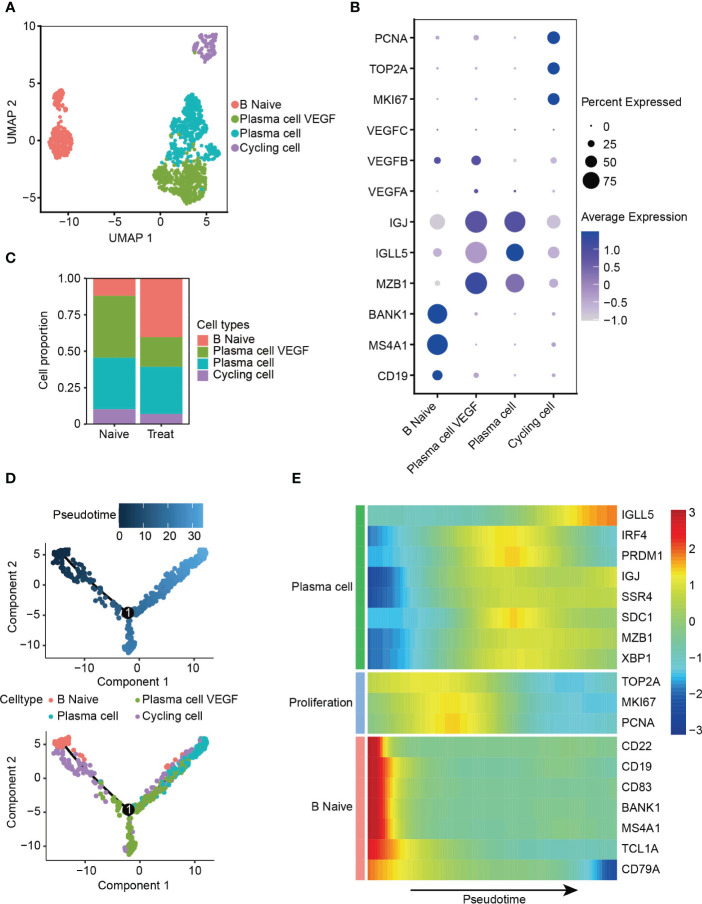
Decreased angiogenic function of plasma cells after FOLFOX-Bev treatment. **(A)** UMAP plot depicting the subtypes of B cells in advanced colon cancer. **(B)** Dot plot showing the expression levels of marker genes in the B cell subtypes. **(C)** Bar plots depicting the cell proportion of each B cell subtype before and after FOLFOX-Bev treatment. **(D)** Pseudotime-ordered analysis of B cells. B cell subtypes are labeled with colors. Each dot indicates a single cell. **(E)** Heatmap displaying the dynamic changes in gene expression along the pseudotime. The selected genes are associated with naïve B cells, proliferation, and plasma cells. The vertical color bar on the right represents the normalized gene expression levels. The pseudotime is displayed along the horizontal axis.

The B cells were re-clustered into four distinct subsets (**Figure 5A**), with B Naive cells exhibiting high expression of CD19, MS4A1 and BANK1. Two clusters of plasma cells were identified based on their high expression levels of MZB1, IGLL5 and IGJ, one of these clusters exhibited expression of VEGFA and VEGFB, thus called plasma cell VEGF. Cycling cells were identified as a proliferative cluster with high expression of MKI67, TOP2A and PCNA (**Figures 5B**, **S3A, B**). Interestingly, we observed increased infiltration of B Naive cells and reduced infiltration of plasma cells that release VEGF factors in the Treat sample, indicating a potential reduction in the angiogenic function of plasma cells mediated by VEGF factors following FOLFOX-Bev treatment (**Figure 5C**).

To further understand the dynamic cell transitions of B cell subsets, we used monocle for unsupervised analysis to construct the trajectory. We observed that B Naive cells and the proliferative cluster (Cycling cells) were located at the beginning of the trajectory, whereas the two plasma cell clusters were more concentrated at the end of the trajectory (**Figure 5D**). Moreover, three sets of differentially expressed genes were identified along the trajectory. The first set, consisting of naive B cell markers (CD22, CD19, MS4A1 and BANK1), decreased along the trajectory, while the second set, consisting of proliferative genes (MKI67, TOP2A, and PCNA), were highly expressed in the middle of the trajectory. The last set, consisting of plasma cell markers (IGLL5, IGJ and SDC1), increased towards the end of the trajectory (**Figure 5E**). These analyses reveal the dynamic cell transitions of B cells, from naive to terminally differentiated plasma cells, providing insight into the potential role of B cells in the immune response to cancer and the effects of FOLFOX-Bev treatment on this response.

### Impaired secretory function of cancer-associated fibroblasts (CAFs) after FOLFOX-Bev treatment

**Figure 6:**
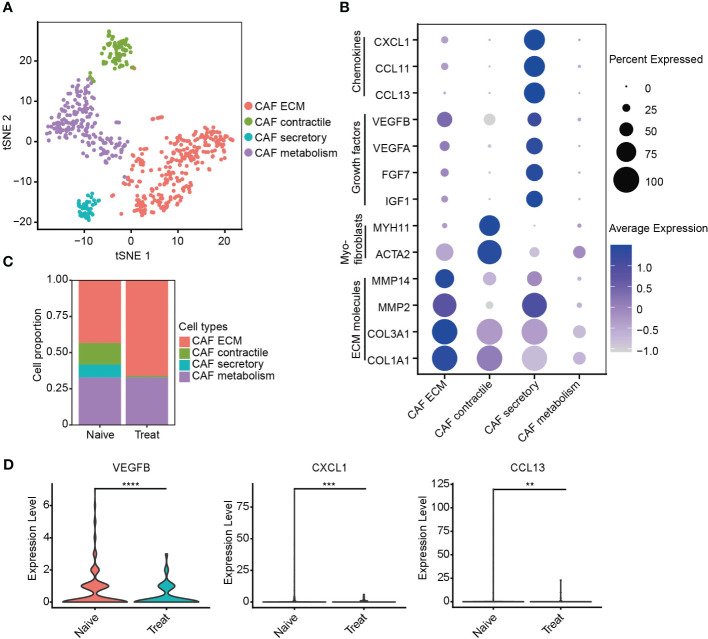
Impaired secretory function of cancer-associated fibroblasts (CAFs) after FOLFOX-Bev treatment. **(A)** t-SNE plot depicting the subtypes of CAFs in advanced colon cancer. **(B)** Dot plot showing the expression levels of selected gene sets in CAF subtypes, including ECM molecules, myofibroblasts, growth factors and chemokines. **(C)** Bar plots depicting the cell proportion of each CAF subtype before and after FOLFOX-Bev treatment. **(D)** Violin plot showing the expression levels of VEGFB, CXCL1, and CCL13 in CAF before and after FOLFOX-Bev treatment. The p values were calculated using a Student’s t test. **p ≤ 0.01; ***p ≤ 0.001; ****p ≤ 0.0001.

Next, we re-clustered cancer-associated fibroblasts (CAFs) into four distinct subtypes based on their gene expression profiles (**Figure 6A**). CAF ECM cells exhibited high expression of extracellular matrix (ECM) molecules such as COL1A1, COL3A1, MMP2, and MMP14. CAF contractile cells displayed high expression of myofibroblast markers ACTA2 and MYH11. CAF secretory cells demonstrated high expression of growth factors IGF1, FGF7, VEGFA, and VEGFB, as well as chemokines CCL13, CCL11, and CXCL1 (**Figures 6B**, **S4A**). CAF metabolism cells were significantly enriched in pathways related to RNA metabolism (**Figure S4B**).

Unexpectedly, our analysis revealed a significant decrease in the number of CAF secretory cells in the Treat sample (**Figure 6C**), indicating that FOLFOX-Bev treatment might have inhibited the secretory function of CAFs, including the reduction of VEGFB, CXCL1 and CCL13 secretion (**Figure 6D**), which could potentially impair the angiogenic function of CAFs.

### Altered intercellular communication in advanced colon cancer after FOLFOX-Bev treatment

**Figure 7:**
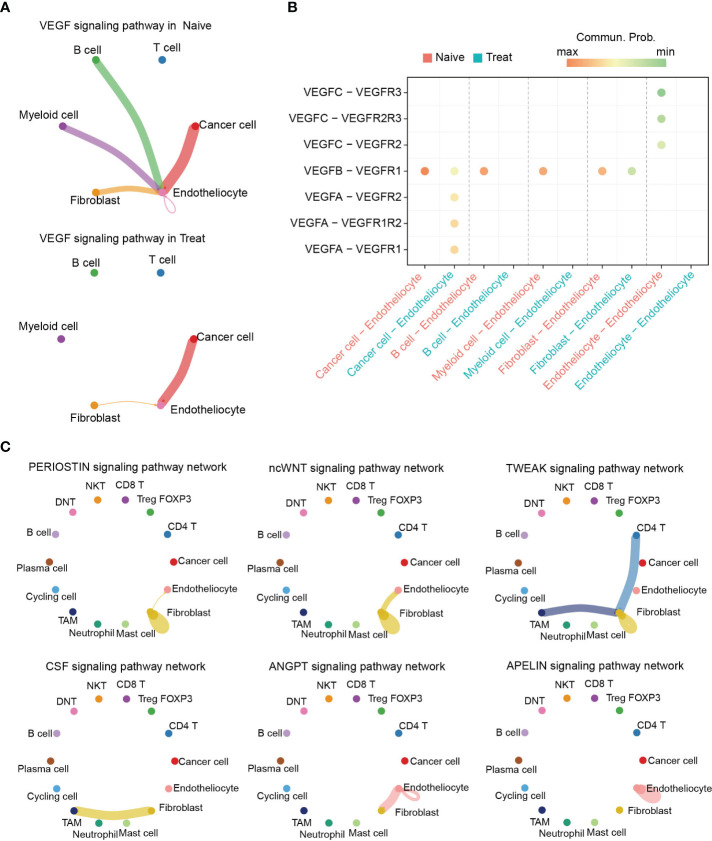
Altered intercellular communication in advanced colon cancer after FOLFOX-Bev treatment. **(A)** Circle plot depicting the inferred VEGF signaling network before (left) and after FOLFOX-Bev treatment (right). Edge colors indicate the sender, and the edge width represents the communication probability. Thicker edge lines indicate stronger signals. **(B)** Bubble chart displaying the ligand and receptor pairs of the VEGF pathway in advanced colon cancer before (red) and after FOLFOX-Bev treatment (green), as calculated by CellChat. The p values were computed using a one-sided permutation test. **(C)** Circle plot showing selected signaling networks present only in the naive sample.

To further investigate the potential effects of FOLFOX-Bev treatment on intercellular communication within the TME, we utilized CellChat for cell communication analysis. Notably, our analysis revealed significant inhibition of the VEGF pathway after treatment (**Figure 7A**), particularly the VEGFB-VEGFR1 pathway which was the primary VEGF pathway in the Naive sample and was markedly suppressed in the Treat sample. Conversely, we observed transcriptional upregulation of the VEGFA-mediated pathway in the Treat sample (**Figures 7B**, **S5A**), indicating that FOLFOX-Bev treatment might have a complex impact on the VEGF pathway.

Further analysis of the TME pathways revealed that PERIOSTIN, ncWNT, TWEAK, CSF, ANGPT, and APELIN pathways were exclusively present in the Naive sample (**Figure S5B**). Moreover, our analysis of the distribution of these pathways among various cell populations (**Figure 7C**) showed that PERIOSTIN, ncWNT, and TWEAK pathways primarily targeted fibroblasts and were involved in promoting fibroblast activation, proliferation, and inducing tissue fibrosis and metastatic niche formation (23–25). The CSF pathway primarily targeted TAMs and may play a role in controlling their proliferation, differentiation, survival, and function (26, 27). Additionally, we found that ncWNT, ANGPT, and APELIN pathways could target endothelial cells, regulating endothelial differentiation and angiogenesis (28–32).

In conclusion, our findings suggest that FOLFOX-Bev treatment not only targets cancer cells but also affects intercellular communication in the TME, leading to inhibition of pathways related to the survival, proliferation, differentiation, and function of non-cancer cells.

To address potential limitations in our study, including small sample size, single-center design, and patient selection bias, we sought to validate our findings by performing additional analyses using an independent publicly available dataset (GSE178318). In this dataset, we focused on untreated (Naïve, n=3) and FOLFOX-Bev-treated (Treat, n=1) colon cancer orthotopic samples. Initially, we annotated the cell types in this dataset using the top differentially expressed genes (DEGs) of the respective cell clusters (**Figures S6A, B**). Subsequently, we classified cancer cells into two subgroups based on their sensitivity to FOLFOX-Bev treatment: a sensitive subgroup (Sensitive) and an insensitive subgroup (Non-sensitive) (**Figure S6C**). Consistent with our original study, we observed that the upregulated genes in the sensitive subgroup, compared to the insensitive subgroup, were significantly enriched in pathways related to proliferation (E2F targets, G2M checkpoint, mitotic spindle) and metastasis (epithelial-mesenchymal transition) (**Figure S6D**). This finding supports the notion that cancer cells exhibiting higher proliferation and metastatic potential are more responsive to FOLFOX-Bev treatment.

Furthermore, we conducted cell cycle analysis and found that FOLFOX-Bev treatment induced G1 cell cycle arrest in cancer cells, as evidenced by an increased proportion of cells in the G1 phase (**Figures S6E, F**). This observation aligns with our original study and strengthens the evidence for the ability of FOLFOX-Bev treatment to influence cell cycle progression in cancer cells. Regarding the tumor-associated macrophages (TAMs), our analysis confirmed that TAMs displayed a relatively quiescent state with reduced functional activity following FOLFOX-Bev treatment, as indicated by scoring the functional characteristics of TAMs (**Figures S6G, H**). This validation further supports our original finding of TAM impairment upon treatment. Additionally, we observed a decrease in the number of cancer-associated fibroblast (CAF) secretory cells and a reduction in the expression of vascular endothelial growth factors (VEGFs) in the Treat sample, suggesting that FOLFOX-Bev treatment may hinder the secretory function of CAFs and potentially impede angiogenesis (**Figures S6I–K**). These results align with our original study and provide further evidence of the impact of FOLFOX-Bev treatment on CAF-mediated angiogenesis

## Discussion

Despite advances in the treatment of advanced CRC, there is limited knowledge about the changes in the TME that occur in response to chemotherapy combined with bevacizumab. In this study, we used scRNA-seq to characterize the cellular landscape of advanced CRC before and after FOLFOX-Bev therapy, encompassing cancer cells, immune cells, and stromal cells. Our findings shed light on the response of specific cell subsets to FOLFOX-Bev therapy, highlighting the potential of this treatment regimen in treating advanced CRC. Importantly, we identified the cancer cell subset with high proliferative, metastatic, and pro-angiogenic properties showed sensitivity to FOLFOX-Bev therapy. Furthermore, we demonstrated that FOLFOX-Bev treatment impaired VEGFB-mediated angiogenesis. These findings have significant implications for the clinical management of advanced CRC and offer insight into the potential use of predictive biomarkers to improve patient outcomes.

Bevacizumab has demonstrated significant preclinical and clinical benefits against metastatic CRC, especially when combined with chemotherapy (33). However, clinical trials investigating the efficacy of adding bevacizumab to FOLFOX in early-stage colon cancer have produced conflicting results. While Allegra et al. reported that the addition of bevacizumab did not significantly prolong disease-free survival in stages II and III colon cancer, despite significant but transient effects being observed (34). Gramont et al. found no benefit for disease-free survival and even a potential detrimental effect for overall survival when bevacizumab was added to adjuvant chemotherapy in stage III colon cancer (35). The reason for the different responses to bevacizumab in metastatic and early-stage CRC remains unclear. In this study, we used scRNA-seq to identify the functional characteristics of FOLFOX-Bev-sensitive cancer cells. We found that these cells exhibited enhanced epithelial-mesenchymal transition and proangiogenic capacity in addition to strong proliferation, which are key factors for the metastasis of CRC. Our findings suggest that CRC with a strong metastatic tendency may be more sensitive to FOLFOX-Bev, which could partly explain why FOLFOX-Bev is more effective in treating metastatic CRC.

The VEGF family plays a critical role in regulating both normal and pathological angiogenesis, which supports the growth, invasion, and metastasis of CRC. The expression of the predominant VEGF subtypes is correlated with the progression of CRC (36, 37). For example, high expression of VEGFB and VEGFC has been found to significantly correlate with lymph node metastasis and lymphatic invasion, while VEGFA is higher in CRC with liver metastasis than in those without (Kawakami et al., 2016) (36). In this study, we observed that VEGFB, secreted by both cancer cells and non-cancer cells, was the predominant VEGF subtype in advanced CRC with multiple metastases to the liver, lung, and perienteric lymph nodes. Bevacizumab is a humanized monoclonal antibody that binds to the VEGFA subtype. Surprisingly, we found that FOLFOX-Bev treatment significantly inhibited the VEGFB-VEGFR1 pathway while enhancing the VEGFA pathway based on transcriptional level. We propose several hypotheses to explain this result. Firstly, FOLFOX-Bev treatment may kill sensitive cancer cells that primarily secrete VEGFB, leading to compensatory upregulation of VEGFA in the remaining insensitive cancer cells, which is competitively bound by bevacizumab, thereby dually inhibiting the VEGF pathway. Additionally, we found that M2 polarization of TAMs was significantly inhibited and remained in a relatively quiescent state after FOLFOX-Bev treatment, resulting in the inhibition of their pro-angiogenic function. Furthermore, we observed that CAF secretory cells, which mainly secrete VEGF, were significantly reduced after FOLFOX-Bev treatment, potentially impairing their angiogenic function. Lastly, we identified a distinct subpopulation of VEGF-secreting plasma cells that were significantly reduced after FOLFOX-Bev treatment. Although the primary function of plasma cells is to synthesize, store, and secrete antibodies, our study and previous research (38) have shown that they can also produce VEGF.

Interestingly, we made an intriguing observation that Treg FOXP3 cells exhibited high expression of cytotoxic T-lymphocyte protein 4 (CTLA-4), a potent molecule known to inhibit T cell activation. CTLA-4 has emerged as a critical immune checkpoint receptor with implications for immune evasion by cancer cells in various cancer types, including CRC (39). Studies by Afshin et al. demonstrated an increasing trend of CTLA-4 expression in CRC tissues and cell lines, and treatment with capecitabine effectively suppressed the expression of CTLA-4 in CRC cells, suggesting a potential bridge between immunotherapy approaches and chemotherapy (40). In addition, multiple research groups have suggested a significant role for CTLA-4 in CRC progression (41, 42), immunosuppression (39), and therapeutic interventions (43). These findings highlight the potential of CTLA-4 as an important target for colon cancer treatment.

There are several important limitations that should be taken into account: i) the findings were based on biopsy samples obtained from a limited number of advanced colon cancer patients before and after FOLFOX-Bev therapy. Although we have obtained some further confirmation through analysis of a published dataset, it is essential to conduct future research with larger and more diverse cohorts to enhance the generalizability of the findings. ii) In terms of cell annotation, we acknowledge the limitations associated with relying solely on canonical cellular markers, as they may not fully capture the complexity and heterogeneity of cell populations. Therefore, we recognize the need for complementary approaches, such as single-cell proteomics or spatial transcriptomics, to provide a more comprehensive and accurate cell characterization. iii) the cell annotation in this study was based on canonical cellular markers from the literature. While these markers provide a useful framework for cell classification, they may not encompass the full complexity and diversity of the cell populations under investigation. Therefore, future studies should aim to incorporate additional markers and employ more comprehensive approaches to capture the full range of cellular heterogeneity. iv) in examining functional differences between cell populations, we utilized predefined functional gene sets derived from previous published papers. However, it is crucial to acknowledge that these gene sets may not encompass the full complexity and intricacies of the biological processes under investigation. Future studies should consider incorporating a broader range of functional gene sets or employ more comprehensive and unbiased methods to explore the functional landscape of the cell populations.

In conclusion, our findings highlight the complex impact of FOLFOX-Bev treatment on the VEGF pathway in advanced CRC. By comprehensively characterizing the cellular composition and transcriptional profiles of the TME in advanced CRC with FOLFOX-Bev therapy, our study provides valuable insights that could aid in the identification of potentially advantageous populations. Moving forward, it is crucial to further investigate and compare the transcriptional profiles among patients exhibiting different responses to FOLFOX-Bev treatment. This approach will enable the identification of clear biomarkers that can guide the selection of patients who are more likely to benefit from FOLFOX-Bev therapy.

## Data availability statement

The data presented in the study are deposited in the NCBI Gene Expression Omnibus database (https://www.ncbi.nlm.nih.gov/), accession number GSE232525 and GSE178318.

## Ethics statement

The studies involving human participants were reviewed and approved by the institutional review boards of Guangdong Provincial People’s Hospital. The patients/participants provided their written informed consent to participate in this study. Written informed consent was obtained from the individual(s) for the publication of any potentially identifiable images or data included in this article.

## Author contributions

MY performed the scRNA-seq analyses, wrote the manuscript and revised the manuscript. CY, DM and ZL interpreted the data and revised the manuscript. WZ and DY interpreted the data, wrote the manuscript, and provided supervision.

## References

[1] Siegel RL Miller KD Fuchs HE Jemal A. Cancer statistics, 2022. CA: A Cancer J Clin (2022) 72(1):7–33. doi: 10.3322/caac.21708 35020204

[2] Vatandoust S Price TJ Karapetis CS. Colorectal cancer: Metastases to a single organ. World J Gastroenterol (2015) 21(41):11767–76. doi: 10.3748/wjg.v21.i41.11767 PMC4631975 26557001

[3] Miller KD Nogueira L Mariotto AB Rowland JH Yabroff KR Alfano CM. Cancer treatment and survivorship statistics, 2019. CA Cancer J Clin (2019) 69(5):363–85. doi: 10.3322/caac.21565 31184787

[4] Ferrara N Hillan KJ Gerber H-P Novotny W. Discovery and development of bevacizumab, an anti-VEGF antibody for treating cancer. Nat Rev Drug Discov (2004) 3(5):391–400. doi: 10.1038/nrd1381 15136787

[5] Li H Courtois ET Sengupta D Tan Y Chen KH Goh JJL. Reference component analysis of single-cell transcriptomes elucidates cellular heterogeneity in human colorectal tumors. Nat Genet (2017) 49(5):708–18. doi: 10.1038/ng.3818 28319088

[6] Wang Q Wang Z Zhang Z Zhang W Zhang M Shen Z. Landscape of cell heterogeneity and evolutionary trajectory in ulcerative colitis-associated colon cancer revealed by single-cell RNA sequencing. Chin J Cancer Res (2021) 33(2):271–88. doi: 10.21147/j.issn.1000-9604.2021.02.13 PMC8181874 34158745

[7] Uhlitz F Bischoff P Peidli S Sieber A Trinks A Lüthen M. Mitogen-activated protein kinase activity drives cell trajectories in colorectal cancer. EMBO Mol Med (2021) 13(10):e14123. doi: 10.15252/emmm.202114123 34409732 PMC8495451

[8] Lee HO Hong Y Etlioglu HE Cho YB Pomella V Van den Bosch B. Lineage-dependent gene expression programs influence the immune landscape of colorectal cancer. Nat Genet (2020) 52(6):594–603. doi: 10.1038/s41588-020-0636-z 32451460

[9] Liu Y Zhang Q Xing B Luo N Gao R Yu K. Immune phenotypic linkage between colorectal cancer and liver metastasis. Cancer Cell (2022) 40(4):424–37.e5. doi: 10.1016/j.ccell.2022.02.013 35303421

[10] Che LH Liu JW Huo JP Luo R Xu RM He C. A single-cell atlas of liver metastases of colorectal cancer reveals reprogramming of the tumor microenvironment in response to preoperative chemotherapy. Cell Discov (2021) 7(1):80. doi: 10.1038/s41421-021-00312-y 34489408 PMC8421363

[11] Zhang L Li Z Skrzypczynska KM Fang Q Zhang W O'Brien SA. Single-cell analyses inform mechanisms of myeloid-targeted therapies in colon cancer. Cell (2020) 181(2):442–59.e29. doi: 10.1016/j.cell.2020.03.048 32302573

[12] Yang M Wang F Lu G Cheng M Zhao W Zou C. Single-cell transcriptome analysis reveals T-cell exhaustion in denosumab-treated giant cell tumor of bone. Front Immunol (2022) 13:934078. doi: 10.3389/fimmu.2022.934078 36172351 PMC9510370

[13] Yang M Wang F Liang H Ji G Lian Y Zou C. Single-cell RNA sequencing reveals distinct immune cell subsets in phalangeal and soft-tissue recurrence of giant cell tumor of bone. Med Adv (2023) 1(1):14–29. doi: 10.1002/med4.10

[14] Hao Y Hao S Andersen-Nissen E Mauck WM 3rd Zheng S Butler A. Integrated analysis of multimodal single-cell data. Cell (2021) 184(13):3573–87 e29. doi: 10.1016/j.cell.2021.04.048 34062119 PMC8238499

[15] Sun Y Wu L Zhong Y Zhou K Hou Y Wang Z. Single-cell landscape of the ecosystem in early-relapse hepatocellular carcinoma. Cell (2021) 184(2):404–21 e16. doi: 10.1016/j.cell.2020.11.041 33357445

[16] Zhou Y Yang D Yang Q Lv X Huang W Zhou Z. Single-cell RNA landscape of intratumoral heterogeneity and immunosuppressive microenvironment in advanced osteosarcoma. Nat Commun (2020) 11(1):6322. doi: 10.1038/s41467-020-20059-6 33303760 PMC7730477

[17] Yu G Wang LG Han Y He QY. clusterProfiler: an R package for comparing biological themes among gene clusters. OMICS (2012) 16(5):284–7. doi: 10.1089/omi.2011.0118 PMC3339379 22455463

[18] Liberzon A Birger C Thorvaldsdóttir H Ghandi M Mesirov JP Tamayo P. The Molecular Signatures Database (MSigDB) hallmark gene set collection. Cell Syst (2015) 1(6):417–25. doi: 10.1016/j.cels.2015.12.004 PMC4707969 26771021

[19] Trapnell C Cacchiarelli D Grimsby J Pokharel P Li S Morse M. The dynamics and regulators of cell fate decisions are revealed by pseudotemporal ordering of single cells. Nat Biotechnol (2014) 32(4):381–6. doi: 10.1038/nbt.2859 PMC4122333 24658644

[20] Qiu X Hill A Packer J Lin D Ma YA Trapnell C. Single-cell mRNA quantification and differential analysis with Census. Nat Methods (2017) 14(3):309–15. doi: 10.1038/nmeth.4150 PMC5330805 28114287

[21] Qiu X Mao Q Tang Y Wang L Chawla R Pliner HA. Reversed graph embedding resolves complex single-cell trajectories. Nat Methods (2017) 14(10):979–82. doi: 10.1038/nmeth.4402 PMC5764547 28825705

[22] Jin SQ Guerrero-Juarez CF Zhang LH Chang I Ramos R Kuan CH. Inference and analysis of cell-cell communication using CellChat. Nat Commun (2021) 12(1):1088. doi: 10.1038/s41467-021-21246-9 33597522 PMC7889871

[23] Kong J Tian H Zhang F Zhang Z Li J Liu X. Extracellular vesicles of carcinoma-associated fibroblasts creates a pre-metastatic niche in the lung through activating fibroblasts. Mol Cancer (2019) 18(1):175. doi: 10.1186/s12943-019-1101-4 31796058 PMC6892147

[24] Vuga LJ Ben-Yehudah A Kovkarova-Naumovski E Oriss T Gibson KF Feghali-Bostwick C. WNT5A is a regulator of fibroblast proliferation and resistance to apoptosis. Am J Respir Cell Mol Biol (2009) 41(5):583–9. doi: 10.1165/rcmb.2008-0201OC PMC2778165 19251946

[25] Zhang Y Zeng W Xia Y. TWEAK/Fn14 axis is an important player in fibrosis. J Cell Physiol (2021) 236(5):3304–16. doi: 10.1002/jcp.30089 33000480

[26] Neubert NJ Schmittnaegel M Bordry N Nassiri S Wald N Martignier C. T cell-induced CSF1 promotes melanoma resistance to PD1 blockade. Sci Trans Med (2018) 10(436):eaan3311. doi: 10.1126/scitranslmed.aan3311 PMC5957531 29643229

[27] Hume DA MacDonald KP. Therapeutic applications of macrophage colony-stimulating factor-1 (CSF-1) and antagonists of CSF-1 receptor (CSF-1R) signaling. Blood (2012) 119(8):1810–20. doi: 10.1182/blood-2011-09-379214 22186992

[28] Chen T Zhang F Liu J Huang Z Zheng Y Deng S. Dual role of WNT5A in promoting endothelial differentiation of glioma stem cells and angiogenesis of glioma derived endothelial cells. Oncogene (2021) 40(32):5081–94. doi: 10.1038/s41388-021-01922-2 34188250

[29] Yang DH Yoon JY Lee SH Bryja V Andersson ER Arenas E. Wnt5a is required for endothelial differentiation of embryonic stem cells and vascularization via pathways involving both Wnt/beta-catenin and protein kinase Calpha. Circ Res (2009) 104(3):372–9. doi: 10.1161/circresaha.108.185405 19096028

[30] Aoi J Endo M Kadomatsu T Miyata K Nakano M Horiguchi H. Angiopoietin-like protein 2 is an important facilitator of inflammatory carcinogenesis and metastasis. Cancer Res (2011) 71(24):7502–12. doi: 10.1158/0008-5472.can-11-1758 22042794

[31] Endo M Nakano M Kadomatsu T Fukuhara S Kuroda H Mikami S. Tumor cell-derived angiopoietin-like protein ANGPTL2 is a critical driver of metastasis. Cancer Res (2012) 72(7):1784–94. doi: 10.1158/0008-5472.can-11-3878 22345152

[32] Helker CS Eberlein J Wilhelm K Sugino T Malchow J Schuermann A. Apelin signaling drives vascular endothelial cells toward a pro-angiogenic state. Elife (2020) 9:e55589. doi: 10.7554/eLife.55589 32955436 PMC7567607

[33] Hurwitz H Fehrenbacher L Novotny W Cartwright T Hainsworth J Heim W. Bevacizumab plus irinotecan, fluorouracil, and leucovorin for metastatic colorectal cancer. New Engl J Med (2004) 350(23):2335–42. doi: 10.1056/NEJMoa032691 15175435

[34] Allegra CJ Yothers G O'Connell MJ Sharif S Petrelli NJ Colangelo LH. Phase III trial assessing bevacizumab in stages II and III carcinoma of the colon: results of NSABP protocol C-08. J Clin Oncol Off J Am Soc Clin Oncol (2011) 29(1):11–6. doi: 10.1200/JCO.2010.30.0855 PMC3055856 20940184

[35] de Gramont A Van Cutsem E Schmoll HJ Tabernero J Clarke S Moore MJ. Bevacizumab plus oxaliplatin-based chemotherapy as adjuvant treatment for colon cancer (AVANT): a phase 3 randomised controlled trial. Lancet Oncol (2012) 13(12):1225–33. doi: 10.1016/S1470-2045(12)70509-0 23168362

[36] Kawakami M Furuhata T Kimura Y Yamaguchi K Hata F Sasaki K. Expression analysis of vascular endothelial growth factors and their relationships to lymph node metastasis in human colorectal cancer. J Exp Clin Cancer Res CR (2003) 22(2):229–37. 12866573

[37] Hanrahan V Currie MJ Gunningham SP Morrin HR Scott PA Robinson BA. The angiogenic switch for vascular endothelial growth factor (VEGF)-A, VEGF-B, VEGF-C, and VEGF-D in the adenoma-carcinoma sequence during colorectal cancer progression. J Pathol (2003) 200(2):183–94. doi: 10.1002/path.1339 12754739

[38] Kumar S Witzig TE Timm M Haug J Wellik L Fonseca R. Expression of VEGF and its receptors by myeloma cells. Leukemia (2003) 17(10):2025–31. doi: 10.1038/sj.leu.2403084 14513053

[39] Imazeki H Ogiwara Y Kawamura M Boku N Kudo-Saito C. CD11b(+)CTLA4(+) myeloid cells are a key driver of tumor evasion in colorectal cancer. J Immunother Cancer (2021) 9(7):e002841. doi: 10.1136/jitc-2021-002841 34261702 PMC8280900

[40] Derakhshani A Hashemzadeh S Asadzadeh Z Shadbad MA Rasibonab F Safarpour H. Cytotoxic T-Lymphocyte Antigen-4 in colorectal cancer: another therapeutic side of capecitabine. Cancers (2021) 13(10):2414. doi: 10.3390/cancers13102414 34067631 PMC8155910

[41] Li J Chen Y Liao M Yu S Yuan B Jia Z. Exosomes-delivered PD-L1 siRNA and CTLA-4 siRNA protect against growth and tumor immune escape in colorectal cancer. Genomics (2023) 115(4):110646. doi: 10.1016/j.ygeno.2023.110646 37217085

[42] Omura Y Toiyama Y Okugawa Y Yin C Shigemori T Kusunoki K. Prognostic impacts of tumoral expression and serum levels of PD-L1 and CTLA-4 in colorectal cancer patients. Cancer Immunol Immunother CII (2020) 69(12):2533–46. doi: 10.1007/s00262-020-02645-1 PMC11027465 32577816

[43] Kanani A Veen T Søreide K. Neoadjuvant immunotherapy in primary and metastatic colorectal cancer. Br J Surg (2021) 108(12):1417–25. doi: 10.1093/bjs/znab342 PMC10364874 34694371

