# Supplementary material for: Single-cell analysis reveals cellular reprogramming in advanced colon cancer following FOLFOX-bevacizumab treatment
Source: Front Oncol. 2023 Jul 28;13:1219642. doi: 10.3389/fonc.2023.1219642 (PMC10421721; doi:10.3389/fonc.2023.1219642)
Supplement: Supplementary file 1 [file DataSheet_1.docx]

https://www.jianguoyun.com/p/DZOtGVkQ3K7RCxjp_YUFIAA
