# Supplementary material for: Single-cell analysis reveals cellular reprogramming in advanced colon cancer following FOLFOX-bevacizumab treatment
Source: Front Oncol. 2023 Jul 28;13:1219642. doi: 10.3389/fonc.2023.1219642 (PMC10421721; doi:10.3389/fonc.2023.1219642)
Supplement: Supplementary file 2 [file DataSheet_2.docx]

Supplementary Material

Single-Cell Analysis Reveals Cellular Reprogramming in Advanced Colon Cancer following FOLFOX-Bevacizumab Treatment

Meiling Yang, Ciqiu Yang, Dong Ma, Zijun Li, Wei Zhao*, Dongyang Yang*

*** Correspondence:**

Wei Zhao

Email: zhaowei23@mail.sysu.edu.cn

Dongyang Yang

Email: 845419316@qq.com

# Supplementary Figures


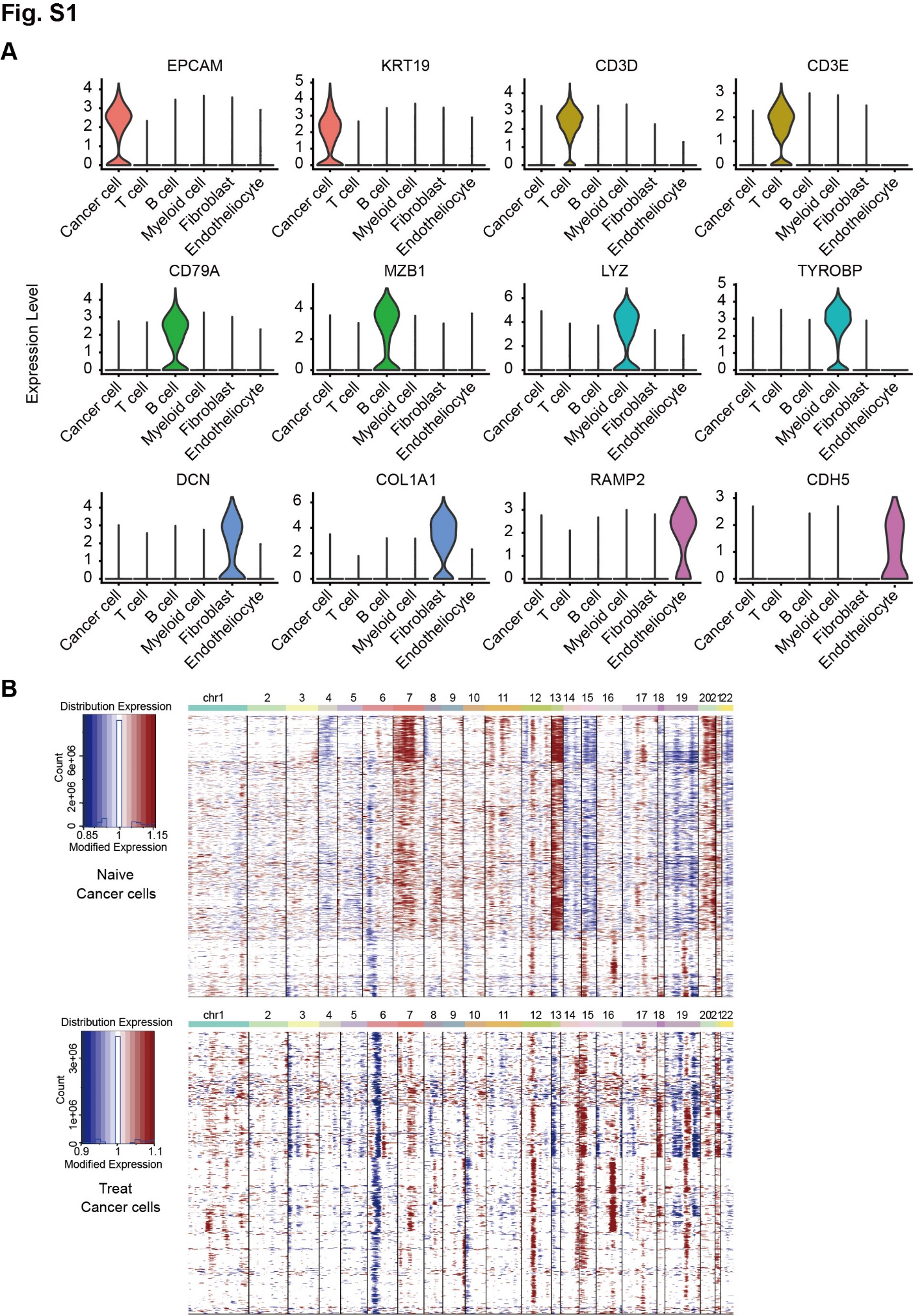


**Single-cell RNA sequencing (scRNA-seq) profiling of the tumor microenvironment (TME) in advanced colon cancer before and after FOLFOX-Bev treatment, related to Figure 1.** (A) Violin plot illustrating the expression levels of marker genes in different cell types within the TME. (B) Heatmap showcasing large-scale copy number variations (CNVs) identified in the cancer cells based on the scRNA-seq data. The heatmap displays regions of amplifications in red and deletions in blue.


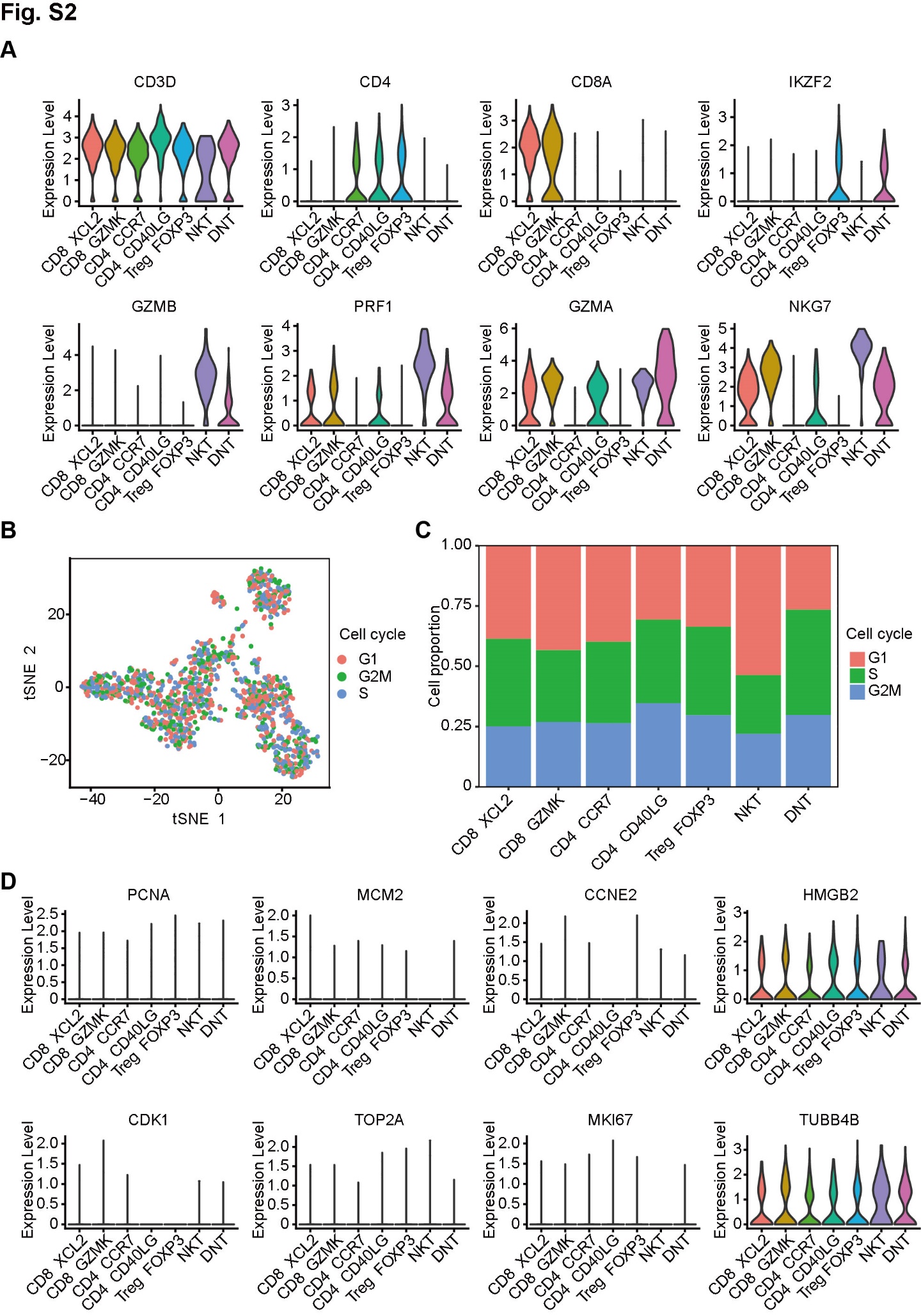


**Supplementary Figure 2. Identification of double negative T cell cluster (DNT) in advanced colon cancer, related to Figure 4.** (A) Violin plot illustrating the expression levels of selected marker genes in different T cell subtypes, including the double negative T cell cluster (DNT). (B) t-SNE plot visualizing the spatial distribution of T cells in different cell-cycle phases. The t-SNE plot helps to identify the organization and clustering patterns of T cells based on their cell-cycle phases. (C) Bar plots presenting the cell proportion of T cell subtypes within different cell-cycle phases. (D) Violin plot displaying the expression levels of cell cycle-related genes within the identified T cell subtypes.


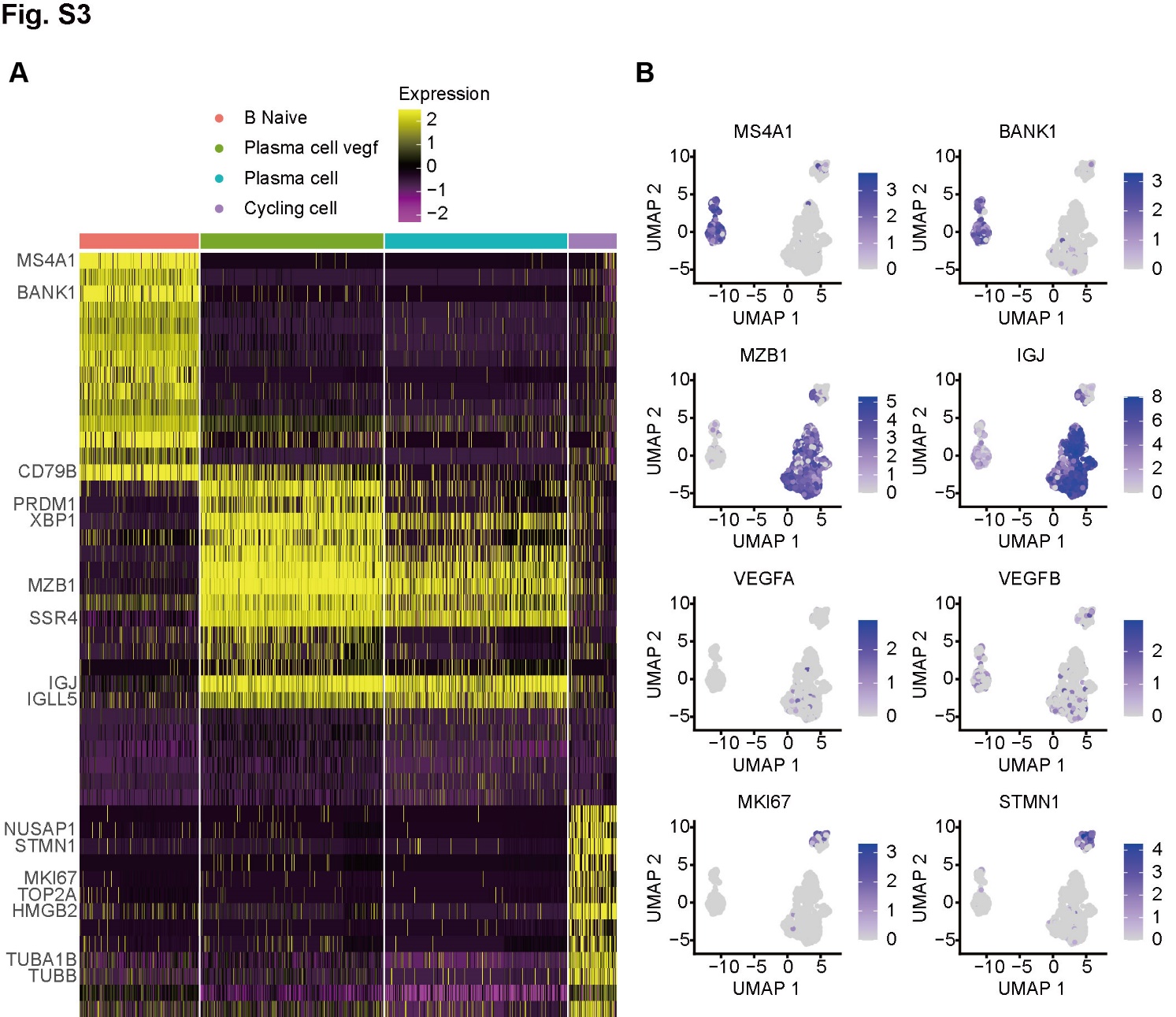


**Supplementary Figure 3. Cell clustering of B cells in advanced colon cancer, related to Figure 5.** (A) Heatmap displaying the top 20 differentially expressed genes (DEGs) of the identified B cell subtypes. Each row represents a gene, and each column represents a single cell. (B) UMAP plot visualizing the expression levels of selected marker genes within the B cell population.


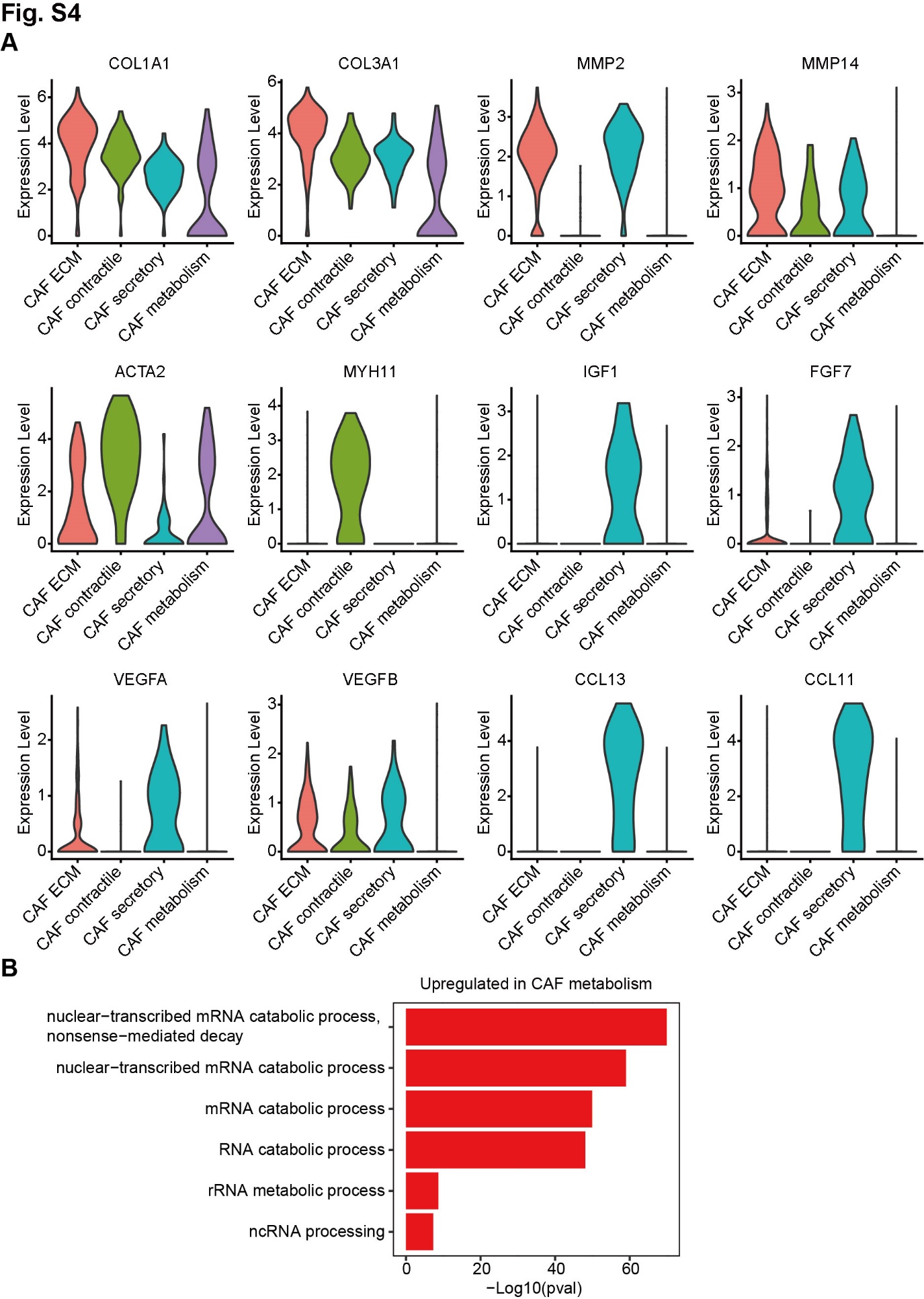


**Supplementary Figure 4. Identification of cancer-associated fibroblast (CAF) subtypes in advanced colon cancer, related to Figure 6.** (A) Violin plot illustrating the expression levels of selected marker genes within the identified CAF subtypes. (B) Gene Ontology (GO) enrichment analysis revealing that CAF metabolism cells were significantly enriched in pathways associated with RNA metabolism.


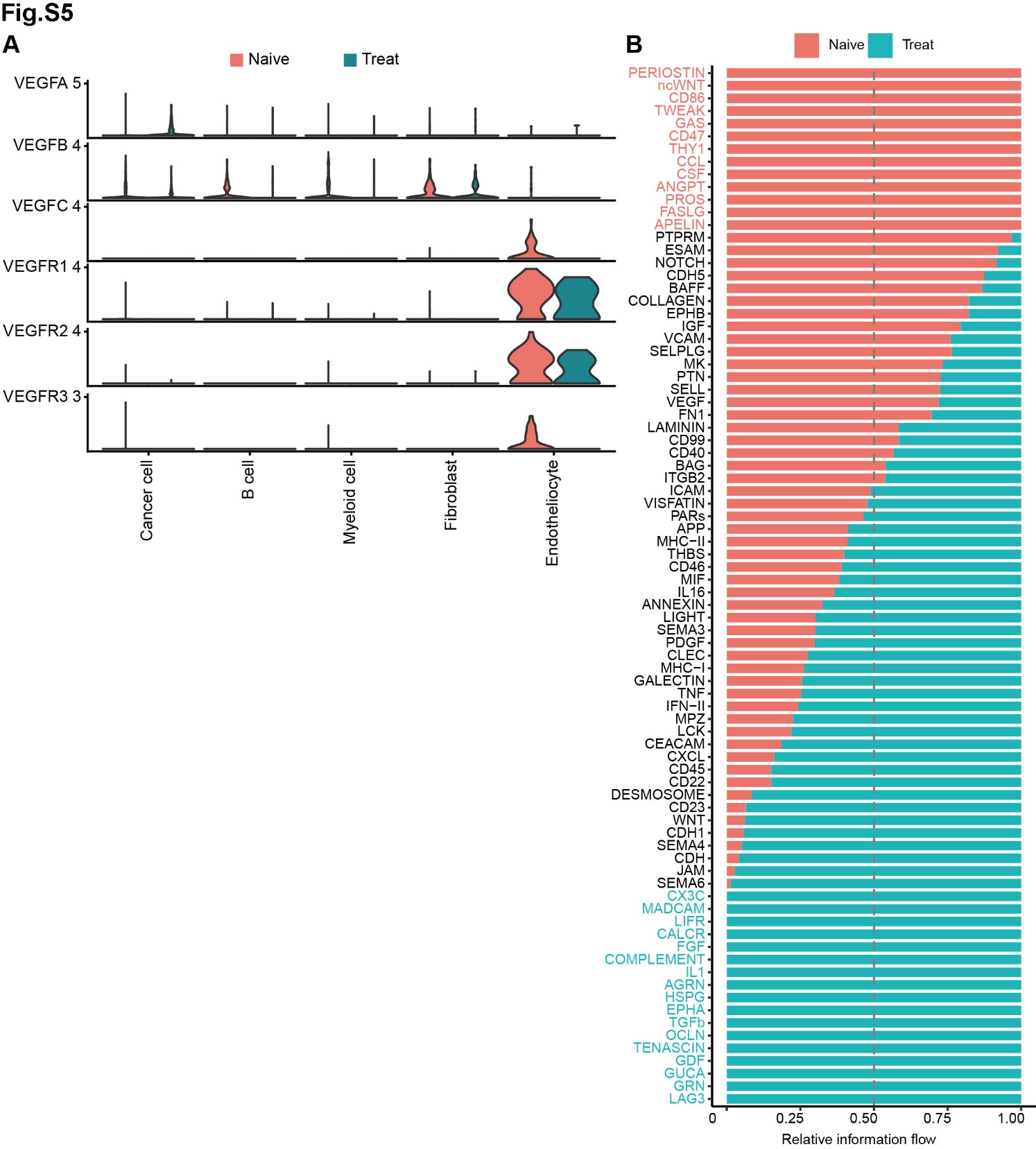


**Supplementary Figure 5. Altered intercellular communication in advanced colon cancer after FOLFOX-Bev treatment, related to Figure 7.** (A) Violin plot illustrating the expression levels of genes involved in the VEGF signaling network before (Naive) and after (Treat) FOLFOX-Bev treatment. (B) Signaling pathways ranked based on their differences in the overall information flow within the inferred networks between Naive and Treat. The top signaling pathways, colored in red, are enriched in Naive, while the middle ones, colored in black, are enriched in both Naive and Treat. The bottom signaling pathways, colored in green, are enriched in Treat. The significance of the differences in pathway enrichment was assessed using a permutation test.


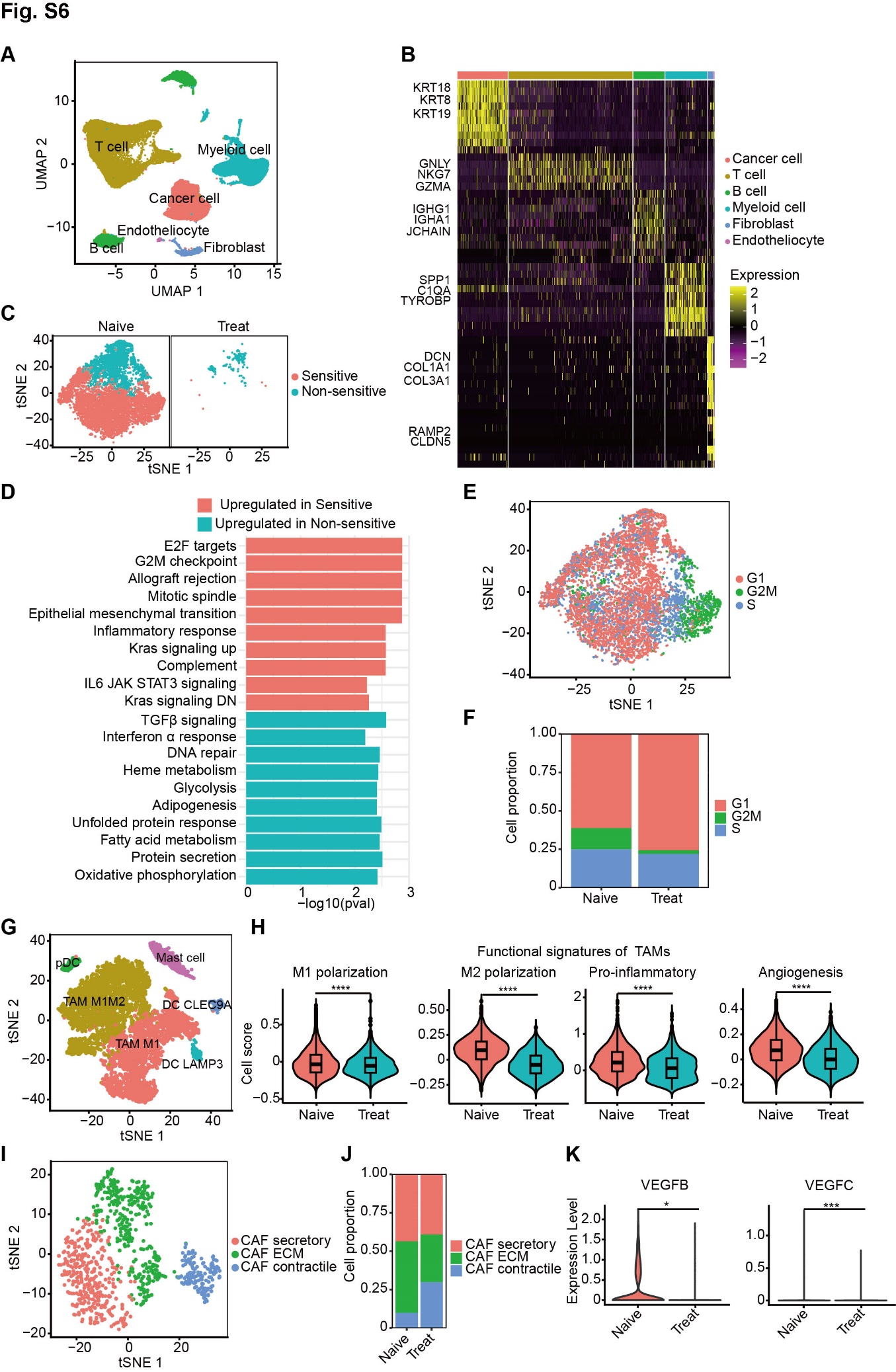


**Supplementary Figure 6.** **Validation of important conclusions with the published dataset (GSE178318).** (A) UMAP plot displaying the indicated cell types in advanced colon cancer. (B) Heatmap showing the top 10 DEGs of the indicated cell types. The heatmap represents the expression levels of the top 10 DEGs in each cell type. (C) t-SNE plot showing the distribution of cancer cell subpopulations in advanced colon cancer before (left, Naïve, n=3) and after FOLFOX-Bev treatment (right, Treat, n=1). The t-SNE plot allows for the comparison of cancer cell subpopulations between the Naive and Treat conditions, assessing the impact of FOLFOX-Bev treatment on cancer cell heterogeneity. (D) Gene Set Enrichment Analysis (GSEA) of upregulated genes in sensitive or non-sensitive malignant cells, based on the HALLMARK gene set. (E) t-SNE plot showing the distribution of cancer cells in different cell-cycle phases. (F) Bar plots depicting the cell proportion of cancer cells in different cell-cycle phases before and after FOLFOX-Bev treatment. (G) t-SNE plot depicting the subtypes of myeloid-derived cells in advanced colon cancer. (H) Violin plot showing the M1/M2 polarization, pro-inflammatory, and angiogenesis score of TAMs. (I) t-SNE plot depicting the subtypes of cancer-associated fibroblasts (CAFs) in advanced colon cancer. (J) Bar plots depicting the cell proportion of each CAF subtype before and after FOLFOX-Bev treatment. (K) Violin plot showing the expression levels of VEGFB and VEGFC in CAFs before and after FOLFOX-Bev treatment. The statistical significance was assessed using a Student's t-test. The p-values are denoted as follows: *, p < 0.05; ***, p ≤ 0.001; ****, p ≤ 0.0001.
