# Supplementary material for: Single-cell analysis reveals cellular reprogramming in advanced colon cancer following FOLFOX-bevacizumab treatment
Source: Front Oncol. 2023 Jul 28;13:1219642. doi: 10.3389/fonc.2023.1219642 (PMC10421721; doi:10.3389/fonc.2023.1219642)
Supplement: Supplementary file 3 [file DataSheet_1.zip › PDF/Figure1 and figureS1.pdf]

```
library(Seurat)
library(dplyr)
library(patchwork)
library(tidyverse)
library(stringr)
library(ggpubr)

#本数据是同一个结肠癌患者（晚期，发生肝转移）FOLFOX 化疗+贝伐单抗（VEGFA 的单抗)靶向治疗前后的配对结肠病理样本的单细胞数据

#以下是参照 seurat V4 工作流程进行的整合分析
Naive.data <- Read10X(data.dir = "F:/scRNA/JCML/raw data/JCML1/")
Naive <- CreateSeuratObject(counts = Naive.data,min.cells = 10, min.features = 200, project = "Naive")
Naive[["percent.mt"]] <- PercentageFeatureSet(Naive, pattern = "^MT-")
Naive[["percent.rb"]] <- PercentageFeatureSet(Naive, pattern = "^RP[SL]")
VlnPlot(Naive, features = c("nFeature_RNA", "nCount_RNA", "percent.mt","percent.rb"), ncol = 2)
Naive <- subset(Naive, subset = nFeature_RNA > 200 & nFeature_RNA < 6000 & percent.mt <50)
Naive

Treat.data <- Read10X(data.dir = "F:/scRNA/JCML/raw data/JCML2/")
Treat <- CreateSeuratObject(counts = Treat.data,min.cells = 10, min.features = 200, project = "Treat")
Treat[["percent.mt"]] <- PercentageFeatureSet(Treat, pattern = "^MT-")
Treat[["percent.rb"]] <- PercentageFeatureSet(Treat, pattern = "^RP[SL]")
VlnPlot(Treat, features = c("nFeature_RNA", "nCount_RNA", "percent.mt","percent.rb"), ncol = 2)
Treat <- subset(Treat, subset = nFeature_RNA > 200 & nFeature_RNA < 6000 & percent.mt <50)
Treat

#
JCML <- merge(Naive, y = Treat, add.cell.ids = c("Naive","Treat"), project = "JCML")
JCML

head(colnames(JCML))
tail(colnames(JCML))
table(JCML$orig.ident)
# 查看合并的元数据
```

```
View(JCML@meta.data)

# split the dataset into a list of two seurat objects (Naive and Treat)
JCML.list <- SplitObject(JCML, split.by = "orig.ident")
# normalize and identify variable features for each dataset
independently
JCML.list <- lapply(X = JCML.list, FUN = function(x) {
  x <- NormalizeData(x)
  x <- FindVariableFeatures(x, selection.method = "vst", nfeatures =
2000)
})

# select features that are repeatedly variable across datasets for
integration
features <- SelectIntegrationFeatures(object.list = JCML.list)

### Integration ----
JCML.anchors <- FindIntegrationAnchors(object.list = JCML.list,
anchor.features = features)

# this command creates an 'integrated' data assay
JCML.combined <- IntegrateData(anchorset = JCML.anchors)
JCML.combined
saveRDS(JCML.combined,file="F:/scRNA/JCML/analysis3/JCML_combined.RDS")
JCML.combined <-
readRDS(file="F:/scRNA/JCML/analysis3/JCML_combined.RDS")


# specify that we will perform downstream analysis on the corrected data
note that the original
# unmodified data sNKTl resides in the 'RNA' assay
DefaultAssay(JCML.combined) <- "integrated"

# Run the standard workflow for visualization and clustering
JCML.combined <- ScaleData(JCML.combined, verbose = FALSE)
JCML.combined <- RunPCA(JCML.combined,verbose = FALSE)
ElbowPlot(JCML.combined)

JCML.combined <- RunUMAP(JCML.combined, reduction = "pca", dims = 1:20)
JCML.combined <- RunTSNE(JCML.combined, reduction = "pca", dims = 1:20)
JCML.combined <- FindNeighbors(JCML.combined, reduction = "pca", dims =
1:20)
saveRDS(JCML.combined,file="F:/scRNA/JCML/analysis3/JCML_combined_20.R
DS")
```

```
JCML.combined <-
readRDS(file="F:/scRNA/JCML/analysis3/JCML_combined_20.RDS")

JCML.combined <- FindClusters(JCML.combined, resolution = 2)
saveRDS(JCML.combined,file="F:/scRNA/JCML/analysis3/JCML_combined_20_2
.RDS")
JCML.combined <-
readRDS(file="F:/scRNA/JCML/analysis3/JCML_combined_20_2.RDS")

table(Idents(JCML.combined))
# Visualization
p1 <- DimPlot(JCML.combined, reduction = "umap", group.by =
"orig.ident")+theme(panel.background =
element_blank(),panel.grid.major = element_blank(),panel.border =
element_rect(colour="black",fill=NA))
p2 <- DimPlot(JCML.combined, reduction = "umap", label = TRUE, repel =
TRUE)+theme(panel.background = element_blank(),panel.grid.major =
element_blank(),panel.border = element_rect(colour="black",fill=NA))
p1 + p2

p3 <- DimPlot(JCML.combined, reduction = "tsne", group.by =
"orig.ident")+theme(panel.background =
element_blank(),panel.grid.major = element_blank(),panel.border =
element_rect(colour="black",fill=NA))
p4 <- DimPlot(JCML.combined, reduction = "tsne",label = TRUE, repel =
TRUE,pt.size=1)+theme(panel.background =
element_blank(),panel.grid.major = element_blank(),panel.border =
element_rect(colour="black",fill=NA))
p3 + p4


#To visualize the two conditions side-by-side, we can use the split.by
argument to show each condition colored by cluster.

DimPlot(JCML.combined, reduction = "umap", split.by =
"orig.ident",label = TRUE)+theme(panel.background =
element_blank(),panel.grid.major = element_blank(),panel.border =
element_rect(colour="black",fill=NA))
DimPlot(JCML.combined, reduction = "tsne", split.by =
"orig.ident",label = TRUE)+theme(panel.background =
element_blank(),panel.grid.major = element_blank(),panel.border =
element_rect(colour="black",fill=NA))
```

```
#We can explore these marker genes for each cluster and use them to annotate our clusters as specific cell types.
DefaultAssay(JCML.combined) <- "RNA"


#Epithelial cell/cancer cell
plots <- VlnPlot(JCML.combined, features = c("EPCAM","KRT19","KRT18","CDH1","KRT5","KRT14","KRT15","KRT16"),
                 pt.size = 0, combine = FALSE)
wrap_plots(plots = plots, ncol = 1)

VlnPlot(JCML.combined, features = c("MKI67","PCNA","TOP2A"),pt.size = 0)

#NKT tumor-infiltration lymphocyte:T CELL:CD3D,CD3E,CD3G;NK:NKG7,GNLY;B CELL:MS4A1,CD19,CD79A
plots <- VlnPlot(JCML.combined, features = c("CD3D","CD3E","CD2", "NKG7", "GNLY","MS4A1","CD79A","MZB1","CD19"),
                 pt.size = 0, combine = FALSE)
wrap_plots(plots = plots, ncol = 1)
VlnPlot(JCML.combined, features = c("CD8A","CD8B","CD4","CTLA4","HAVCR2","LAG3","TIGIT","PDCD1"),pt.size = 0)


#Plasma Cell Markers:TNFRSF17/BCMA;SDC1/CD138,CD38
plots <- VlnPlot(JCML.combined, features = c("TNFRSF17","CD27","CD38", "CCR4","SDC1",

"IGHG1","IGHG2","IGHG3","IGHG4","IGHD","IGHM"),
                 pt.size = 0, combine = FALSE)
wrap_plots(plots = plots, ncol = 1)

#Myeloid cell
plots <- VlnPlot(JCML.combined, features = c("CD74", "CD14", "FCGR3A","LYZ","CD68","TYROBP","PTPRC"),
                 pt.size = 0, combine = FALSE)
wrap_plots(plots = plots, ncol = 1)
#DC
plots <- VlnPlot(JCML.combined, features = c("CD1A","CD1C","FCER1A","CLEC9A","CCR7","CD14","CD163"),
                 pt.size = 0, combine = FALSE)
wrap_plots(plots = plots, ncol = 1)
#neutrophils characterized by S100A8, S100A9, and G0S2
VlnPlot(JCML.combined, features = c("CSF3R","S100A8",
```

```
"S100A9","G0S2","MPO","CD15","FUT4","CD32",

"CD66b","CEACAM8","SELL","ELANE","BPI"),pt.size = 0)

#MAST CELL
plots <- VlnPlot(JCML.combined, features = c("KIT","MS4A2","GATA2","IL2RA","PTPRC","FCER2","TPSAB1","FCER1A"),
                 pt.size = 0, combine = FALSE)
wrap_plots(plots = plots, ncol = 1)
#Fibroblast
plots <- VlnPlot(JCML.combined, features = c("DCN", "COL1A1", "C1R","LUM","FN1","VIM","THY1"),
                 pt.size = 0, combine = FALSE)
wrap_plots(plots = plots, ncol = 1)
FeaturePlot(JCML.combined, features = c("DCN", "COL1A1", "LUM","FN1","VIM","THY1"),reduction = "tsne")

#CAF
plots <- VlnPlot(JCML.combined, features = c("FAP","ACTA2","ITGB1","S100A4","CAV1","PDGFRB","PDGFRA","PDPN","MCAM"),
                 pt.size = 0, combine = FALSE)
wrap_plots(plots = plots, ncol = 1)
VlnPlot(JCML.combined, features = c("FAP","ACTA2","ITGB1","S100A4","CAV1","PDGFRB","PDGFRA","PDPN","MCAM"),
        pt.size = 0)
#Myoblast
plots <- VlnPlot(JCML.combined, features = c("MYLPF","MYL1"),
                 pt.size = 0, combine = FALSE)
wrap_plots(plots = plots, ncol = 1)

# Mesenchymal stem cell markers:CD166/ALCAM, CD73/NT5E,CD105/ENG,BST1/stromal cell antigen 1 /stro-1,"MME","THY1","CXCL12","SFRP2"
plots <- VlnPlot(JCML.combined, features = c("ALCAM","NT5E","ENG","BST1","MME","THY1","CXCL12","SFRP2"),
                 pt.size = 0, combine = FALSE)
wrap_plots(plots = plots, ncol = 1)

#Endotheliocyte
plots <- VlnPlot(JCML.combined, features = c("PECAM1","VWF","RAMP2","CD34","DARC","CDH5"),
                 pt.size = 0, combine = FALSE)
```

```
wrap_plots(plots = plots, ncol = 1)
#Pericytes:MCAM/CD146,CSPG4/NG2,ALPL/Alkaline phosphatase
plots <- VlnPlot(JCML.combined, features = c("RGS5","ACTA2","MCAM","CSPG4","ALPL"),
                 pt.size = 0, combine = FALSE)
wrap_plots(plots = plots, ncol = 1)

#cancer stem cell marker:CD133/PROM1,CD44,OCT3/POU5F1/OCT4,SOX2,NANOG,MET/C-MET,CD24,ALDH1A1,BST1/stromal cell antigen 1 /stro-1
plots <- VlnPlot(JCML.combined, features = c("PROM1","CD44","POU5F1","SOX2","NANOG","MET","CD24","ALDH1A1"),
                 pt.size = 0, combine = FALSE)
wrap_plots(plots = plots, ncol = 1)

VlnPlot(JCML.combined, features = c("HBB","HBA"),pt.size = 0)

VlnPlot(JCML.combined, features = c("nFeature_RNA", "nCount_RNA", "percent.mt", "percent.rb"),split.by = "orig.ident", ncol = 2)

VlnPlot(JCML.combined, features = c("nFeature_RNA", "nCount_RNA", "percent.mt", "percent.rb"),ncol = 2)


#20 2
#C15:高表达核糖体,线粒体相关基因
C15 <- FindMarkers(JCML.combined, ident.1 =15)
write.csv(C15,file="F:/scRNA/JCML/analysis3/20 2/C15_markers.csv")
#C11:高表达核糖体相关基因
C11 <- FindMarkers(JCML.combined, ident.1 =11)
write.csv(C11,file="F:/scRNA/JCML/analysis3/20 2/C11_markers.csv")


#细胞类型注释
JCML.combined <- readRDS(file="F:/scRNA/JCML/analysis3/JCML_combined_20_2.RDS")
DimPlot(JCML.combined, reduction = "tsne",label = TRUE, repel = TRUE,pt.size=1)+theme(panel.background = element_blank(),panel.grid.major = element_blank(),panel.border = element_rect(colour="black",fill=NA))
DimPlot(JCML.combined, reduction = "umap",label = TRUE, repel = TRUE,pt.size=1)+theme(panel.background = element_blank(),panel.grid.major = element_blank(),panel.border = element_rect(colour="black",fill=NA))
```

```
JCML.combined@meta.data
#细胞类型
Myeloid_cell=c(9,12,18,22,24,29)
Cancer_cell=c(0,6,8,10,16,17,21,26,27,30)
Fibroblast=c(14,19,28,32)
T_cell=c(1,2,5,7,20)
B_cell=c(3,4,13,23)
Endotheliocyte=c(31)
Unknown=c(11,15)
Doublets=c(25)


current.cluster.ids                                                    <-
c(Myeloid_cell,Cancer_cell,Fibroblast,T_cell,B_cell,Endotheliocyte,Unk
nown,Doublets)
new.cluster.ids <- c(rep("Myeloid_cell",length(Myeloid_cell)),
                     rep("Cancer_cell",length(Cancer_cell)),
                     rep("Fibroblast",length(Fibroblast)),
                     rep("T_cell",length(T_cell)),
                     rep("B_cell",length(B_cell)),
                     rep("Endotheliocyte",length(Endotheliocyte)),
                     rep("Unknown",length(Unknown)),
                     rep("Doublets",length(Doublets)))

JCML.combined@meta.data$Cluster        <-        plyr::mapvalues(x        =
as.integer(as.character(JCML.combined@meta.data$seurat_clusters)),
from = current.cluster.ids, to = new.cluster.ids)
head(JCML.combined@meta.data)
table(JCML.combined@meta.data$Cluster)


JCML.combined$Cluster                                                  <-
factor(JCML.combined$Cluster,level=c("Cancer_cell",

"T_cell","B_cell","Myeloid_cell",

"Fibroblast","Endotheliocyte",

"Unknown","Doublets"))
Idents(JCML.combined) <- "Cluster"
table(Idents(JCML.combined))

saveRDS(JCML.combined,file="F:/scRNA/JCML/analysis3/JCML_combined_20_2
```

```
_celltype.RDS")
JCML.combined <-
readRDS(file="F:/scRNA/JCML/analysis3/JCML_combined_20_2_celltype.RDS")

#去除Doublets，高表达核糖体基因的未知细胞
JCML.combined <-
subset(JCML.combined,idents=c("Unknown","Doublets"),invert=TRUE)
JCML.combined
saveRDS(JCML.combined,file="F:/scRNA/JCML/analysis3/JCML_combined_20_2
_celltype_D.RDS")
JCML.combined <-
readRDS(file="F:/scRNA/JCML/analysis3/JCML_combined_20_2_celltype_D.RD
S")
table(Idents(JCML.combined))

#亚群信息返回后最终使用
JCML.combined <-
readRDS(file="F:/scRNA/JCML/analysis3/JCML_combined_celltype.rds")
Idents(JCML.combined) <- "Cluster"
table(Idents(JCML.combined))

# Visualization
p3 <- DimPlot(JCML.combined, reduction = "tsne", group.by =
"orig.ident")+theme(panel.background =
element_blank(),panel.grid.major = element_blank(),panel.border =
element_rect(colour="black",fill=NA))
p4 <- DimPlot(JCML.combined, reduction = "tsne",repel =
TRUE,pt.size=1)+theme(panel.background =
element_blank(),panel.grid.major = element_blank(),panel.border =
element_rect(colour="black",fill=NA))
p3 + p4

DefaultAssay(JCML.combined) <- "RNA"

#The DotPlot() function with the split.by parameter can be useful for viewing conserved cell type markers across conditions, showing both the expression level and the percentage of cells in a cluster expressing any given gene. Here we plot 2-3 strong marker genes for each of our 14 clusters.

markers.to.plot <- c("EPCAM","KRT19","KRT18",
                     "CD3D","CD3E","CD2",
                     "MS4A1","CD79A","MZB1",
                     "CD14", "CD68","TYROBP",
```

```
                     "DCN","COL1A1","THY1",
                     "RAMP2","CD34","CDH5")

DotPlot(JCML.combined, features = markers.to.plot, dot.scale = 8) +
  theme(panel.background    =    element_blank(),panel.grid.major    =
element_blank(),panel.border                                         =
element_rect(colour="black",fill=NA))+coord_flip()+
  RotatedAxis()

markers.to.plot <- c("EPCAM","KRT19",
                     "CD3D","CD3E",
                     "CD79A","MZB1",
                     "LYZ","TYROBP",
                     "DCN","COL1A1",
                     "RAMP2","CDH5")

VlnPlot(JCML.combined, features = markers.to.plot,ncol=4,pt.size = 0)
VlnPlot(JCML.combined, features = "CTLA4",pt.size = 0)

#cell component

#proportion
#orig.ident
JCML.combined                                                       <-
readRDS(file="F:/scRNA/JCML/analysis3/JCML_combined_celltype.rds")
Idents(JCML.combined) <- "Cluster"
table(Idents(JCML.combined))
JCML.combined <- subset(JCML.combined,idents="Cancer_cell",invert=TRUE)

table(JCML.combined$orig.ident)
table(Idents(JCML.combined))
prop.table(table(Idents(JCML.combined)))
table(Idents(JCML.combined), JCML.combined$orig.ident)
prop.table(table(Idents(JCML.combined),      JCML.combined$orig.ident),
margin = 2)
JCML.combined_P<-as.data.frame(prop.table(table(Idents(JCML.combined),
JCML.combined@meta.data[,"orig.ident"]), margin = 2))

ggplot(JCML.combined_P,aes(x=JCML.combined_P[,3],y=JCML.combined_P[,2]
,fill=JCML.combined_P[,1]))+
  geom_bar(position = 'stack',stat="identity")+
  labs(x="Cell proportion",y="orig.ident")+
  theme(panel.background=element_rect(fill='transparent'),panel.border
=element_rect(fill=NA,color='black'),
```

```
        legend.key=element_rect(fill='transparent',
color='transparent'),axis.text = element_text(color="black"))+
  scale_x_continuous(expand=c(0.001,0.001))+
  guides(fill = guide_legend(keywidth = 1, keyheight = 1,ncol=1,title =
'Cell types'))



library(infercnv)
library(Seurat)
library(tidyverse)
library(RColorBrewer)
rm(list = ls())


JCML.combined                                                          <-
readRDS(file="F:/scRNA/JCML/analysis3/JCML_combined_celltype.rds")
table(Idents(JCML.combined))
Idents(JCML.combined) <- "Cluster"
table(Idents(JCML.combined))
table(JCML.combined@meta.data$Cluster)

#Treat
Treat <- subset(JCML.combined,subset = orig.ident == "Treat")
Treat
table(Treat@meta.data$Cluster)

cellAnnota <- subset(Treat@meta.data, select='Cluster')
DefaultAssay(Treat) <- "RNA"
exprMatrix <- as.matrix(GetAssayData(Treat, slot='counts'))
#创建 inferCNV 对象
infercnv_obj = CreateInfercnvObject(delim = '\t',
                                    raw_counts_matrix = exprMatrix,
                                    annotations_file = cellAnnota,
                                    gene_order_file = "E:/single cell
sequence/GCTB/20201126/gencode_v21_gen_pos_complete_delete.txt",
                                    ref_group_names
=c("T_cell","B_cell","Myeloid_cell"))

setwd("F:/scRNA/JCML/analysis3/20 2/celltype/inferCNV/")
#10x 数据 cutoff 推荐使用 0.1
infercnv_obj = infercnv::run(infercnv_obj,
                             cutoff=0.1,
                             out_dir='inferCNV_Treat',
```

```
                              cluster_by_groups=TRUE,
                              denoise=TRUE,
                              HMM=TRUE)
#Naive
Naive <- subset(JCML.combined,subset = orig.ident == "Naive")
Naive
table(Naive@meta.data$Cluster)

cellAnnota <- subset(Naive@meta.data, select='Cluster')
DefaultAssay(Naive) <- "RNA"
exprMatrix <- as.matrix(GetAssayData(Naive, slot='counts'))
#创建 inferCNV 对象
infercnv_obj = CreateInfercnvObject(delim = '\t',
                                     raw_counts_matrix = exprMatrix,
                                     annotations_file = cellAnnota,
                                     gene_order_file = "E:/single cell
sequence/GCTB/20201126/gencode_v21_gen_pos_complete_delete.txt",
                                     ref_group_names
=c("T_cell","B_cell","Myeloid_cell"))

setwd("F:/scRNA/JCML/analysis3/20 2/celltype/inferCNV/")
#10x 数据 cutoff 推荐使用 0.1
infercnv_obj = infercnv::run(infercnv_obj,
                              cutoff=0.1,
                              out_dir='inferCNV_Naive',
                              cluster_by_groups=TRUE,
                              denoise=TRUE,
                              HMM=TRUE)
```
