# Supplementary material for: Single-cell analysis reveals cellular reprogramming in advanced colon cancer following FOLFOX-bevacizumab treatment
Source: Front Oncol. 2023 Jul 28;13:1219642. doi: 10.3389/fonc.2023.1219642 (PMC10421721; doi:10.3389/fonc.2023.1219642)
Supplement: Supplementary file 3 [file DataSheet_1.zip › PDF/Figure2.pdf]

```
#Cancer cell
library(ggplot2)
library(cowplot)
library(Seurat)
library(dplyr)
library(patchwork)
library(ggpubr)
library(reshape2)
theme_set(theme_cowplot())
rm(list=ls())


JCML.combined <-
readRDS(file="F:/scRNA/JCML/analysis3/JCML_combined_20_2_celltype_D.RD
S")

tumor <- subset(JCML.combined,idents = "Cancer_cell")

tumor <- RunPCA(tumor)
ElbowPlot(tumor)

tumor <- RunUMAP(tumor, reduction = "pca", dims = 1:15)
tumor <- RunTSNE(tumor, reduction = "pca", dims = 1:15)
tumor <- FindNeighbors(tumor, reduction = "pca", dims = 1:15)
saveRDS(tumor,file = "F:/scRNA/JCML/analysis3/20
2/celltype/cancer_cell/Cancer_cell_15.RDS")
tumor <- readRDS(file = "F:/scRNA/JCML/analysis3/20
2/celltype/cancer_cell/Cancer_cell_15.RDS")

tumor <- FindClusters(tumor, resolution = 0.2)
saveRDS(tumor,file = "F:/scRNA/JCML/analysis3/20
2/celltype/cancer_cell/Cancer_cell_15_0.2.RDS")
tumor <- readRDS(file = "F:/scRNA/JCML/analysis3/20
2/celltype/cancer_cell/Cancer_cell_15_0.2.RDS")

DimPlot(tumor, reduction = "umap",label = TRUE)+theme(panel.background
= element_blank(),panel.grid.major = element_blank(),panel.border =
element_rect(colour="black",fill=NA))

# Visualization
p1 <- DimPlot(tumor, reduction = "umap", group.by =
"orig.ident")+theme(panel.background =
element_blank(),panel.grid.major = element_blank(),panel.border =
element_rect(colour="black",fill=NA))
```

```
p2 <- DimPlot(tumor, reduction = "umap",label = TRUE, repel = TRUE)+theme(panel.background = element_blank(),panel.grid.major = element_blank(),panel.border = element_rect(colour="black",fill=NA))
p1 + p2

p3 <- DimPlot(tumor, reduction = "tsne", group.by = "orig.ident")+theme(panel.background = element_blank(),panel.grid.major = element_blank(),panel.border = element_rect(colour="black",fill=NA))
p4 <- DimPlot(tumor, reduction = "tsne",label = TRUE,repel = TRUE,pt.size=1)+theme(panel.background = element_blank(),panel.grid.major = element_blank(),panel.border = element_rect(colour="black",fill=NA))
p3 + p4

VlnPlot(tumor, features = c("nFeature_RNA", "nCount_RNA", "percent.mt", "percent.rb"),split.by = "orig.ident", ncol = 2)
VlnPlot(tumor, features = c("nFeature_RNA", "nCount_RNA", "percent.mt", "percent.rb"), ncol = 2)

#To visualize the two conditions side-by-side, we can use the split.by argument to show each condition colored by cluster.

DimPlot(tumor, reduction = "umap",label = FALSE, split.by = "orig.ident",ncol=6)+theme(panel.background = element_blank(),panel.grid.major = element_blank(),panel.border = element_rect(colour="black",fill=NA))
DimPlot(tumor, reduction = "tsne", split.by = "orig.ident")+theme(panel.background = element_blank(),panel.grid.major = element_blank(),panel.border = element_rect(colour="black",fill=NA))


#15 0.2
#细胞类型注释
tumor <- readRDS(file = "F:/scRNA/JCML/analysis3/202/celltype/cancer_cell/Cancer_cell_15_0.2.RDS")
table(Idents(tumor))

Sensitive=c(0,1,5)
Non_sensitive=c(2,3,4,6)

current.cluster.ids <- c(Sensitive,Non_sensitive)
new.cluster.ids <- c(rep("Sensitive",length(Sensitive)),
```

```
                rep("Non_sensitive",length(Non_sensitive)))

tumor@meta.data$Celltype <- plyr::mapvalues(x = as.integer(as.character(tumor@meta.data$seurat_clusters)), from = current.cluster.ids, to = new.cluster.ids)

table(tumor@meta.data$Celltype)

tumor$Celltype <- factor(tumor$Celltype ,level=c("Sensitive","Non_sensitive"))
Idents(tumor)<-"Celltype"
table(Idents(tumor))

saveRDS(tumor,file="F:/scRNA/JCML/analysis3/20
2/celltype/cancer_cell/Cancer_cell_15_0.2_celltype.RDS")
tumor <- readRDS(file="F:/scRNA/JCML/analysis3/20
2/celltype/cancer_cell/Cancer_cell_15_0.2_celltype.RDS")
View(tumor@meta.data)
DimPlot(tumor, reduction = "umap",label = FALSE, split.by = "orig.ident",ncol=6)+theme(panel.background = element_blank(),panel.grid.major = element_blank(),panel.border = element_rect(colour="black",fill=NA))


DefaultAssay(tumor) <- "RNA"

Sensitive_vs_NonSensitive <- FindMarkers(tumor,ident.1 = "Sensitive", ident.2 = "Non_sensitive",min.pct = 0.25)
write.csv(Sensitive_vs_NonSensitive,file = "F:/scRNA/JCML/analysis3/20
2/celltype/cancer_cell/15
0.2/celltype/Tumor_Sensitive_vs_NonSensitive_marker.csv")

VlnPlot(tumor, features = "CTLA4",pt.size = 0,group.by = "orig.ident")
VlnPlot(tumor, features = "CTLA4",pt.size = 0,split.by = "orig.ident")


#VEGF
FeaturePlot(tumor, features = c("VEGFA","VEGFB","VEGFC"))

VlnPlot(tumor, features = c("VEGFA","VEGFB","VEGFC"),pt.size = 0)
VlnPlot(tumor, features = c("VEGFA","VEGFB","VEGFC"),pt.size = 0,split.by = "orig.ident")
VlnPlot(tumor, features = c("VEGFA","VEGFB","VEGFC"),pt.size = 0,group.by = "orig.ident")
```

```r
#治疗前后加p值
#1
VlnPlot(tumor, features = c("VEGFA"),pt.size = 0,split.by = "orig.ident")+
  stat_compare_means(aes(split="orig.ident"), label = "p.signif",method="t.test")
VlnPlot(tumor, features = c("VEGFB"),pt.size = 0,split.by = "orig.ident")+
  stat_compare_means(aes(split="orig.ident"), label = "p.signif",method="t.test")

#2：#1和#2结果一致
tumor_s <- subset(tumor,idents="Sensitive")
VlnPlot(tumor_s, features = c("VEGFA"),pt.size = 0,group.by = "orig.ident")+
  stat_compare_means(label = "p.signif",method="t.test")
VlnPlot(tumor_s, features = c("VEGFB"),pt.size = 0,group.by = "orig.ident")+
  stat_compare_means(label = "p.signif",method="t.test")

tumor_ns <- subset(tumor,idents="Non_sensitive")
VlnPlot(tumor_ns, features = c("VEGFA"),pt.size = 0,group.by = "orig.ident")+
  stat_compare_means(label = "p.signif",method="t.test")
VlnPlot(tumor_ns, features = c("VEGFB"),pt.size = 0,group.by = "orig.ident")+
  stat_compare_means(label = "p.signif",method="t.test")

#治疗前，两组间加p值
tumor_naive <- subset(tumor,subset=orig.ident=="Naive")
VlnPlot(tumor_naive, features = c("VEGFA"),pt.size = 0)+
  stat_compare_means(label = "p.signif",method="t.test")
VlnPlot(tumor_naive, features = c("VEGFB"),pt.size = 0)+
  stat_compare_means(label = "p.signif",method="t.test")
```

#细胞周期分析

#Seurat包CellCycleScoring注释cell cycle，较scran包cyclone函数最主要的区别是直接根据每个cycle，一组marker基因表达值判断。

#Seurat 包提供的人的细胞中分别与 S 期、G2M 期直接相关的 marker 基因，CellCycleScoring即根据此，对每个细胞的S期、G2M期可能性进行打分；

#根据每个细胞的S期(或者G2/M期)基因集是否显著高表达，对应的score就是表示在该细胞中，S期(或者G2/M期)基因集高表达的程度(如果是负数，就认为不

属于该 phase)
#Seurat 包仅提供了人类细胞有关的 cell cycle related gene，没有小鼠的，可以将对应人的 cc.gene 转换为鼠对应的基因名，当做后者的 cell cycle related gene（因为鼠和人类基因的高度相似性)。提到的 solution 就是采用 biomaRt 包转换一下

```
library(Seurat)
str(cc.genes)

tumor <- readRDS(file = "F:/scRNA/JCML/analysis3/202/celltype/cancer_cell/Cancer_cell_15_0.2.RDS")
DefaultAssay(tumor) <- "RNA"
```

#set.ident 参数是将细胞周期的推断结果赋值为每个细胞的身份，之前聚类之后每个细胞的身份是 seurat_clusters 这一步之后 test.seu@meta.data 数据框会多 4 列：S.Score、G2M.Score、Phase、old.ident

```
tumor <- CellCycleScoring(tumor,
                          s.features = cc.genes$s.genes,
                          g2m.features = cc.genes$g2m.genes,
                          seed = 1,
                          set.ident =TRUE)


head(x = tumor@meta.data)
```

#细胞的 S.Score 与 G2M.Score 均小于 0 时，则为 G1 期；否则那个值大，就是属于哪个 phase。
#展示 1

```
plot(tumor$S.Score,tumor$G2M.Score,
     col=factor(tumor$Phase),
     main="CellCycleScoring")
legend("topright",inset=.05,
       title = "cell cycle",
       c("G1","S","G2M"), pch = c(1),col=c("black","green","red"))
```

#展示 2

```
tumor@meta.data %>% ggplot(aes(S.Score,G2M.Score))+geom_point(aes(color=Phase))+theme_minimal()


RidgePlot(tumor,features = c("PCNA","TOP2A","MKI67","MCM6"))
```

```
DefaultAssay(tumor) <- "integrated"

saveRDS(tumor,file="F:/scRNA/JCML/analysis3/20
2/celltype/cancer_cell/Cancer_cell_15_0.2_cellcycle.RDS")

DimPlot(tumor, reduction = "umap",group.by = "Phase")+
  theme(panel.background = element_blank(),panel.grid.major =
element_blank(),panel.border = element_rect(colour="black",fill=NA))

DimPlot(tumor, reduction = "umap",group.by = "Phase",split.by =
"orig.ident")+
  theme(panel.background = element_blank(),panel.grid.major =
element_blank(),panel.border = element_rect(colour="black",fill=NA))

DimPlot(tumor, reduction = "umap",
        group.by = "Phase",
        shape.by = "orig.ident",
        pt.size =2)

DimPlot(tumor, reduction = "pca",
        group.by = "orig.ident",
        shape.by = "Phase",
        pt.size =2)

DimPlot(tumor, reduction = "tsne",
        group.by = "orig.ident",
        shape.by = "Phase")

DimPlot(tumor, reduction = "umap",
        group.by = "orig.ident",
        shape.by = "Phase",
        pt.size =2)

#cell component
#proportion
tumor <- readRDS(file = "F:/scRNA/JCML/analysis3/20
2/celltype/cancer_cell/Cancer_cell_15_0.2_cellcycle.RDS")
#orig.ident
table(tumor$orig.ident)
table(Idents(tumor))
prop.table(table(Idents(tumor)))
table(Idents(tumor), tumor$orig.ident)
prop.table(table(Idents(tumor), tumor$orig.ident), margin = 2)
tumor_p<-as.data.frame(prop.table(table(Idents(tumor),
```

```
tumor@meta.data[,"orig.ident"]), margin = 2))
#纵向
ggplot(tumor_p,aes(x=tumor_p[,2],y=tumor_p[,3],fill=tumor_p[,1]))+
  geom_bar(position = 'stack',stat="identity")+
  labs(x="orig.ident",y="Cell proportion")+
  theme(panel.background=element_rect(fill='transparent',
color='black'),panel.border =element_rect(fill=NA,color='black'),
        legend.key=element_rect(fill='transparent',
color='transparent'),axis.text = element_text(color="black"))+
  scale_y_continuous(expand=c(0.001,0.001))+
  guides(fill = guide_legend(keywidth = 1, keyheight = 1,ncol=1,title =
'Cell types'))



#GSEA 分析
library(presto)
library(msigdbr)
library(fgsea)
library(dplyr)
library(ggplot2)
library(tibble)

#按治疗的敏感性分群
scRNAsub <- readRDS(file = "F:/scRNA/JCML/analysis3/202/celltype/cancer_cell/Cancer_cell_15_0.2_celltype.RDS")

table(Idents(scRNAsub))
DefaultAssay(scRNAsub) <- "RNA"
#查找 marker 基因
scRNAsub.markers <- FindAllMarkers(scRNAsub, only.pos = TRUE, min.pct =
0.25, logfc.threshold = 0.25)

#使用 wilcoxauc()计算每个 cluster 的差异基因:scRNAsub.genes <-
wilcoxauc(scRNAsub, 'seurat_clusters')
scRNAsub.genes <- wilcoxauc(scRNAsub, 'Celltype')
head(scRNAsub.genes)
# 查看每个 cluster 中有多少基因（与矩阵的基因数一致）
dplyr::count(scRNAsub.genes, group)
#选取 group Sensitive 的差异基因进行 GSEA 分析，将 Sensitive 的 差异基因整理为 GSEA 分析需要的数据格式（也可输入注释好的细胞群的差异基因）
scRNAsub.genes %>%
  dplyr::filter(group == "Sensitive") %>%
  arrange(desc(logFC), desc(auc)) %>%
```

```
  head(n = 10)      #进行降序排序
# 仅选择 fgsea 的 feature 和 auc 列
Sensitive.genes<- scRNAsub.genes %>% dplyr::filter(group ==
"Sensitive") %>% arrange(desc(auc)) %>% dplyr::select(feature, auc)
ranks<- deframe(Sensitive.genes)
head(ranks)


#选择自己数据的物种以及要做的 GSEA 的数据库类型，准备目标基因集的输入文
件
##查看物种的数据
msigdbr_species()
#我们使用 50 个 hallmark 基因集
m_df<- msigdbr(species = "Homo sapiens", category = "H")

head(m_df)

##将 m_df 的基因与通路取出并改成一个通路对应相应基因的格式
fgsea_sets<- m_df %>% split(x = .$gene_symbol, f = .$gs_name)
#fgsea_sets$GSE11057_NAIVE_VS_MEMORY_CD4_TCELL_UP
#以 gs_name 为 factor 对 gene_symbol 进行分类，统计落在每个 gs_name 中的
gene_symbol 的个数，并生成 list。
summary(fgsea_sets)

#使用 fgsea 进行基因集富集并绘图
#富集分析
fgseaRes<- fgsea(fgsea_sets, stats = ranks, nperm = 1000)
#nperm 设置的是 permutation 次数
#整理数据：
fgseaResTidy <- fgseaRes %>%  as_tibble() %>% arrange(desc(NES))
fgseaResTidy %>% dplyr::select(-leadingEdge, -ES, -nMoreExtreme) %>%
arrange(padj) %>% head()
View(fgseaResTidy)

#绘图:
#应用标准化富集分数绘制 barplot:显示 top20 信号通路
ggplot(fgseaResTidy %>% filter(pval < 0.05) %>% head(n= 50),
aes(reorder(pathway, NES), NES)) +
  geom_col(aes(fill= NES < 0)) +
  coord_flip() +
  labs(x="Pathway", y="Normalized Enrichment Score",
       title="Hallmark pathways NES from GSEA") +
  theme_minimal() ####以 7.5 进行绘图填色
ggplot(fgseaResTidy %>% filter(padj < 0.01) %>% head(n= 50),
```

```
aes(reorder(pathway, NES), NES)) +
  geom_col(aes(fill= NES < 0)) +
  coord_flip() +
  labs(x="Pathway", y="Normalized Enrichment Score",
       title="Hallmark pathways NES from GSEA") +
  theme_minimal() ####以 7.5 进行绘图填色

ggplot(fgseaResTidy  %>%  filter(pval  <  0.05)  %>%  head(n=  38),
aes(reorder(pathway, NES), NES)) +
  geom_col(aes(fill= NES < 0)) +
  coord_flip() +
  labs(x="Pathway", y="Normalized Enrichment Score",
       title="Hallmark pathways NES from GSEA") +
  theme_minimal() ####以 7.5 进行绘图填色

ggplot(fgseaResTidy  %>%  filter(pval  <  0.05)  %>%  head(n=  50),
aes(reorder(pathway, NES), -log10(pval))) +
  geom_col(aes(fill= NES < 0)) +
  coord_flip() +
  labs(x="Pathway", y="-log10(pval)",
       title="Hallmark pathways NES from GSEA") +
  theme_minimal() ####以 7.5 进行绘图填色


#输出数据，以选择想要展示的通路

#按治疗的敏感性分群
Sensitive <- fgseaResTidy %>% filter(pval < 0.01)
View(Sensitive)
pathway <- Sensitive$pathway
pval<-Sensitive$pval
padj<-Sensitive$padj
ES <-Sensitive$ES
NES<-Sensitive$NES
LeadingEdge<-Sensitive$leadingEdge
size<-Sensitive$size

df<-data.frame(pathway,pval,padj,ES,NES,size)
#2
write.csv(df,file = "F:/scRNA/JCML/analysis3/202/celltype/cancer_cell/15
0.2/celltype/GSEA/Sensitive_VS_NonSensitive_p0.01.csv",row.names =
TRUE)
```

```
library(ggplot2)
#UP used
UP              <-             read.csv(file="F:/scRNA/JCML/analysis3/20
2/celltype/cancer_cell/15
0.2/celltype/GSEA/Sensitive_VS_NonSensitive_p0.01_UP_used.csv")
ggplot(data = UP) +
  geom_bar(aes(-log10(pval),reorder(pathway,  -log10(pval)),  ),stat  =
"identity",fill="red") +
  theme(panel.background = element_blank(),
        panel.grid.major = element_blank(),
        panel.border = element_rect(colour="black",fill=NA))+
  labs(y="Pathway", x="-Log10(pval)",
       title="Upregulated in Sensitive")

#DOWN used
DOWN            <-             read.csv(file="F:/scRNA/JCML/analysis3/20
2/celltype/cancer_cell/15
0.2/celltype/GSEA/Sensitive_VS_NonSensitive_p0.01_DOWN_used.csv")
ggplot(data = DOWN) +
  geom_bar(aes(-log10(pval),reorder(pathway,  -log10(pval)),  ),stat  =
"identity",fill="blue") +
  theme(panel.background = element_blank(),
        panel.grid.major = element_blank(),
        panel.border = element_rect(colour="black",fill=NA))+
  labs(y="Pathway", x="-Log10(pval)",
       title="Upregulated in Non_sensitive")

#GSEA 图
plotEnrichment(fgsea_sets[["GSE9650_EXHAUSTED_VS_MEMORY_CD8_TCELL_UP"]
],
               ranks)                                                  +
labs(title="GSE9650_EXHAUSTED_VS_MEMORY_CD8_TCELL_UP")
plotEnrichment(fgsea_sets[["GSE9650_EXHAUSTED_VS_MEMORY_CD8_TCELL_DN"]
],
               ranks)                                                  +
labs(title="GSE9650_EXHAUSTED_VS_MEMORY_CD8_TCELL_DN")



#代谢及其他特征 评分 score
library(Seurat)
?AddModuleScore
library(tidyverse)
library(Matrix)
```

```
library(cowplot)
library(readxl)

## 输入数据：Seurat 对象和一个 gene list。

tumor <- readRDS(file="F:/scRNA/JCML/analysis3/202/celltype/cancer_cell/Cancer_cell_15_0.2.RDS")

scRNAsub <- tumor


DefaultAssay(scRNAsub) <- "RNA"
table(Idents(scRNAsub))

tumor <- scRNAsub
DefaultAssay(tumor) <- "RNA"

#一.代谢、能量

#1.Glycolysis
#
Glycolysis_G <- readxl::read_xlsx("E:/single cell sequence/Score gene sets/GOBP_POSITIVE_REGULATION_OF_GLYCOLYTIC_PROCESS.xlsx",col_names = FALSE)
#View(Glycolysis_G)
#转换成 list
gene <- as.list(Glycolysis_G)
scRNAsub <- AddModuleScore(
  object = scRNAsub,
  features = gene,
  ctrl =100,
  name = 'Glycolysis_G',
  seed=1)

#
Fatty_Acid_Metabolism_K <- readxl::read_xlsx("E:/single cell sequence/Score gene sets/KEGG_FATTY_ACID_METABOLISM.xlsx",col_names = FALSE)
#转换成 list
gene <- as.list(Fatty_Acid_Metabolism_K)
scRNAsub <- AddModuleScore(
  object = scRNAsub,
  features = gene,
  ctrl =100,
```

```
  name = 'Fatty_Acid_Metabolism_K',
  seed=1)

#
Oxidative_Phosphorylation_K    <-    readxl::read_xlsx("E:/single    cell
sequence/Score                                                     gene
sets/KEGG_OXIDATIVE_PHOSPHORYLATION.xlsx",col_names = FALSE)
#View(Oxidative_Phosphorylation)
#转换成 list
gene <- as.list(Oxidative_Phosphorylation_K)

scRNAsub <- AddModuleScore(
  object = scRNAsub,
  features = gene,
  ctrl =100,
  name = 'Oxidative_Phosphorylation_K',
  seed=1)

###计算结果保存在 scRNAsub@meta.data[["CD_Features1"]]
###得到的 score 是在每个细胞中算出来的我们感兴趣的基因的表达均值。
###背景基因的平均值在于找每个基因所在的 bin，在该 bin 内随机抽取相应的
ctrl 个基因作为背景，
###最后所有的目标基因算一个平均值，所有的背景基因算一个平均值，两者想减
就是该 gene set 的 score 值。
colnames(scRNAsub@meta.data)

colnames(scRNAsub@meta.data)[10] <- 'Glycolysis_G'
colnames(scRNAsub@meta.data)[11] <- 'Fatty_Acid_Metabolism_K'
colnames(scRNAsub@meta.data)[12] <- 'Oxidative_Phosphorylation_K'

colnames(scRNAsub@meta.data)

library(ggpubr)
library(reshape2)
data1<- FetchData(scRNAsub,vars = c("orig.ident","seurat_clusters",

"Glycolysis_G","Fatty_Acid_Metabolism_K","Oxidative_Phosphorylation_K"
))

#Tumor1:ctrl = 100,
write.csv(data1,file="F:/scRNA/JCML/analysis3/20
2/celltype/cancer_cell/15 0.2/score/Tumor1.csv")

data1<-                      read.csv(file="F:/scRNA/JCML/analysis3/20
```

```
2/celltype/cancer_cell/15 0.2/score/Tumor1.csv",header = TRUE)


#按治疗与否
#
ggviolin(data1, x = "orig.ident", y = "Glycolysis_G",
         fill = "orig.ident", add = "boxplot",
         ylab = "Glycolysis_G", xlab = "orig.ident")+
  stat_compare_means(label ="p.signif",method="t.test",hide.ns = FALSE)

#
ggviolin(data1, x = "orig.ident", y = "Fatty_Acid_Metabolism_K",
         fill = "orig.ident", add = "boxplot",
         ylab = "Fatty_Acid_Metabolism_K", xlab = "orig.ident")+
  stat_compare_means(label ="p.signif",method="t.test",hide.ns = FALSE)

ggviolin(data1, x = "orig.ident", y = "Oxidative_Phosphorylation_K",
         fill = "orig.ident", add = "boxplot",
         ylab = "Oxidative_Phosphorylation_K", xlab = "orig.ident")+
  stat_compare_means(label ="p.signif",method="t.test",hide.ns = FALSE)

#按治疗与否
#
ggviolin(data1, x = "orig.ident", y = "Glycolysis_G",
         fill = "orig.ident", add = "boxplot",
         ylab = "Glycolysis_G", xlab = "orig.ident")+
  stat_compare_means(label ="p.signif",method="wilcox.test",hide.ns =
FALSE)

#
ggviolin(data1, x = "orig.ident", y = "Fatty_Acid_Metabolism_K",
         fill = "orig.ident", add = "boxplot",
         ylab = "Fatty_Acid_Metabolism_K", xlab = "orig.ident")+
  stat_compare_means(label ="p.signif",method="wilcox.test",hide.ns =
FALSE)

ggviolin(data1, x = "orig.ident", y = "Oxidative_Phosphorylation_K",
         fill = "orig.ident", add = "boxplot",
         ylab = "Oxidative_Phosphorylation_K", xlab = "orig.ident")+
  stat_compare_means(label ="p.signif",method="wilcox.test",hide.ns =
FALSE)
```
