# Supplementary material for: Single-cell analysis reveals cellular reprogramming in advanced colon cancer following FOLFOX-bevacizumab treatment
Source: Front Oncol. 2023 Jul 28;13:1219642. doi: 10.3389/fonc.2023.1219642 (PMC10421721; doi:10.3389/fonc.2023.1219642)
Supplement: Supplementary file 3 [file DataSheet_1.zip › PDF/Figure3.pdf]

```
library(ggplot2)
library(cowplot)
library(Seurat)
library(dplyr)
library(patchwork)
library(ggpubr)
library(reshape2)

rm(list=ls())

setwd("F:/scRNA/JCML/analysis3/20 2/celltype/Myeloid/")
JCML.combined <-
readRDS(file="F:/scRNA/JCML/analysis3/JCML_combined_20_2_celltype_D.RDS")

Myeloid_cell <- subset(JCML.combined,idents="Myeloid_cell")
DefaultAssay(Myeloid_cell) <- "integrated"
Myeloid_cell <- RunPCA(Myeloid_cell,verbose = FALSE)
ElbowPlot(Myeloid_cell)

Myeloid_cell <- RunUMAP(Myeloid_cell, reduction = "pca", dims = 1:15)
Myeloid_cell <- RunTSNE(Myeloid_cell, reduction = "pca", dims = 1:15)
Myeloid_cell <- FindNeighbors(Myeloid_cell, reduction = "pca", dims = 1:15)
saveRDS(Myeloid_cell,file = "F:/scRNA/JCML/analysis3/20 2/celltype/Myeloid/Myeloid_cell_15.RDS")
Myeloid_cell <- readRDS(file = "F:/scRNA/JCML/analysis3/20 2/celltype/Myeloid/Myeloid_cell_15.RDS")

Myeloid_cell <- FindClusters(Myeloid_cell, resolution = 0.1)
saveRDS(Myeloid_cell,file = "F:/scRNA/JCML/analysis3/20 2/celltype/Myeloid/Myeloid_cell_15_0.1.RDS")
Myeloid_cell <- readRDS(file = "F:/scRNA/JCML/analysis3/20 2/celltype/Myeloid/Myeloid_cell_15_0.1.RDS")


p3 <- DimPlot(Myeloid_cell, reduction = "tsne", group.by = "orig.ident")+theme(panel.background = element_blank(),panel.grid.major = element_blank(),panel.border = element_rect(colour="black",fill=NA))
p4 <- DimPlot(Myeloid_cell, reduction = "tsne",label = TRUE, repel = TRUE,pt.size=1)+theme(panel.background = element_blank(),panel.grid.major = element_blank(),panel.border = element_rect(colour="black",fill=NA))
```

```
p4 + p3

p3 <- DimPlot(Myeloid_cell, reduction = "umap", group.by = "orig.ident")+theme(panel.background = element_blank(),panel.grid.major = element_blank(),panel.border = element_rect(colour="black",fill=NA))
p4 <- DimPlot(Myeloid_cell, reduction = "umap",label = TRUE, repel = TRUE,pt.size=1)+theme(panel.background = element_blank(),panel.grid.major = element_blank(),panel.border = element_rect(colour="black",fill=NA))
p4 + p3

table(Idents(Myeloid_cell))

DefaultAssay(Myeloid_cell) <- "RNA"

Myeloid_cell.markers <- FindAllMarkers(Myeloid_cell, only.pos = TRUE, min.pct = 0.25, logfc.threshold =0.25)

top10 <- Myeloid_cell.markers %>% group_by(cluster) %>% top_n(n = 10, wt = avg_log2FC)
DefaultAssay(Myeloid_cell) <- "integrated"
DoHeatmap(Myeloid_cell, features = top10$gene)


#VEGF
VlnPlot(Myeloid_cell, features = c("VEGFA","VEGFB","VEGFC"),pt.size = 0)
VlnPlot(Myeloid_cell, features = c("VEGFA","VEGFB","VEGFC"),pt.size = 0,split.by = "orig.ident")
VlnPlot(Myeloid_cell, features = c("VEGFA","VEGFB","VEGFC"),pt.size = 0,group.by = "orig.ident")


#
VlnPlot(Myeloid_cell, features = c("SPP1"," C1QC"),pt.size = 0)




#DC:"CD1A","CD1C","FCER1A","CLEC9A","CCR7"
#pDC: IGJ,"LILRA4", "CLEC4C/DLEC/BDCA2",IL3RA/CD123,NRP1/BDCA4
#Langerhans cell:FCER1A
#migratory DCs:CCR7,AXL
```

```
#CD83 is a specific marker of mature DCs;CR2/CD21,CLU/clusterin for follicular DCs
#classical DC1 marker gene THBD(CD141)
#DC2 cells (CD1C and CLEC10A),
#DC3 cells (CCL19,LAMP3, and CCR7) could represent a Myeloid_cell-specific DC subpopulation, because they were also detected in our reanalysis of melanoma , head andneck head cancer,but were not found in PBMCs
VlnPlot(Myeloid_cell, features = c("CD1A","CD1C","FCER1A","CLEC9A","CCR7",

"IGJ","LILRA4","CLEC4C","DLEC","BDCA2","IL3RA","NRP1",

"AXL","THBD","CLEC10A","CD83","CR2","CLU","CCL19","LAMP3","IDO1"))
VlnPlot(Myeloid_cell, features = c("IFNB1"))


#neutrophils characterized by S100A8, S100A9, and G0S2
VlnPlot(Myeloid_cell, features = c("CSF3R","S100A8", "S100A9","G0S2","MPO","CD15","FUT4","CD32","CD66b","CEACAM8","SELL","ELANE","BPI"))

#MAST CELL
VlnPlot(Myeloid_cell, features = c("KIT","MS4A2","GATA2","IL2RA","PTPRC","FCER2","TPSAB1","FCER1A"))


# find markers for every cluster compared to all remaining cells
Myeloid_cell.markers <- FindAllMarkers(Myeloid_cell, only.pos = TRUE, min.pct = 0.25, logfc.threshold =0.25)
top10 <- Myeloid_cell.markers %>% group_by(cluster) %>% top_n(n = 10, wt = avg_log2FC)
DefaultAssay(Myeloid_cell) <- "integrated"
DoHeatmap(Myeloid_cell, features = top10$gene) + NoLegend()


VlnPlot(Myeloid_cell, features = c("nFeature_RNA", "nCount_RNA", "percent.mt", "percent.rb"),split.by = "orig.ident", ncol = 2)
VlnPlot(Myeloid_cell, features = c("nFeature_RNA", "nCount_RNA", "percent.mt", "percent.rb"), ncol = 2)

#15 0.1
#细胞类型注释 1
Myeloid_cell <- readRDS(file = "F:/scRNA/JCML/analysis3/20
```

```
2/celltype/Myeloid/Myeloid_cell_15_0.1.RDS")

TAM_M0=c(0)
TAM_M1M2=c(1)
Neutrophil=c(2)
Mast_cell=c(3)
current.cluster.ids <- c(TAM_M0,TAM_M1M2,Neutrophil,Mast_cell)
new.cluster.ids <- c(rep("TAM_M0",length(TAM_M0)),
                     rep("TAM_M1M2",length(TAM_M1M2)),
                     rep("Neutrophil",length(Neutrophil)),
                     rep("Mast_cell",length(Mast_cell)))

Myeloid_cell@meta.data$Celltype <- plyr::mapvalues(x = as.integer(as.character(Myeloid_cell@meta.data$seurat_clusters)), from = current.cluster.ids, to = new.cluster.ids)
head(Myeloid_cell@meta.data)
table(Myeloid_cell@meta.data$Celltype)
table(Idents(Myeloid_cell))

Myeloid_cell$Celltype <- factor(Myeloid_cell$Celltype ,level=c("TAM_M0","TAM_M1M2","Neutrophil","Mast_cell"))

Idents(Myeloid_cell)<-"Celltype"
table(Idents(Myeloid_cell))

saveRDS(Myeloid_cell,file="F:/scRNA/JCML/analysis3/20
2/celltype/Myeloid/15 0.1/celltype/Myeloid_15_0.1_Celltype")
Myeloid_cell <- readRDS(file = "F:/scRNA/JCML/analysis3/20
2/celltype/Myeloid/15 0.1/celltype/Myeloid_15_0.1_Celltype")

DefaultAssay(Myeloid_cell) <- "RNA"


markers.to.plot <- c("CD14", "CD68",
                     "CD163","MS4A4A","TGFB1",
                     "CD86","IL1B","TNF",
                     "S100A8","S100A9","G0S2",
                     "KIT","GATA2","TPSAB1")

VlnPlot(Myeloid_cell, features =c("CD14", "CD68",
                                  "CD163","MS4A4A","TGFB1",
                                  "CD86","IL1B","TNF",
                                  "S100A8","S100A9","G0S2",
```

```
"KIT","GATA2","TPSAB1"),ncol=4,pt.size = 0)

DotPlot(Myeloid_cell, features = markers.to.plot, dot.scale = 8) +
  theme(panel.background = element_blank(),panel.grid.major =
element_blank(),panel.border =
element_rect(colour="black",fill=NA))+coord_flip()+
  RotatedAxis()

#细胞类型注释 2
Myeloid_cell <- readRDS(file = "F:/scRNA/JCML/analysis3/20
2/celltype/Myeloid/Myeloid_cell_15_0.1.RDS")

TAM=c(0,1)
Neutrophil=c(2)
Mast_cell=c(3)
current.cluster.ids <- c(TAM,Neutrophil,Mast_cell)
new.cluster.ids <- c(rep("TAM",length(TAM)),
                     rep("Neutrophil",length(Neutrophil)),
                     rep("Mast_cell",length(Mast_cell)))

Myeloid_cell@meta.data$Celltype <- plyr::mapvalues(x =
as.integer(as.character(Myeloid_cell@meta.data$seurat_clusters)), from
= current.cluster.ids, to = new.cluster.ids)
head(Myeloid_cell@meta.data)
table(Myeloid_cell@meta.data$Celltype)
table(Idents(Myeloid_cell))

Myeloid_cell$Celltype <-
factor(Myeloid_cell$Celltype ,level=c("TAM","Neutrophil","Mast_cell"))

Idents(Myeloid_cell)<-"Celltype"
table(Idents(Myeloid_cell))

saveRDS(Myeloid_cell,file="F:/scRNA/JCML/analysis3/20
2/celltype/Myeloid/15 0.1/celltype/Myeloid_15_0.1_Celltype2")
Myeloid_cell <- readRDS(file = "F:/scRNA/JCML/analysis3/20
2/celltype/Myeloid/15 0.1/celltype/Myeloid_15_0.1_Celltype2")


p3 <- DimPlot(Myeloid_cell, reduction = "tsne", group.by =
"orig.ident")+theme(panel.background =
element_blank(),panel.grid.major = element_blank(),panel.border =
element_rect(colour="black",fill=NA))
```

```
p4 <- DimPlot(Myeloid_cell, reduction = "tsne", repel = TRUE,pt.size=1)+theme(panel.background = element_blank(),panel.grid.major = element_blank(),panel.border = element_rect(colour="black",fill=NA))
p4 + p3

p3 <- DimPlot(Myeloid_cell, reduction = "umap", group.by = "orig.ident")+theme(panel.background = element_blank(),panel.grid.major = element_blank(),panel.border = element_rect(colour="black",fill=NA))
p4 <- DimPlot(Myeloid_cell, reduction = "umap", repel = TRUE,pt.size=1)+theme(panel.background = element_blank(),panel.grid.major = element_blank(),panel.border = element_rect(colour="black",fill=NA))
p4 + p3


#cell component
#proportion
Myeloid_cell <- readRDS(file = "F:/scRNA/JCML/analysis3/202/celltype/Myeloid/15 0.1/celltype/Myeloid_15_0.1_Celltype")

table(Myeloid_cell$orig.ident)
table(Idents(Myeloid_cell))
prop.table(table(Idents(Myeloid_cell)))
table(Idents(Myeloid_cell), Myeloid_cell$orig.ident)
prop.table(table(Idents(Myeloid_cell), Myeloid_cell$orig.ident), margin = 2)
Myeloid_cell_p<-as.data.frame(prop.table(table(Idents(Myeloid_cell), Myeloid_cell@meta.data[,"orig.ident"]), margin = 2))

ggplot(Myeloid_cell_p,aes(x=Myeloid_cell_p[,2],y=Myeloid_cell_p[,3],fill=Myeloid_cell_p[,1]))+
  geom_bar(position = 'stack',stat="identity")+
  labs(x="Sample",y="Cell proportion")+

theme(panel.background=element_rect(fill='transparent',color='black'), panel.border =element_rect(fill=NA,color='black'),
        legend.key=element_rect(fill='transparent', color='transparent'),axis.text = element_text(color="black"))+
  scale_y_continuous(expand=c(0.001,0.001))+
  guides(fill = guide_legend(keywidth = 1, keyheight = 1,ncol=1,title = 'Cell types'))
```

```
#Macrophage
Myeloid_cell <- readRDS(file = "F:/scRNA/JCML/analysis3/20
2/celltype/Myeloid/15 0.1/celltype/Myeloid_15_0.1_Celltype")
table(Idents(Myeloid_cell))

macrophage <- subset(Myeloid_cell,idents=c("TAM_M0","TAM_M1M2"))

saveRDS(macrophage,file = "F:/scRNA/JCML/analysis3/20
2/celltype/Myeloid/15
0.1/celltype/macrophage/Myeloid_cell_15_0.1_celltype_macrophage.RDS")
table(Idents(macrophage))

#macrophage 评分
library(Seurat)
?AddModuleScore
library(tidyverse)
library(Matrix)
library(cowplot)
library(readxl)
## 输入数据：Seurat 对象和一个 gene list。
macrophage <- readRDS(file = "F:/scRNA/JCML/analysis3/20
2/celltype/Myeloid/15
0.1/celltype/macrophage/Myeloid_cell_15_0.1_celltype_macrophage.RDS")

table(Idents(macrophage))
DefaultAssay(macrophage) <- "RNA"

#VEGF
VlnPlot(macrophage, features = c("VEGFA","VEGFB","VEGFC"),pt.size = 0)

VlnPlot(macrophage, features = c("VEGFA","VEGFB","VEGFC"),pt.size =
0,split.by = "orig.ident")

VlnPlot(macrophage, features = c("VEGFA","VEGFB","VEGFC"),pt.size =
0,group.by = "orig.ident")

VlnPlot(macrophage, features = c("VEGFA"),pt.size = 0,group.by =
"orig.ident")+
  stat_compare_means(label ="p.signif" ,method="wilcox.test",hide.ns =
FALSE)

VlnPlot(macrophage, features = c("VEGFB"),pt.size = 0,group.by =
"orig.ident")+
```

```
  stat_compare_means(label ="p.signif" ,method="wilcox.test",hide.ns = FALSE)


#M1
M1 <- readxl::read_xlsx("E:/single cell sequence/Score gene sets/immunocyte/M1.xlsx")
#View(M1)
#转换成list
gene <- as.list(M1)
macrophage <- AddModuleScore(
  object = macrophage,
  features = gene,
  ctrl = 100,
  name = 'M1_Score')

#M2
M2 <- readxl::read_xlsx("E:/single cell sequence/Score gene sets/immunocyte/M2.xlsx")
#View(M2)
#转换成list
gene <- as.list(M2)
macrophage <- AddModuleScore(
  object = macrophage,
  features = gene,
  ctrl = 100,
  name = 'M2_Score')

#Pro_inflammatory
Pro_inflammatory <- readxl::read_xlsx("E:/single cell sequence/Score gene sets/immunocyte/Pro_inflammatory.xlsx")
#View(Pro_inflammatory)
#转换成list
gene <- as.list(Pro_inflammatory)
macrophage <- AddModuleScore(
  object = macrophage,
  features = gene,
  ctrl = 100,
  name = 'Pro_inflammatory_Score')


#Anti_inflammatory
Anti_inflammatory <- readxl::read_xlsx("E:/single cell sequence/Score gene sets/immunocyte/Anti_inflammatory.xlsx")
```

```
#View(Anti_inflammatory)
#转换成 list
gene <- as.list(Anti_inflammatory)
macrophage <- AddModuleScore(
  object = macrophage,
  features = gene,
  ctrl = 100,
  name = 'Anti_inflammatory_Score')

#KEGG_antigen_process_present_geneset
APP <- read.csv("E:/single cell sequence/Score gene sets/immunocyte/KEGG_antigen_process_present.csv")
#View(APP)
#转换成 list
gene <- as.list(APP)
macrophage <- AddModuleScore(
  object = macrophage,
  features = gene,
  ctrl = 100,
  name = 'APP_score')


#co-stimulatory:OX40L/TNFSF4,4-1BBL/TNFSF9.CD40LG/CD154,"CD27","CD40","CD70"
Costimulatory <- read.csv("E:/single cell sequence/Score gene sets/immunocyte/Costimulatory ligand used.csv")
#View(Costimulatory)
#"CD80","CD86","ICOSLG","TNFSF4","TNFSF9","TNFSF14"
#转换成 list
gene <- as.list(Costimulatory)
macrophage <- AddModuleScore(
  object = macrophage,
  features = gene,
  ctrl = 100,
  name = 'Costimulatory_score')

MHC_I <- readxl::read_xlsx("E:/single cell sequence/Score gene sets/HLA_classI.xlsx",col_names = FALSE)
#View(MHC_I)
#转换成 list
gene <- as.list(MHC_I)
macrophage <- AddModuleScore(
  object = macrophage,
  features = gene,
```

```
  ctrl =100,
  name = 'MHC_I',
  seed=1)

MHC_II  <-  readxl::read_xlsx("E:/single  cell  sequence/Score  gene sets/HLA_classII.xlsx",col_names = FALSE)
#View(APP)
#转换成 list
gene <- as.list(MHC_II)
macrophage <- AddModuleScore(
  object = macrophage,
  features = gene,
  ctrl =100,
  name = 'MHC_II',
  seed=1)

#IFNγ_response
IFNγ_response <- readxl::read_xlsx("E:/single cell sequence/Score gene sets/HALLMARK_INTERFERON_GAMMA_RESPONSE.xlsx",col_names = FALSE)
#View(IFNγ_response)
#转换成 list
gene <- as.list(IFNγ_response)
macrophage <- AddModuleScore(
  object = macrophage,
  features = gene,
  ctrl =100,
  name = 'IFNγ_response',
  seed=1)



#血管生成 HALLMARK_ANGIOGENESIS
Angiogenesis <- readxl::read_xlsx("E:/single cell sequence/Score gene sets/HALLMARK_ANGIOGENESIS.xlsx",col_names = FALSE)
#View(Angiogenesis)
#转换成 list
gene <- as.list(Angiogenesis)
macrophage <- AddModuleScore(
  object = macrophage,
  features = gene,
  ctrl =100,
  name = 'Angiogenesis',
  seed=1)
```

###计算结果保存在 macrophage@meta.data[["CD_Features1"]]
###得到的 score 是在每个细胞中算出来的我们感兴趣的基因的表达均值。
###背景基因的平均值在于找每个基因所在的 bin，在该 bin 内随机抽取相应的 ctrl 个基因作为背景，
###最后所有的目标基因算一个平均值，所有的背景基因算一个平均值，两者想减就是该 gene set 的 score 值。

```
colnames(macrophage@meta.data)
colnames(macrophage@meta.data)[11] <- 'M1_score'
colnames(macrophage@meta.data)[12] <- 'M2_score'
colnames(macrophage@meta.data)[13] <- 'Pro_inflammatory_score'
colnames(macrophage@meta.data)[14] <- 'Anti_inflammatory_score'
colnames(macrophage@meta.data)[15] <- 'APP_score'
colnames(macrophage@meta.data)[16] <- 'Costimulatory_score'
colnames(macrophage@meta.data)[17] <- 'MHC_I'
colnames(macrophage@meta.data)[18] <- 'MHC_II'
colnames(macrophage@meta.data)[19] <- 'IFNγ_response'
colnames(macrophage@meta.data)[20] <- 'Angiogenesis'


colnames(macrophage@meta.data)

library(ggpubr)
library(reshape2)
#celltype
data1<- FetchData(macrophage,vars = c("orig.ident","Celltype",

"M1_score","M2_score","Pro_inflammatory_score","Anti_inflammatory_score",

"APP_score","Costimulatory_score",
                                       "MHC_I","MHC_II",
                                       "IFNγ_response",
                                       "Angiogenesis"))
write.csv(data1,file="F:/scRNA/JCML/analysis3/20 2/celltype/Myeloid/15 0.1/celltype/macrophage/score/data1.csv")
view(data1)

data1<- read.csv(file="F:/scRNA/JCML/analysis3/20 2/celltype/Myeloid/15 0.1/celltype/macrophage/score/data1.csv",header = TRUE)
```

#按样本处理

```
#按样本处理
#wilcox
ggviolin(data1, x = "orig.ident", y = "M1_score",
         fill = "orig.ident", add = "boxplot",
         ylab = "M1_score", xlab = "sample")+
  stat_compare_means(label ="p.signif" ,method="wilcox.test",hide.ns =
FALSE)
ggviolin(data1, x = "orig.ident", y = "M2_score",
         fill = "orig.ident", add = "boxplot",
         ylab = "M2_score", xlab = "sample")+
  stat_compare_means(label ="p.signif" ,method="wilcox.test",hide.ns =
FALSE)
#Pro_inflammatory_score
ggviolin(data1, x = "orig.ident", y = "Pro_inflammatory_score",
         fill = "orig.ident", add = "boxplot",
         ylab = "Pro_inflammatory_score", xlab = "sample")+
  stat_compare_means(label ="p.signif" ,method="wilcox.test",hide.ns =
FALSE)
ggviolin(data1, x = "orig.ident", y = "Anti_inflammatory_score",
         fill = "orig.ident", add = "boxplot",
         ylab = "Anti_inflammatory_score", xlab = "sample")+
  stat_compare_means(label ="p.signif" ,method="wilcox.test",hide.ns =
FALSE)


ggviolin(data1, x = "orig.ident", y = "Angiogenesis",
         fill = "orig.ident", add = "boxplot",
         ylab = "Angiogenesis", xlab = "sample")+
  stat_compare_means(label ="p.signif" ,method="wilcox.test",hide.ns =
FALSE)
```
