# Supplementary material for: Single-cell analysis reveals cellular reprogramming in advanced colon cancer following FOLFOX-bevacizumab treatment
Source: Front Oncol. 2023 Jul 28;13:1219642. doi: 10.3389/fonc.2023.1219642 (PMC10421721; doi:10.3389/fonc.2023.1219642)
Supplement: Supplementary file 3 [file DataSheet_1.zip › PDF/Figure4 and figureS2.pdf]

```
#T_cell
library(ggplot2)
library(cowplot)
library(Seurat)
library(dplyr)
library(patchwork)
library(ggpubr)

setwd("F:/scRNA/JCML/analysis3/20 2/celltype/T_cell/")

JCML.combined <-
readRDS(file="F:/scRNA/JCML/analysis3/JCML_combined_20_2_celltype_D.RDS")
view(JCML.combined@meta.data)
T_cell <- subset(JCML.combined, idents = "T_cell")
T_cell

DefaultAssay(T_cell) <- "integrated"
T_cell <- RunPCA(T_cell, verbose = FALSE)
ElbowPlot(T_cell)
T_cell <- RunUMAP(T_cell, reduction = "pca", dims = 1:15)
T_cell <- RunTSNE(T_cell, reduction = "pca", dims = 1:15)
T_cell <- FindNeighbors(T_cell, reduction = "pca", dims = 1:15)
saveRDS(T_cell,file = "F:/scRNA/JCML/analysis3/20 2/celltype/T_cell/T_cell_15.RDS")
T_cell <- readRDS(file = "F:/scRNA/JCML/analysis3/20 2/celltype/T_cell/T_cell_15.RDS")

T_cell <- FindClusters(T_cell, resolution = 0.5)

saveRDS(T_cell,file = "F:/scRNA/JCML/analysis3/20 2/celltype/T_cell/T_cell_15_0.5.RDS")
T_cell <- readRDS(file = "F:/scRNA/JCML/analysis3/20 2/celltype/T_cell/T_cell_15_0.5.RDS")


p1 <- DimPlot(T_cell, reduction = "umap", group.by = "orig.ident")+theme(panel.background = element_blank(),panel.grid.major = element_blank(),panel.border = element_rect(colour="black",fill=NA))
p2 <- DimPlot(T_cell, reduction = "umap",label = TRUE, repel = TRUE,pt.size=1)+theme(panel.background = element_blank(),panel.grid.major = element_blank(),panel.border = element_rect(colour="black",fill=NA))
```

```
p2 + p1
p3 <- DimPlot(T_cell, reduction = "tsne", group.by = "orig.ident")+theme(panel.background = element_blank(),panel.grid.major = element_blank(),panel.border = element_rect(colour="black",fill=NA))
p4 <- DimPlot(T_cell, reduction = "tsne",label = TRUE, repel = TRUE,pt.size=1)+theme(panel.background = element_blank(),panel.grid.major = element_blank(),panel.border = element_rect(colour="black",fill=NA))
p4 + p3

DefaultAssay(T_cell) <- "RNA"

markers.to.plot <- c("CD3D","CD3E","CD3G","CD2","CD4","CD8A","CD8B","KLRF1","KLRD1",
                     "IKZF2","IL2RA","FOXP3",
                     "CCR7","LEF1","SELL","TCF7",
                     "IFNG","GZMB","PRF1","GZMK","GNLY","GZMA","NKG7",
                     "CTLA4","HAVCR2","LAG3","TIGIT","PDCD1",
                     "TNFRSF9","ICOS","TNFRSF14","CD28",
                     "MKI67")

#2021Cancer cell:Signatures of plasticity, metastasis, and immunosuppression in an atlas of human small cell lung cancer
#memory-like:"CD44","IL7R","GZMK","CD69","CD27"
#effector-like
markers.to.plot <-c("CD44","IL7R","GZMK","CD69","CD27",

"CTSW","GZMB","PRF1","GNLY","FGFBP2","FCGR3A","TRGC2")

VlnPlot(T_cell, features = markers.to.plot)

#cluster0 和 8 只有少部分是 T 细胞，故删除，不对其进行更多研究。
T_cell <- subset(T_cell, idents = c(0,8),invert=TRUE)
table(Idents(T_cell))

T_cell <- subset(T_cell, idents = c(0,8))
table(Idents(T_cell))
Cluster <- c("T_cell")
Celltype <- c("T_DELETE")
T_cell@meta.data$Celltype <- plyr::mapvalues(x = T_cell@meta.data$Cluster,from = Cluster, to = Celltype)
head(T_cell@meta.data)
table(T_cell@meta.data$Celltype)
```

```
Idents(T_cell)<-"Celltype"
saveRDS(T_cell,file="F:/scRNA/JCML/analysis3/20
2/celltype/T_cell_DELETE.RDS")
T_cell <- readRDS(file = "F:/scRNA/JCML/analysis3/20
2/celltype/T_cell_DELETE.RDS")


DefaultAssay(T_cell) <- "integrated"
T_cell <- RunPCA(T_cell, verbose = FALSE)
ElbowPlot(T_cell)
T_cell <- RunUMAP(T_cell, reduction = "pca", dims = 1:15)
T_cell <- RunTSNE(T_cell, reduction = "pca", dims = 1:15)
T_cell <- FindNeighbors(T_cell, reduction = "pca", dims = 1:15)
saveRDS(T_cell,file = "F:/scRNA/JCML/analysis3/20
2/celltype/T_cell/T_cell_15_0.5_D_15.RDS")
T_cell <- readRDS(file = "F:/scRNA/JCML/analysis3/20
2/celltype/T_cell/T_cell_15_0.5_D_15.RDS")

T_cell <- FindClusters(T_cell, resolution = 0.5)

saveRDS(T_cell,file = "F:/scRNA/JCML/analysis3/20
2/celltype/T_cell/T_cell_15_0.5_D_15_0.5.RDS")
T_cell <- readRDS(file = "F:/scRNA/JCML/analysis3/20
2/celltype/T_cell/T_cell_15_0.5_D_15_0.5.RDS")


p1 <- DimPlot(T_cell, reduction = "umap", group.by =
"orig.ident")+theme(panel.background =
element_blank(),panel.grid.major = element_blank(),panel.border =
element_rect(colour="black",fill=NA))
p2 <- DimPlot(T_cell, reduction = "umap",label = TRUE, repel =
TRUE,pt.size=1)+theme(panel.background =
element_blank(),panel.grid.major = element_blank(),panel.border =
element_rect(colour="black",fill=NA))
p2 + p1
p3 <- DimPlot(T_cell, reduction = "tsne", group.by =
"orig.ident")+theme(panel.background =
element_blank(),panel.grid.major = element_blank(),panel.border =
element_rect(colour="black",fill=NA))
p4 <- DimPlot(T_cell, reduction = "tsne",label = TRUE, repel =
TRUE,pt.size=1)+theme(panel.background =
element_blank(),panel.grid.major = element_blank(),panel.border =
element_rect(colour="black",fill=NA))
p4 + p3
```

```
DefaultAssay(T_cell) <- "RNA"
#We can explore these marker genes for each cluster and use them to annotate our clusters as specific cell types.
markers.to.plot <- c("CD3D","CD3E","CD3G","CD2","CD4","CD8A","CD8B","KLRF1","KLRD1",
                    "IKZF2","IL2RA","FOXP3",
                    "CCR7","LEF1","SELL","TCF7",
                    "IFNG","GZMB","PRF1","GZMK","GNLY","GZMA","NKG7",
                    "CTLA4","HAVCR2","LAG3","TIGIT","PDCD1",
                    "TNFRSF9","ICOS","TNFRSF14","CD28")
DotPlot(T_cell, features = markers.to.plot,cols = c("yellow", "red"))+
  theme(panel.background = element_blank(),panel.grid.major = element_blank(),panel.border = element_blank(),line = element_blank(),axis.title = element_blank(),axis.ticks.y = element_line(colour="black"),legend.position="top",legend.direction="horizontal",legend.text = element_text(size = 10),legend.title=element_text(size = 10))+coord_flip()
```

```
markers <- FindAllMarkers(T_cell,only.pos = TRUE)
top10 <- markers %>% group_by(cluster) %>% top_n(n = 10, wt = avg_log2FC)
DefaultAssay(T_cell) <- "integrated"
DoHeatmap(T_cell, features = top10$gene) + NoLegend()

#15 0.5
DefaultAssay(T_cell) <- "RNA"
cluster1 <- FindMarkers(T_cell, ident.1 =1,min.pct = 0.25)
write.csv(cluster1,file="F:/scRNA/JCML/analysis3/20
2/celltype/T_cell/15 0.5/cluster1_marker.csv")
cluster2 <- FindMarkers(T_cell, ident.1 =2)
write.csv(cluster2,file="F:/scRNA/JCML/analysis3/20
2/celltype/T_cell/15 0.5/cluster2_marker.csv")
cluster5 <- FindMarkers(T_cell, ident.1 =5,min.pct = 0.25)
write.csv(cluster5,file="F:/scRNA/JCML/analysis3/20
2/celltype/T_cell/15 0.5/cluster5_marker.csv")
```

```
VlnPlot(T_cell, features = c("nFeature_RNA", "nCount_RNA", "percent.mt", "percent.rb"),split.by = "orig.ident", ncol = 2)
```

```
VlnPlot(T_cell, features = c("nFeature_RNA", "nCount_RNA", "percent.mt",
"percent.rb"),ncol = 2)



VlnPlot(T_cell,                          features                          =
c("CTLA4","HAVCR2","LAG3","TIGIT","PDCD1","C10orf54"),group.by          =
"orig.ident")
VlnPlot(T_cell, features = c("XCL2",   "CCL5",    "ITGA1",   "KLRC1",
   "HOPX",    "CD160",   "CCL4",    "KLRD1",    "SPRY1",   "FKBP11"))

VlnPlot(T_cell, features = c("IL7R","ANXA1","CD6","SPOCK2","RORA"))

VlnPlot(T_cell, features = c("CD40LG","KLRC2","CD160","CD244"))


T_cell      <-      readRDS(file      =      "F:/scRNA/JCML/analysis3/20
2/celltype/T_cell/T_cell_15_0.5_D_15_0.5.RDS")
table(Idents(T_cell))

CD4_CCR7=c(0)
CD8_XCL2=c(1)
Treg_FOXP3= c(2)
DNT=c(3)
CD8_GZMK=c(4)
CD4_CD40LG=c(5)
NKT=c(6)

current.cluster.ids                                                     <-
c(CD8_XCL2,CD4_CCR7,CD4_CD40LG,Treg_FOXP3,CD8_GZMK,NKT,DNT)
new.cluster.ids <- c(rep("CD8_XCL2",length(CD8_XCL2)),
                     rep("CD4_CCR7",length(CD4_CCR7)),
                     rep("CD4_CD40LG",length(CD4_CD40LG)),
                     rep("Treg_FOXP3",length(Treg_FOXP3)),
                     rep("CD8_GZMK",length(CD8_GZMK)),
                     rep("NKT",length(NKT)),
                     rep("DNT",length(DNT)))

T_cell@meta.data$Celltype        <-        plyr::mapvalues(x         =
as.integer(as.character(T_cell@meta.data$seurat_clusters)),    from    =
current.cluster.ids, to = new.cluster.ids)
head(T_cell@meta.data)
table(T_cell@meta.data$Celltype)
```

```
T_cell$Celltype <-
factor(T_cell$Celltype ,level=c("CD8_XCL2","CD8_GZMK",

"CD4_CCR7","CD4_CD40LG","Treg_FOXP3",
                                                      "NKT","DNT"))
Idents(T_cell)<-"Celltype"
table(Idents(T_cell))

saveRDS(T_cell,file="F:/scRNA/JCML/analysis3/20 2/celltype/T_cell/15
0.5/celltype/T_cell_15_0.5_celltype.RDS")
T_cell <- readRDS(file="F:/scRNA/JCML/analysis3/20 2/celltype/T_cell/15
0.5/celltype/T_cell_15_0.5_celltype.RDS")

p1 <- DimPlot(T_cell, reduction = "umap", group.by =
"orig.ident")+theme(panel.background =
element_blank(),panel.grid.major = element_blank(),panel.border =
element_rect(colour="black",fill=NA))
p2 <- DimPlot(T_cell, reduction = "umap",label = TRUE, repel =
TRUE,pt.size=1)+theme(panel.background =
element_blank(),panel.grid.major = element_blank(),panel.border =
element_rect(colour="black",fill=NA))
p2 + p1
p3 <- DimPlot(T_cell, reduction = "tsne", group.by =
"orig.ident")+theme(panel.background =
element_blank(),panel.grid.major = element_blank(),panel.border =
element_rect(colour="black",fill=NA))
p4 <- DimPlot(T_cell, reduction = "tsne",label = TRUE, repel =
TRUE,pt.size=1)+theme(panel.background =
element_blank(),panel.grid.major = element_blank(),panel.border =
element_rect(colour="black",fill=NA))
p4 + p3


#cellcycle
T_cell <- readRDS(file="F:/scRNA/JCML/analysis3/20 2/celltype/T_cell/15
0.5/celltype/T_cell_15_0.5_celltype.RDS")
DefaultAssay(T_cell) <- "RNA"
#$s.genes
VlnPlot(T_cell, features =
c("MCM5","PCNA","TYMS","FEN1","MCM2","MCM4","RRM1","UNG","GINS2","MCM6
","CDCA7","DTL","PRIM1",

"UHRF1","MLF1IP","HELLS","RFC2","RPA2","NASP","RAD51AP1","GMNN","WDR76
","SLBP","CCNE2","UBR7",
```

```
"POLD3","MSH2","ATAD2","RAD51","RRM2","CDC45","CDC6","EXO1","TIPIN","DSCC1","BLM","CASP8AP2",

"USP1","CLSPN","POLA1","CHAF1B","BRIP1","E2F8"),pt.size = 0,ncol=7)
#$g2m.genes
VlnPlot(T_cell, features = c("HMGB2","CDK1","NUSAP1","UBE2C","BIRC5","TPX2","TOP2A","NDC80","CKS2","NUF2","CKS1B","MKI67",

"TMPO","CENPF","TACC3","FAM64A","SMC4","CCNB2","CKAP2L","CKAP2","AURKB","BUB1","KIF11","ANP32E",
                               "TUBB4B","GTSE1","KIF20B","HJURP",
"CDCA3","HN1","CDC20","TTK","CDC25C","KIF2C","RANGAP1","NCAPD2",

"DLGAP5","CDCA2","CDCA8","ECT2","KIF23","HMMR","AURKA","PSRC1","ANLN","LBR","CKAP5","CENPE","CTCF",

"NEK2","G2E3","GAS2L3","CBX5","CENPA"),pt.size = 0,ncol=7)

VlnPlot(T_cell, features = c("PCNA","MCM2","CCNE2",

"HMGB2","CDK1","TOP2A","MKI67","TUBB4B"),pt.size = 0,ncol=4)

T_cell <- CellCycleScoring(T_cell,
                           s.features = cc.genes.updated.2019$s.genes,
                           g2m.features = cc.genes.updated.2019$g2m.genes,
                           seed = 1,
                           set.ident =TRUE)

T_cell@meta.data %>% ggplot(aes(S.Score,G2M.Score))+geom_point(aes(color=Phase))+theme_minimal()


DimPlot(T_cell, reduction = "tsne",group.by = "Phase")+
  theme(panel.background = element_blank(),panel.grid.major = element_blank(),panel.border = element_rect(colour="black",fill=NA))

DimPlot(T_cell, reduction = "umap",group.by = "Phase",split.by = "orig.ident")+
  theme(panel.background = element_blank(),panel.grid.major = element_blank(),panel.border = element_rect(colour="black",fill=NA))
```

```
DimPlot(T_cell, reduction = "tsne",
        group.by = "Phase",
        pt.size =0.5)

DimPlot(T_cell, reduction = "pca",
        group.by = "orig.ident",
        shape.by = "Phase",
        pt.size =2)

DimPlot(T_cell, reduction = "tsne",
        group.by = "orig.ident",
        shape.by = "Phase")

DimPlot(T_cell, reduction = "umap",
        group.by = "orig.ident",
        shape.by = "Phase",
        pt.size =2)

#$s.genes
VlnPlot(T_cell,                        features                        =
c("MCM5","PCNA","TYMS","FEN1","MCM2","MCM4","RRM1","UNG","GINS2","MCM6
","CDCA7","DTL","PRIM1",

"UHRF1","MLF1IP","HELLS","RFC2","RPA2","NASP","RAD51AP1","GMNN","WDR76
","SLBP","CCNE2","UBR7",

"POLD3","MSH2","ATAD2","RAD51","RRM2","CDC45","CDC6","EXO1","TIPIN","D
SCC1","BLM","CASP8AP2",

"USP1","CLSPN","POLA1","CHAF1B","BRIP1","E2F8"),pt.size = 0,group.by =
"orig.ident")

VlnPlot(T_cell,                        features                        =
c("MCM5","PCNA","TYMS","FEN1","MCM2","MCM4","RRM1","UNG","GINS2","MCM6
","CDCA7","DTL","PRIM1",

"UHRF1","MLF1IP","HELLS","RFC2","RPA2","NASP","RAD51AP1","GMNN","WDR76
","SLBP","CCNE2","UBR7",

"POLD3","MSH2","ATAD2","RAD51","RRM2","CDC45","CDC6","EXO1","TIPIN","D
SCC1","BLM","CASP8AP2",

"USP1","CLSPN","POLA1","CHAF1B","BRIP1","E2F8"),pt.size = 0)
```

```
#$g2m.genes
VlnPlot(T_cell,                          features                          =
c("HMGB2","CDK1","NUSAP1","UBE2C","BIRC5","TPX2","TOP2A","NDC80","CKS2
","NUF2","CKS1B","MKI67",

"TMPO","CENPF","TACC3","FAM64A","SMC4","CCNB2","CKAP2L","CKAP2","AURKB
","BUB1","KIF11","ANP32E",
                             "TUBB4B","GTSE1","KIF20B","HJURP",
"CDCA3","HN1","CDC20","TTK","CDC25C","KIF2C","RANGAP1","NCAPD2",

"DLGAP5","CDCA2","CDCA8","ECT2","KIF23","HMMR","AURKA","PSRC1","ANLN",
"LBR","CKAP5","CENPE","CTCF",

"NEK2","G2E3","GAS2L3","CBX5","CENPA"),pt.size    =    0,group.by    =
"orig.ident")

VlnPlot(T_cell,                          features                          =
c("HMGB2","CDK1","NUSAP1","UBE2C","BIRC5","TPX2","TOP2A","NDC80","CKS2
","NUF2","CKS1B","MKI67",

"TMPO","CENPF","TACC3","FAM64A","SMC4","CCNB2","CKAP2L","CKAP2","AURKB
","BUB1","KIF11","ANP32E",
                             "TUBB4B","GTSE1","KIF20B","HJURP",
"CDCA3","HN1","CDC20","TTK","CDC25C","KIF2C","RANGAP1","NCAPD2",

"DLGAP5","CDCA2","CDCA8","ECT2","KIF23","HMMR","AURKA","PSRC1","ANLN",
"LBR","CKAP5","CENPE","CTCF",

"NEK2","G2E3","GAS2L3","CBX5","CENPA"),pt.size = 0)


#cell component
#proportion
Idents(T_cell)<-"Phase"

#orig.ident
table(T_cell$Celltype)
table(Idents(T_cell))
prop.table(table(Idents(T_cell)))
table(Idents(T_cell), T_cell$Celltype)
prop.table(table(Idents(T_cell), T_cell$Celltype), margin = 2)
T_cell_p<-as.data.frame(prop.table(table(Idents(T_cell),
T_cell@meta.data[,"Celltype"]), margin = 2))
```

```
#
ggplot(T_cell_p,aes(x=T_cell_p[,2],y=T_cell_p[,3],fill=T_cell_p[,1]))+
  geom_bar(position = 'stack',stat="identity")+
  labs(x="Celltype",y="Cell proportion")+
  theme(panel.background=element_rect(fill='transparent',
color='black'),panel.border =element_rect(fill=NA,color='black'),
        legend.key=element_rect(fill='transparent',
color='transparent'),axis.text = element_text(color="black"))+
  scale_y_continuous(expand=c(0.001,0.001))+
  guides(fill = guide_legend(keywidth = 1, keyheight = 1,ncol=1,title =
'Cell types'))+RotatedAxis()

#number

T_cell_N<-as.data.frame(table(Idents(T_cell),
T_cell@meta.data[,"Celltype"]), margin = 2)
#纵向
ggplot(T_cell_N,aes(x=T_cell_N[,2],y=T_cell_N[,3],fill=T_cell_N[,1]))+
  geom_bar(position = 'stack',stat="identity")+
  labs(x="Celltype",y="Cell number")+
  theme(panel.background=element_rect(fill='transparent',
color='black'),panel.border =element_rect(fill=NA,color='black'),
        legend.key=element_rect(fill='transparent',
color='transparent'),axis.text = element_text(color="black"))+
  scale_y_continuous(expand=c(0.001,0.001))+
  guides(fill = guide_legend(keywidth = 1, keyheight = 1,ncol=1,title =
'Cell types'))


#2
T_cell <- readRDS(file = "F:/scRNA/JCML/analysis3/20
2/celltype/T_cell/T_cell_15_0.5_D_15_0.5.RDS")
table(Idents(T_cell))

CD8_T=c(1,4)
CD4_T=c(0,5)
Treg_FOXP3= c(2)
DNT=c(3)
NKT=c(6)

current.cluster.ids <- c(CD8_T,CD4_T,Treg_FOXP3,NKT,DNT)
new.cluster.ids <- c(rep("CD8_T",length(CD8_T)),
                     rep("CD4_T",length(CD4_T)),
                     rep("Treg_FOXP3",length(Treg_FOXP3)),
```

```
                    rep("NKT",length(NKT)),
                    rep("DNT",length(DNT)))

T_cell@meta.data$Celltype        <-        plyr::mapvalues(x        =
as.integer(as.character(T_cell@meta.data$seurat_clusters)),   from   =
current.cluster.ids, to = new.cluster.ids)
head(T_cell@meta.data)
table(T_cell@meta.data$Celltype)

T_cell$Celltype <- factor(T_cell$Celltype ,level=c("CD8_T",

"CD4_T","Treg_FOXP3",
                                                  "NKT","DNT"))
Idents(T_cell)<-"Celltype"
table(Idents(T_cell))

saveRDS(T_cell,file="F:/scRNA/JCML/analysis3/20   2/celltype/T_cell/15
0.5/celltype/T_cell_15_0.5_celltype2.RDS")
T_cell <- readRDS(file="F:/scRNA/JCML/analysis3/20 2/celltype/T_cell/15
0.5/celltype/T_cell_15_0.5_celltype2.RDS")


p1    <-    DimPlot(T_cell,    reduction    =    "umap",    group.by    =
"orig.ident")+theme(panel.background                                   =
element_blank(),panel.grid.major   =   element_blank(),panel.border   =
element_rect(colour="black",fill=NA))
p2    <-    DimPlot(T_cell,    reduction    =    "umap",    repel    =
TRUE,pt.size=1)+theme(panel.background                                 =
element_blank(),panel.grid.major   =   element_blank(),panel.border   =
element_rect(colour="black",fill=NA))
p2 + p1
p3    <-    DimPlot(T_cell,    reduction    =    "tsne",    group.by    =
"orig.ident")+theme(panel.background                                   =
element_blank(),panel.grid.major   =   element_blank(),panel.border   =
element_rect(colour="black",fill=NA))
p4    <-    DimPlot(T_cell,    reduction    =    "tsne",    repel    =
TRUE,pt.size=1)+theme(panel.background                                 =
element_blank(),panel.grid.major   =   element_blank(),panel.border   =
element_rect(colour="black",fill=NA))
p4 + p3


#We can explore these marker genes for each cluster and use them to
annotate our clusters as specific cell types.
```

```
T_cell <- readRDS(file="F:/scRNA/JCML/analysis3/20 2/celltype/T_cell/15
0.5/celltype/T_cell_15_0.5_celltype.RDS")
DefaultAssay(T_cell) <- "RNA"
#dotplot used
markers.to.plot <-c("CD3E","CD3D","CD8A","CD8B","CD4","GNLY",
                    "IKZF2","IL2RA","FOXP3",
                    "CCR7","LEF1","SELL","TCF7",
                    "TNFRSF9","ICOS","TNFRSF14","CD28",
                    "IFNG","GZMB","PRF1","GZMK","GZMA","NKG7",
                    "CTLA4","HAVCR2","LAG3","TIGIT","PDCD1")
DotPlot(T_cell, features = markers.to.plot,cols = c("yellow", "red"))+
  theme(panel.background    =    element_blank(),panel.grid.major    =
element_blank(),panel.border = element_blank(),
        line          =          element_blank(),axis.title          =
element_blank(),axis.ticks.y = element_line(colour="black"),
        legend.position="top",legend.direction=
"horizontal",legend.text = element_text(size = 10),
        legend.title=element_text(size                                =
10))+coord_flip()+RotatedAxis()


markers.to.plot                                                      <-
c("CD3E","CD3D","CD8A","CD8B","CD4","GNLY","NKG7","KLRB1","KLRC1","KLR
D1",
                    "XCL2","GZMK","CCR7","CD40LG","FOXP3","MKI67")
#vln used
markers.to.plot <-c("CD3E","CD3D","CD8A","CD8B","CD4","GNLY","KLRD1",
                    "XCL2","GZMK","CCR7","CD40LG","FOXP3")


VlnPlot(T_cell, features = markers.to.plot,pt.size=0)

VlnPlot(T_cell,                       features                        =
c("PECAM1","VWF","RAMP2","CD34","DARC","CDH5"),pt.size=0)
##
markers.to.plot <-c("CD3D","CD3E","CD3G","CD4","CD8A","CD8B",
                    "IKZF2",
                    "IFNG","GZMB","PRF1","GZMK","GNLY","GZMA","NKG7")

markers.to.plot <-c("CD3D","CD4","CD8A","IKZF2",
                    "GZMB","PRF1","GZMA","NKG7")

VlnPlot(T_cell, features = markers.to.plot,pt.size=0,ncol = 4)
```

```
#"CTLA4"
VlnPlot(T_cell, features = "CTLA4",pt.size=0)
VlnPlot(T_cell, features = "CTLA4",pt.size=0,split.by = "orig.ident")+
  stat_compare_means(aes(split="orig.ident"),         label         =
"p.signif",method="t.test")
```

```
T_cell <- readRDS(file="F:/scRNA/JCML/analysis3/20 2/celltype/T_cell/15
0.5/celltype/T_cell_15_0.5_celltype.RDS")
T_cell <- readRDS(file="F:/scRNA/JCML/analysis3/20 2/celltype/T_cell/15
0.5/celltype/T_cell_15_0.5_celltype2.RDS")
DefaultAssay(T_cell) <- "RNA"
table(Idents(T_cell))

#cell component

#proportion
T_cell <- readRDS(file="F:/scRNA/JCML/analysis3/20 2/celltype/T_cell/15
0.5/celltype/T_cell_15_0.5_celltype.RDS")
table(T_cell$orig.ident)
table(Idents(T_cell))
prop.table(table(Idents(T_cell)))
table(Idents(T_cell), T_cell$orig.ident)
prop.table(table(Idents(T_cell), T_cell$orig.ident), margin = 2)

T_cell_p<-as.data.frame(prop.table(table(Idents(T_cell),
T_cell@meta.data[,"orig.ident"]), margin = 2))
#
ggplot(T_cell_p,aes(x=T_cell_p[,2],y=T_cell_p[,3],fill=T_cell_p[,1]))+
  geom_bar(position = 'stack',stat="identity")+
  labs(x="Sample",y="Cell proportion")+
  theme(panel.background=element_rect(fill='transparent',
color='black'),panel.border =element_rect(fill=NA,color='black'),
        legend.key=element_rect(fill='transparent',
color='transparent'),axis.text = element_text(color="black"))+
  scale_y_continuous(expand=c(0.001,0.001))+
  guides(fill = guide_legend(keywidth = 1, keyheight = 1,ncol=1,title =
'Cell types'))
```

```
#
ggplot(T_cell_p,aes(x=T_cell_p[,1],y=T_cell_p[,3]))+

geom_bar(stat="identity",aes(fill=T_cell_p[,2]),position=position_dodge(0.9))+
  labs(x="celltype",y="Cell proportion")+
  theme(panel.background=element_rect(fill='transparent',
color='black'),panel.border =element_rect(fill=NA,color='black'),
        legend.key=element_rect(fill='transparent',
color='transparent'),axis.text = element_text(color="black"))+
  scale_y_continuous(expand=c(0.001,0.001))+
  guides(fill = guide_legend(keywidth = 1, keyheight = 1,ncol=1,title =
'Sample'))



#CD8+T
T_cell <- readRDS(file="F:/scRNA/JCML/analysis3/20 2/celltype/T_cell/15 0.5/celltype/T_cell_15_0.5_celltype.RDS")
table(Idents(T_cell))
CD8 <- subset(T_cell,idents=c("CD8_GZMK","CD8_XCL2"))
saveRDS(CD8,file="F:/scRNA/JCML/analysis3/20       2/celltype/T_cell/15 0.5/celltype/T_cell_15_0.5_celltype_CD8.RDS")
CD8  <-  readRDS(file="F:/scRNA/JCML/analysis3/20  2/celltype/T_cell/15 0.5/celltype/T_cell_15_0.5_celltype_CD8.RDS")
table(Idents(CD8))

DefaultAssay(CD8) <- "RNA"



#
library(Seurat)
?AddModuleScore
library(tidyverse)
library(Matrix)
library(cowplot)
library(readxl)
scRNAsub          <-          readRDS(file="F:/scRNA/JCML/analysis3/20 2/celltype/T_cell/15 0.5/celltype/T_cell_15_0.5_celltype_CD8.RDS")
DefaultAssay(scRNAsub) <- "RNA"
table(Idents(scRNAsub))

scRNAsub <- subset(scRNAsub,idents=c("CD8_GZMK","CD8_XCL2"))
```

```
scRNAsub <- subset(T_cell,idents=c(1,3,4))

#CD8 T 活化
Activation <- read.csv("F:/scRNA/GCTB/site/150.8/celltype/TIL/celltype/CD8_T/score/GOBP_CD8_POSITIVE_ALPHA_BETA_T_CELL_ACTIVATION.csv")
#View(Activation)
#转换成list
gene <- as.list(Activation)

scRNAsub <- AddModuleScore(
  object = scRNAsub,
  features = gene,
  ctrl = 100,
  name = 'Activation_score',
  seed=1)

Cytotoxic <- readxl::read_xlsx("E:/single cell sequence/Score gene sets/immunocyte/GOBP_T_CELL_MEDIATED_CYTOTOXICITY.xlsx")
#View(Cytotoxic)
#转换成list
gene <- as.list(Cytotoxic)

scRNAsub <- AddModuleScore(
  object = scRNAsub,
  features = gene,
  ctrl = 100,
  name = 'Cytotoxic_GO',
  seed=1)

colnames(scRNAsub@meta.data)
# [1] "orig.ident" "nCount_RNA" "nFeature_RNA" "proj_name"
# [5] "percent.mt" "percent.HB" "integrated_snn_res.0.05" "seurat_clusters"
# [9] "integrated_snn_res.0.6" "integrated_snn_res.0.3" "meningioma_level" "sample_type"
# [13] "celltype" "RNA_snn_res.0.8" "RNA_snn_res.0.6" "RNA_snn_res.0.4"
# [17] "celltype_1" "RNA_snn_res.0.2" "CD_Features1"


colnames(scRNAsub@meta.data)[11] <- 'Activation_score'
```

```
ccolnames(scRNAsub@meta.data)[12] <- 'Cytotoxic_GO'
colnames(scRNAsub@meta.data)
#view(scRNAsub@meta.data)


data0<- FetchData(scRNAsub,vars = c("orig.ident","Celltype",
                                    "Activation_score","Cytotoxic_GO"))
write.csv(data0,file="F:/scRNA/JCML/analysis3/20   2/celltype/T_cell/15
0.5/celltype/CD8/score/data0.csv")
data0<-read.csv(file="F:/scRNA/JCML/analysis3/20   2/celltype/T_cell/15
0.5/celltype/CD8/score/data0.csv")

library(ggpubr)
ggviolin(data0, x = "orig.ident", y = "Activation_score",
         fill = "orig.ident", add = "boxplot",
         ylab = "Activation_score", xlab = "sample")+
  stat_compare_means(label ="p.format",method="t.test",hide.ns = FALSE)


ggviolin(data0, x = "orig.ident", y = "Cytotoxic_GO",
         fill = "orig.ident", add = "boxplot",
         ylab = "Cytotoxic_GO", xlab = "sample")+
  stat_compare_means(label ="p.format",method="t.test",hide.ns = FALSE)
```
