# Supplementary material for: Single-cell analysis reveals cellular reprogramming in advanced colon cancer following FOLFOX-bevacizumab treatment
Source: Front Oncol. 2023 Jul 28;13:1219642. doi: 10.3389/fonc.2023.1219642 (PMC10421721; doi:10.3389/fonc.2023.1219642)
Supplement: Supplementary file 3 [file DataSheet_1.zip › PDF/Figure5 and figureS3.pdf]

```r
library(ggplot2)
library(cowplot)
library(Seurat)
library(dplyr)
library(patchwork)
library(ggpubr)
rm(list = ls())

setwd("F:/scRNA/JCML/analysis3/20 2/celltype/B_cell/")

JCML.combined <-
readRDS(file="F:/scRNA/JCML/analysis3/JCML_combined_20_2_celltype_D.RDS")
B_cell <- subset(JCML.combined, idents = "B_cell")
B_cell

DefaultAssay(B_cell) <- "integrated"
B_cell <- RunPCA(B_cell,verbose = FALSE)
ElbowPlot(B_cell)

B_cell <- RunUMAP(B_cell, reduction = "pca", dims = 1:15)
B_cell <- RunTSNE(B_cell, reduction = "pca", dims = 1:15)
B_cell <- FindNeighbors(B_cell, reduction = "pca", dims = 1:15)
saveRDS(B_cell,file = "F:/scRNA/JCML/analysis3/20 2/celltype/B_cell/B_cell_15.RDS")
B_cell <- readRDS(file = "F:/scRNA/JCML/analysis3/20 2/celltype/B_cell/B_cell_15.RDS")

B_cell <- FindClusters(B_cell, resolution = 0.1)

saveRDS(B_cell,file = "F:/scRNA/JCML/analysis3/20 2/celltype/B_cell/B_cell_15_0.1.RDS")
B_cell <- readRDS(file = "F:/scRNA/JCML/analysis3/20 2/celltype/B_cell/B_cell_15_0.1.RDS")

p1 <- DimPlot(B_cell, reduction = "umap", group.by = "orig.ident")+theme(panel.background = element_blank(),panel.grid.major = element_blank(),panel.border = element_rect(colour="black",fill=NA))
p2 <- DimPlot(B_cell, reduction = "umap",label = TRUE, repel = TRUE,pt.size=1)+theme(panel.background = element_blank(),panel.grid.major = element_blank(),panel.border = element_rect(colour="black",fill=NA))
p2 + p1
```

```
p3 <- DimPlot(B_cell, reduction = "tsne", group.by = "orig.ident")+theme(panel.background = element_blank(),panel.grid.major = element_blank(),panel.border = element_rect(colour="black",fill=NA))
p4 <- DimPlot(B_cell, reduction = "tsne",label = TRUE, repel = TRUE,pt.size=1)+theme(panel.background = element_blank(),panel.grid.major = element_blank(),panel.border = element_rect(colour="black",fill=NA))
p4 + p3


DefaultAssay(B_cell) <- "RNA"

markers <- FindAllMarkers(B_cell,only.pos = TRUE, min.pct = 0.25, logfc.threshold =0.25)
top30 <- markers %>% group_by(cluster) %>% top_n(n = 30, wt = avg_log2FC)
DefaultAssay(B_cell) <- "integrated"
DoHeatmap(B_cell, features = top30$gene) + NoLegend()

DefaultAssay(B_cell) <- "RNA"

#B 细胞分群
#浆细胞杀伤肿瘤机制：1.secreting immunoglobulins to mediate the effects of antibody-dependent cell-mediated cytotoxicity(ADCC), antibody-dependent cellular phagocytosis (ADCP), and complement-dependent cytotoxicity (CDC); 2. antigen presentation to activate T cells; 3.secreting Granzyme B, tumor necrosis factorrelated apoptosis inducing ligand(TRAIL) and IFNγ to kill tumor cells directly
#文献：
#Naive B CELL: CD79/CD79A+,CD20/MS4A1+,CD27-,IgD+
#Memory B CELL: unswitched memory B:CD79 + CD20 +CD27 + IgD+;switched memory B:CD79 + CD20 + CD27 + IgD-
#Antibody secreting B CELL: plasmablast: CD20-CD27 + CD38 +CD138/SDC1-;plasma: CD20-CD27+ CD38 + CD138+
#mucosa-associated lymphoid tissue-derived plasma B cells(MALT B CELL):JCHAIN/IGJ,IGHA1
#GrB-secreting cells; GZMB
#GC B cells in the LZ:LMO2,BCL2A1
#GC B cells in the DZ:STMN1,AICDA,MKI67,BIRC5
#TRAIL/TNFSF10, IFNG

#R&D
#follicular B cells:CD10-,CD19+,MS4A1+,CD21/CR2(MID),CD22+,CD23/FCER2+,CD24/CD24A(LO
```

W),CD27-,CD38(LOW),CXCR5+,HLA-DR+,IgD(high),IgM(LOW),TACI/TNFRSF13B+
#plasma B cells:IGHG1,BCMA/TNFRSF17+,CD10/MME-,CD19-,CD20/MS4A1-,CD27+,CD38(High)， CXCR4+,HLA-DR(LOW),IgD-,CD138/SDC1(HIGH)
#Memory B CELL:CD80+,CD86+,CD10-,CD19+,CD20+,CD21+,CD23(LOW),CD27(Mid/+),CD38-,CD40+,CD93-,CD95/FAS+,CD148/PTPRJ+,HLA-DR+,TACI+
#Breg:
CD1D(HIGH),CD5+,CD19+,CD20-,CD21(HIGH),CD24(HIGH),CD27+/-,CD38(high),CD40+,IgD+/-,IgM(HIGH),IL10+,IL35/IL12A+,TGFB1+,TIM-1/HAVCR1+
#Marginal Zone B Cell:CD1C(HIGH),CD19+,CD20+,CD21(HIGH),CD23-/LOW,CD27+,FCRL3+,IgM+,IgD(LOW),TACI/TNFRSF13B+

```
markers.to.plot <-c("CD79A","MS4A1","CD27","IGHD",

"MME","CD19","CR2","CD22","FCER2","CD24","CXCR5","IGHM","TNFRSF13B",

"TNFRSF17","CD38","SDC1","CXCR4","IGHG1","IGHG2","IGHG3","IGHG4",
                    "CD80","CD86","CD40","CD93","FAS","PTPRJ",
                    "CD1D","CD5","IL10","IL12A","TGFB1","HAVCR1",
                    "CD1C",
                    "IGJ","IGHA1","IGHA2",
                    "GZMB","TNFSF10","IFNG",
                    "LMO2","BCL2A1",
                    "STMN1","AICDA","MKI67","BIRC5")

markers.to.plot <-c("IGHG1","IGHG2","IGHG3","IGHG4",
                    "IGHA1","IGHA2")
```

#2021Spatially-resolved transcriptomics analyses of invasive fronts in solid tumors
#na??ve B cells: MS4A1, CD19, CD22, TCL1A, CD83, BANK1, CD79A
#plasma B cells: MZB1, IGLL1, IGLL5, SSR4, JCHAIN/IGJ, IRF4, SDC1,XBP1, PRDM1
#memory B cells: FCRL4, CCR1, CD27, CD44, GPR183, CD69, CXCR4,CCR7, KLF2
#germinal center B cells: AICDA, RGS13, GCSAM, BCL6, NANS, CD81, CD38
#HLA-Ⅱ:HLA-DRB1, HLA-DQB1, HLA-DPB1, HLA-DRA, HLADQA1,HLA-DPA1, HLA-DRB5, HLA-DRB4, HLA-DQA2,HLA-DRB3, HLA-DOA, HLA-DMA, HLA-DMB, HLADQB2,HLA-DOB

```
markers.to.plot <-
c("CD19","CD79A","MS4A1","CD22","TCL1A","CD83","BANK1",

"MZB1","IGLL1","IGLL5","SSR4","IGJ","IRF4","SDC1","XBP1","PRDM1",
```

```
"FCRL4","CCR1","CD27","CD44","GPR183","CD69","CXCR4","CCR7","KLF2",

"AICDA","RGS13","GCSAM","BCL6","NANS","CD81","CD38",
                    "VEGFA","VEGFB","VEGFC","VEGFD")
DotPlot(B_cell, features = markers.to.plot,cols = c("yellow", "red"))+
  theme(panel.background = element_blank(),panel.grid.major = element_blank(),panel.border = element_blank(),line = element_blank(),axis.title = element_blank(),axis.ticks.y = element_line(colour="black"),legend.position="top",legend.direction= "horizontal",legend.text = element_text(size = 10),legend.title=element_text(size = 10))+coord_flip()

VlnPlot(B_cell, features = markers.to.plot,pt.size = 0,ncol=6)


VlnPlot(B_cell, features = c("nFeature_RNA", "nCount_RNA", "percent.mt", "percent.rb"),split.by = "orig.ident", ncol = 2)
VlnPlot(B_cell, features = c("nFeature_RNA", "nCount_RNA", "percent.mt", "percent.rb"),ncol = 2)

#VEGF
VlnPlot(B_cell, features = c("VEGFA","VEGFB","VEGFC"),pt.size = 0)

VlnPlot(B_cell, features = c("VEGFA","VEGFB","VEGFC"),pt.size = 0,split.by = "orig.ident")

VlnPlot(B_cell, features = c("VEGFA","VEGFB","VEGFC"),pt.size = 0,group.by = "orig.ident")


# find markers for every cluster compared to all remaining cells
B_cell.markers <- FindAllMarkers(B_cell, only.pos = TRUE, min.pct = 0.25, logfc.threshold =0.25)
top10 <- B_cell.markers %>% group_by(cluster) %>% top_n(n = 10, wt = avg_log2FC)
DefaultAssay(B_cell) <- "integrated"
DoHeatmap(B_cell, features = top10$gene) + NoLegend()


#15 0.1
#细胞类型注释 1
B_cells <- readRDS(file = "F:/scRNA/JCML/analysis3/202/celltype/B_cell/B_cell_15_0.1.RDS")
table(Idents(B_cells))
```

```
Plasma_cell=c(0,1)
B_cell=c(2)
Cycling_cell= c(3)

current.cluster.ids <- c(Plasma_cell,B_cell,Cycling_cell)
new.cluster.ids <- c(rep("Plasma_cell",length(Plasma_cell)),
                     rep("B_cell",length(B_cell)),
                     rep("Cycling_cell",length(Cycling_cell)))

B_cells@meta.data$Celltype        <-        plyr::mapvalues(x        =
as.integer(as.character(B_cells@meta.data$seurat_clusters)),   from   =
current.cluster.ids, to = new.cluster.ids)
head(B_cell@meta.data)
table(B_cells@meta.data$Celltype)

B_cells$Celltype                                                     <-
factor(B_cells$Celltype ,level=c("B_cell","Plasma_cell","Cycling_cell"
))
Idents(B_cells)<-"Celltype"
table(Idents(B_cells))

saveRDS(B_cells,file="F:/scRNA/JCML/analysis3/20  2/celltype/B_cell/15
0.1/celltype/B_cell_15_0.1_celltype.RDS")
B_cell <- readRDS(file="F:/scRNA/JCML/analysis3/20 2/celltype/B_cell/15
0.1/celltype/B_cell_15_0.1_celltype.RDS")

#细胞类型注释 2
B_cells     <-     readRDS(file     =     "F:/scRNA/JCML/analysis3/20
2/celltype/B_cell/B_cell_15_0.1.RDS")
table(Idents(B_cells))


Plasma_cell_vegf=c(0)
Plasma_cell=c(1)
B_Naive=c(2)
Cycling_cell= c(3)

current.cluster.ids                                                  <-
c(Plasma_cell_vegf,Plasma_cell,B_Naive,Cycling_cell)
new.cluster.ids <- c(rep("Plasma_cell_vegf",length(Plasma_cell_vegf)),
                     rep("Plasma_cell",length(Plasma_cell)),
                     rep("B_Naive",length(B_Naive)),
```

```
                    rep("Cycling_cell",length(Cycling_cell)))

B_cells@meta.data$Celltype <- plyr::mapvalues(x = as.integer(as.character(B_cells@meta.data$seurat_clusters)), from = current.cluster.ids, to = new.cluster.ids)
head(B_cell@meta.data)
table(B_cells@meta.data$Celltype)

B_cells$Celltype <- factor(B_cells$Celltype ,level=c("B_Naive","Plasma_cell_vegf","Plasma_cell","Cycling_cell"))
Idents(B_cells)<-"Celltype"
table(Idents(B_cells))

saveRDS(B_cells,file="F:/scRNA/JCML/analysis3/20  2/celltype/B_cell/15 0.1/celltype/B_cell_15_0.1_celltype2.RDS")
B_cell <- readRDS(file="F:/scRNA/JCML/analysis3/20 2/celltype/B_cell/15 0.1/celltype/B_cell_15_0.1_celltype2.RDS")

p1 <- DimPlot(B_cell, reduction = "umap", group.by = "orig.ident")+theme(panel.background = element_blank(),panel.grid.major = element_blank(),panel.border = element_rect(colour="black",fill=NA))
p2 <- DimPlot(B_cell, reduction = "umap", repel = TRUE,pt.size=1)+theme(panel.background = element_blank(),panel.grid.major = element_blank(),panel.border = element_rect(colour="black",fill=NA))
p2 + p1
p3 <- DimPlot(B_cell, reduction = "tsne", group.by = "orig.ident")+theme(panel.background = element_blank(),panel.grid.major = element_blank(),panel.border = element_rect(colour="black",fill=NA))
p4 <- DimPlot(B_cell, reduction = "tsne", repel = TRUE,pt.size=1)+theme(panel.background = element_blank(),panel.grid.major = element_blank(),panel.border = element_rect(colour="black",fill=NA))
p4 + p3

#We can explore these marker genes for each cluster and use them to annotate our clusters as specific cell types.
DefaultAssay(B_cell) <- "RNA"
markers.to.plot <-c("MS4A1","BANK1",
                    "MZB1","IGJ",
                    "VEGFA","VEGFB",
```

```
                    "MKI67","STMN1")

DotPlot(B_cell, features = markers.to.plot, dot.scale = 8) +
  theme(panel.background    =    element_blank(),panel.grid.major    =
element_blank(),panel.border                                         =
element_rect(colour="black",fill=NA))+coord_flip()+
  RotatedAxis()

VlnPlot(B_cell, features = markers.to.plot,pt.size = 0,ncol=3)
FeaturePlot(B_cell,features = markers.to.plot,reduction ="umap",ncol=2)



#亚群差异基因
B.markers <- FindAllMarkers(B_cell, only.pos = TRUE)
B.markers
write.csv(B.markers,file="F:/scRNA/JCML/analysis3/20
2/celltype/B_cell/15 0.1/celltype/celltype2/B_marker.csv")



top20  <-  B.markers  %>%  group_by(cluster)  %>%  top_n(n  =  20,  wt  =
avg_log2FC)
top15  <-  B.markers  %>%  group_by(cluster)  %>%  top_n(n  =  15,  wt  =
avg_log2FC)
DefaultAssay(B_cell) <- "integrated"
DoHeatmap(B_cell, features = top15$gene) + NoLegend()
DoHeatmap(B_cell, features = top20$gene)


#cell component
#proportion
B_cell <- readRDS(file="F:/scRNA/JCML/analysis3/20 2/celltype/B_cell/15
0.1/celltype/B_cell_15_0.1_celltype.RDS")
B_cell <- readRDS(file="F:/scRNA/JCML/analysis3/20 2/celltype/B_cell/15
0.1/celltype/B_cell_15_0.1_celltype2.RDS")
table(B_cell$orig.ident)
table(Idents(B_cell))
prop.table(table(Idents(B_cell)))
table(Idents(B_cell), B_cell$orig.ident)
prop.table(table(Idents(B_cell), B_cell$orig.ident), margin = 2)

B_cell_p<-as.data.frame(prop.table(table(Idents(B_cell),
B_cell@meta.data[,"orig.ident"]), margin = 2))
```

```
#堆砌条形图
ggplot(B_cell_p,aes(x=B_cell_p[,2],y=B_cell_p[,3],fill=B_cell_p[,1]))+
  geom_bar(position = 'stack',stat="identity")+
  labs(x="Sample",y="Cell proportion")+
  theme(panel.background=element_rect(fill='transparent',
color='black'),panel.border =element_rect(fill=NA,color='black'),
        legend.key=element_rect(fill='transparent',
color='transparent'),axis.text = element_text(color="black"))+
  scale_y_continuous(expand=c(0.001,0.001))+
  guides(fill = guide_legend(keywidth = 1, keyheight = 1,ncol=1,title =
'Cell types'))

#分组条形图
ggplot(B_cell_p,aes(x=B_cell_p[,1],y=B_cell_p[,3]))+

geom_bar(stat="identity",aes(fill=B_cell_p[,2]),position=position_dodg
e(0.9))+
  labs(x="celltype",y="Cell proportion")+
  theme(panel.background=element_rect(fill='transparent',
color='black'),panel.border =element_rect(fill=NA,color='black'),
        legend.key=element_rect(fill='transparent',
color='transparent'),axis.text = element_text(color="black"))+
  scale_y_continuous(expand=c(0.001,0.001))+
  guides(fill = guide_legend(keywidth = 1, keyheight = 1,ncol=1,title =
'Sample'))


#monocle
library(monocle)
library(Seurat)
library(dplyr)
library(patchwork)
library(tidyverse)
library(stringr)
library(metap)


SeuratObject         <-          readRDS(file="F:/scRNA/JCML/analysis3/20
2/celltype/B_cell/15 0.1/celltype/B_cell_15_0.1_celltype2.RDS")

SeuratObject@meta.data
table(SeuratObject$orig.ident)
table(Idents(SeuratObject))
```

```
data <- as(as.matrix(SeuratObject@assays$RNA@counts), 'sparseMatrix')
pd<-new("AnnotatedDataFrame", data = SeuratObject@meta.data)
fd<-new("AnnotatedDataFrame", data = data.frame(gene_short_name = row.names(data), row.names = row.names(data)))
cds <- newCellDataSet(data, phenoData = pd, featureData = fd)
#对 monocle 对象进行归一化
cds <- estimateSizeFactors(cds)
cds <- estimateDispersions(cds)
#过滤掉低质量的基因
cds <- detectGenes(cds,min_expr = 0.1)
print(head(fData(cds)))
expressed_genes <- row.names(subset(fData(cds),
                                    num_cells_expressed >= 10))
print(head(pData(cds)))
#Trajectory step 1: choose genes that define a cell's progress:cluster

diff_test_res <- differentialGeneTest(cds[expressed_genes,],
                                       fullModelFormulaStr = '~Celltype')

ordering_genes <- row.names (subset(diff_test_res, qval < 0.01))
cds <- setOrderingFilter(cds, ordering_genes)
plot_ordering_genes(cds)
#Trajectory step 2: reduce data dimensionality
cds <- reduceDimension(cds, reduction_method = "DDRTree")
#Trajectory step 3: order cells along the trajectory
cds <- orderCells(cds)
plot_cell_trajectory(cds, color_by = "Pseudotime")##根据拟时间值着色
plot_cell_trajectory(cds, color_by = "State")####以 state 进行着色
plot_cell_trajectory(cds, color_by = "Celltype")##如果有 Seurat 生的 rds 文件的话，按照 seurat 中分的群进行着色，如果不想用 ggplot 的默认色，可以提供颜色列表 col list。
plot_cell_trajectory(cds, color_by = "Celltype") + facet_wrap(~orig.ident,nrow=1)
plot_cell_trajectory(cds, color_by = "Celltype") + facet_wrap(~Celltype, nrow=2)
plot_cell_trajectory(cds, color_by = "Celltype") + facet_wrap(vars(orig.ident,Celltype),nrow=2)

plot_cell_trajectory(cds, color_by = "seurat_clusters")##如果有 Seurat 生的 rds 文件的话，按照 seurat 中分的群进行着色，如果不想用 ggplot 的默认色，可以提供颜色列表 col list。
plot_cell_trajectory(cds, color_by = "seurat_clusters") + facet_wrap(~orig.ident,nrow=1)
```

```
plot_cell_trajectory(cds,     color_by     =     "seurat_clusters")     +
facet_wrap(~seurat_clusters, nrow=2)
plot_cell_trajectory(cds,     color_by     =     "seurat_clusters")     +
facet_wrap(vars(orig.ident,seurat_clusters))

plot_cell_trajectory(cds, color_by = "orig.ident")


saveRDS(cds,file="F:/scRNA/JCML/analysis3/20       2/celltype/B_cell/15
0.1/celltype/monocle/monocle_celltype2.RDS")
cds  <-  readRDS(file="F:/scRNA/JCML/analysis3/20  2/celltype/B_cell/15
0.1/celltype/monocle/monocle_celltype2.RDS")


#used
to_be_tested <- row.names(subset(fData(cds),
                                 gene_short_name                   %in%
c("CD19","CD79A","MS4A1","CD22","TCL1A","CD83","BANK1",

"MZB1","IGLL1","IGLL5","SSR4","IGJ","IRF4","SDC1","XBP1","PRDM1",

"MKI67","PCNA","TOP2A")))

cds_subset <- cds[to_be_tested,]
plot_pseudotime_heatmap(cds_subset,
                        cores = 1,
                        show_rownames = T,
                        num_clusters =3)

plot_pseudotime_heatmap(cds_subset,
                        cores = 1,
                        show_rownames = T,
                        num_clusters =1)
```
