# Supplementary material for: Single-cell analysis reveals cellular reprogramming in advanced colon cancer following FOLFOX-bevacizumab treatment
Source: Front Oncol. 2023 Jul 28;13:1219642. doi: 10.3389/fonc.2023.1219642 (PMC10421721; doi:10.3389/fonc.2023.1219642)
Supplement: Supplementary file 3 [file DataSheet_1.zip › PDF/Figure6 and figureS4.pdf]

```
#Fibroblast
library(ggplot2)
library(cowplot)
library(Seurat)
library(dplyr)
library(patchwork)
library(ggpubr)
rm(list=ls())

setwd("F:/scRNA/JCML/analysis3/20 2/celltype/Fibroblast/")

JCML.combined <-
readRDS(file="F:/scRNA/JCML/analysis3/JCML_combined_20_2_celltype_D.RD
S")
Fibroblast <- subset(JCML.combined, idents = "Fibroblast")
Fibroblast

DefaultAssay(Fibroblast) <- "integrated"
Fibroblast <- RunPCA(Fibroblast,verbose = FALSE)
ElbowPlot(Fibroblast)

Fibroblast <- RunUMAP(Fibroblast, reduction = "pca", dims = 1:15)
Fibroblast <- RunTSNE(Fibroblast, reduction = "pca", dims = 1:15)
Fibroblast <- FindNeighbors(Fibroblast, reduction = "pca", dims = 1:15)
saveRDS(Fibroblast,file = "F:/scRNA/JCML/analysis3/20
2/celltype/Fibroblast/Fibroblast_15.RDS")
Fibroblast <- readRDS(file = "F:/scRNA/JCML/analysis3/20
2/celltype/Fibroblast/Fibroblast_15.RDS")

Fibroblast <- FindClusters(Fibroblast, resolution = 0.3)

saveRDS(Fibroblast,file = "F:/scRNA/JCML/analysis3/20
2/celltype/Fibroblast/Fibroblast_15_0.3.RDS")
Fibroblast <- readRDS(file = "F:/scRNA/JCML/analysis3/20
2/celltype/Fibroblast/Fibroblast_15_0.3.RDS")


p1 <- DimPlot(Fibroblast, reduction = "umap", group.by =
"orig.ident")+theme(panel.background =
element_blank(),panel.grid.major = element_blank(),panel.border =
element_rect(colour="black",fill=NA))
p2 <- DimPlot(Fibroblast, reduction = "umap",label = TRUE, repel =
TRUE,pt.size=1)+theme(panel.background =
element_blank(),panel.grid.major = element_blank(),panel.border =
```

```
element_rect(colour="black",fill=NA))
p2 + p1
p3   <-   DimPlot(Fibroblast,   reduction   =   "tsne",   group.by   =
"orig.ident")+theme(panel.background                                =
element_blank(),panel.grid.major   =   element_blank(),panel.border   =
element_rect(colour="black",fill=NA))
p4 <- DimPlot(Fibroblast, reduction = "tsne",label = TRUE, repel =
TRUE,pt.size=1)+theme(panel.background                              =
element_blank(),panel.grid.major   =   element_blank(),panel.border   =
element_rect(colour="black",fill=NA))
p4 + p3


#15 0.3
Fibroblast         <-         readRDS(file="F:/scRNA/JCML/analysis3/20
2/celltype/Fibroblast/Fibroblast_15_0.3.RDS")

DefaultAssay(Fibroblast) <- "RNA"
cluster0 <- FindMarkers(Fibroblast, ident.1 =0,min.pct = 0.25)
write.csv(cluster0,file="F:/scRNA/JCML/analysis3/20
2/celltype/Fibroblast/15 0.3/cluster0_marker.csv")

#CAF_metabolism
cluster1 <- FindMarkers(Fibroblast, ident.1 =1,min.pct = 0.25)
write.csv(cluster1,file="F:/scRNA/JCML/analysis3/20
2/celltype/Fibroblast/15 0.3/cluster1_marker.csv")

cluster2 <- FindMarkers(Fibroblast, ident.1 =2,min.pct = 0.25)
write.csv(cluster2,file="F:/scRNA/JCML/analysis3/20
2/celltype/Fibroblast/15 0.3/cluster2_marker.csv")

cluster3 <- FindMarkers(Fibroblast, ident.1 =3,min.pct = 0.25)
write.csv(cluster3,file="F:/scRNA/JCML/analysis3/20
2/celltype/Fibroblast/15 0.3/cluster3_marker.csv")

#筛选基因集
INPUT    <-    "F:/scRNA/JCML/analysis3/20    2/celltype/Fibroblast/15
0.3/cluster0_marker.csv"
INPUT    <-    "F:/scRNA/JCML/analysis3/20    2/celltype/Fibroblast/15
0.3/cluster1_marker.csv"
INPUT    <-    "F:/scRNA/JCML/analysis3/20    2/celltype/Fibroblast/15
0.3/cluster2_marker.csv"
INPUT    <-    "F:/scRNA/JCML/analysis3/20    2/celltype/Fibroblast/15
0.3/cluster3_marker.csv"
```

```
df <- read.csv(file=INPUT)
dim(df)
df_fc2 <- df[df$avg_log2FC >0.5,]
df_fc2 <- df[df$avg_log2FC >1,]
df_fc2 <- df[df$avg_log2FC < -0.5,]
df_fc2 <- df[df$avg_log2FC < -1,]
dim(df_fc2)
df_fc2_p0.05 <- df_fc2[df_fc2$p_val < 0.05,]
dim(df_fc2_p0.05)

write.csv(df_fc2_p0.05,file="F:/scRNA/JCML/analysis3/20
2/celltype/Fibroblast/15 0.3/cluster0_marker_fc0.5_p0.05.csv")
write.csv(df_fc2_p0.05,file="F:/scRNA/JCML/analysis3/20
2/celltype/Fibroblast/15 0.3/cluster0_marker_fc1_p0.05.csv")

write.csv(df_fc2_p0.05,file="F:/scRNA/JCML/analysis3/20
2/celltype/Fibroblast/15 0.3/cluster1_marker_fc0.5_p0.05.csv")

write.csv(df_fc2_p0.05,file="F:/scRNA/JCML/analysis3/20
2/celltype/Fibroblast/15 0.3/cluster2_marker_fc0.5_p0.05.csv")
write.csv(df_fc2_p0.05,file="F:/scRNA/JCML/analysis3/20
2/celltype/Fibroblast/15 0.3/cluster2_marker_fc1_p0.05.csv")

write.csv(df_fc2_p0.05,file="F:/scRNA/JCML/analysis3/20
2/celltype/Fibroblast/15 0.3/cluster3_marker_fc0.5_p0.05.csv")
write.csv(df_fc2_p0.05,file="F:/scRNA/JCML/analysis3/20
2/celltype/Fibroblast/15 0.3/cluster3_marker_fc1_p0.05.csv")

#clusterProfiler GO analysis
install.packages("tweenr")
library("clusterProfiler")
library("AnnotationDbi")
library("BiocGenerics")
library("Biobase")
library("IRanges")
library("S4Vectors")
library("parallel")
library("stats4")
library("org.Hs.eg.db")
library("enrichplot")
library("ggplot2")
library("DOSE")
library("GO.db")
```

df <- read.csv("F:/scRNA/JCML/analysis3/20 2/celltype/Fibroblast/15 0.3/cluster0_marker_fc0.5_p0.05.csv",header = TRUE)
df <- read.csv("F:/scRNA/JCML/analysis3/20 2/celltype/Fibroblast/15 0.3/cluster0_marker_fc1_p0.05.csv",header = TRUE)

df <- read.csv("F:/scRNA/JCML/analysis3/20 2/celltype/Fibroblast/15 0.3/cluster1_marker_fc0.5_p0.05.csv",header = TRUE)

df <- read.csv("F:/scRNA/JCML/analysis3/20 2/celltype/Fibroblast/15 0.3/cluster2_marker_fc0.5_p0.05.csv",header = TRUE)
df <- read.csv("F:/scRNA/JCML/analysis3/20 2/celltype/Fibroblast/15 0.3/cluster2_marker_fc1_p0.05.csv",header = TRUE)

df <- read.csv("F:/scRNA/JCML/analysis3/20 2/celltype/Fibroblast/15 0.3/cluster3_marker_fc0.5_p0.05.csv",header = TRUE)
df <- read.csv("F:/scRNA/JCML/analysis3/20 2/celltype/Fibroblast/15 0.3/cluster3_marker_fc1_p0.05.csv",header = TRUE)

df
x <- df[,2]
x
#将"SYMBOL"转换成"ENTREZID",(上述向量即为"SYMBOL"，基因名)
gene <- bitr(x, fromType="SYMBOL", toType="ENTREZID", OrgDb= "org.Hs.eg.db")
#进行 GO 富集分析。org.Hs.eg.db 是参考的数据库；"ALL"：进行三种分析，包括 BP CC MF；readable = T：最后输出的结果为可读的基因名；可修改 qvalue，因为相对于 pvalue 更严苛；pvaluecutoff 不能过大，不然出来的结果不可靠。
ego<- enrichGO(gene=gene$ENTREZID,OrgDb = "org.Hs.eg.db",keyType = "ENTREZID",ont = "ALL",readable = T,pvalueCutoff =0.05,qvalueCutoff = 1,)
#保存富集结果为 csv 格式

write.csv(ego,file="F:/scRNA/JCML/analysis3/20 2/celltype/Fibroblast/15 0.3/cluster0_marker_fc0.5_p0.05_GO.csv")
write.csv(ego,file="F:/scRNA/JCML/analysis3/20 2/celltype/Fibroblast/15 0.3/cluster0_marker_fc1_p0.05_GO.csv")

write.csv(ego,file="F:/scRNA/JCML/analysis3/20 2/celltype/Fibroblast/15 0.3/cluster1_marker_fc0.5_p0.05_GO.csv")

write.csv(ego,file="F:/scRNA/JCML/analysis3/20 2/celltype/Fibroblast/15 0.3/cluster2_marker_fc0.5_p0.05_GO.csv")

```
write.csv(ego,file="F:/scRNA/JCML/analysis3/20
2/celltype/Fibroblast/15 0.3/cluster2_marker_fc1_p0.05_GO.csv")

write.csv(ego,file="F:/scRNA/JCML/analysis3/20
2/celltype/Fibroblast/15 0.3/cluster3_marker_fc0.5_p0.05_GO.csv")
write.csv(ego,file="F:/scRNA/JCML/analysis3/20
2/celltype/Fibroblast/15 0.3/cluster3_marker_fc1_p0.05_GO.csv")

#气泡图
dotplot(ego,showCategory =50,title="cluster3_fc0_GO")
#柱状图
barplot(ego,showCategory =30,title="cluster0_fc0.5_GO")
barplot(ego,showCategory =30,title="cluster1_fc0.5_GO")
barplot(ego,showCategory =30,title="cluster2_fc0.5_GO")
barplot(ego,showCategory =30,title="cluster3_fc0.5_GO")



#used
#ggplot2 作图
library(ggplot2)

##CAF_metabolism
#Upregulated in CAF_metabolism
df          <-          read.csv(file="F:/scRNA/JCML/analysis3/20
2/celltype/Fibroblast/15
0.3/cluster1_marker_fc0.5_p0.05_GO_used_CAF_metabolism.csv")
df
ggplot(data = df) +
  geom_bar(aes(-log10(pvalue),reorder(Description,              -
log10(pvalue)), ),stat = "identity",fill="red") +
  theme(panel.background = element_blank(),
        panel.grid.major = element_blank(),
        panel.border = element_rect(colour="black",fill=NA))+
  labs(y="Pathway", x="-Log10(pval)",
       title="Upregulated in CAF_metabolism")

#15 0.3
#细胞类型注释
Fibroblasts    <-    readRDS(file    =    "F:/scRNA/JCML/analysis3/20
2/celltype/Fibroblast/Fibroblast_15_0.3.RDS")
table(Idents(Fibroblasts))


CAF_ECM=c(0)
```

```
CAF_metabolism=c(1)
CAF_contractile= c(2)
CAF_secretory= c(3)

current.cluster.ids                                                        <-
c(CAF_ECM,CAF_metabolism,CAF_contractile,CAF_secretory)
new.cluster.ids <- c(rep("CAF_ECM",length(CAF_ECM)),
                     rep("CAF_metabolism",length(CAF_metabolism)),
                     rep("CAF_contractile",length(CAF_contractile)),
                     rep("CAF_secretory",length(CAF_secretory)))

Fibroblasts@meta.data$Celltype       <-       plyr::mapvalues(x       =
as.integer(as.character(Fibroblasts@meta.data$seurat_clusters)),   from
= current.cluster.ids,  to = new.cluster.ids)

table(Fibroblasts@meta.data$Celltype)

Fibroblasts$Celltype                                                   <-
factor(Fibroblasts$Celltype ,level=c("CAF_ECM","CAF_contractile","CAF_
secretory","CAF_metabolism"))
Idents(Fibroblasts)<-"Celltype"
table(Idents(Fibroblasts))

saveRDS(Fibroblasts,file="F:/scRNA/JCML/analysis3/20
2/celltype/Fibroblast/15 0.3/celltype/Fibroblast_15_0.3_celltype.RDS")
Fibroblast         <-          readRDS(file="F:/scRNA/JCML/analysis3/20
2/celltype/Fibroblast/15 0.3/celltype/Fibroblast_15_0.3_celltype.RDS")

p1   <-   DimPlot(Fibroblast,   reduction   =   "umap",   group.by   =
"orig.ident")+theme(panel.background                                  =
element_blank(),panel.grid.major   =   element_blank(),panel.border   =
element_rect(colour="black",fill=NA))
p2   <-   DimPlot(Fibroblast,    reduction    =    "umap",    repel    =
TRUE,pt.size=1)+theme(panel.background                                =
element_blank(),panel.grid.major   =   element_blank(),panel.border   =
element_rect(colour="black",fill=NA))
p2 + p1
p3   <-   DimPlot(Fibroblast,   reduction   =   "tsne",   group.by   =
"orig.ident")+theme(panel.background                                  =
element_blank(),panel.grid.major   =   element_blank(),panel.border   =
element_rect(colour="black",fill=NA))
p4   <-   DimPlot(Fibroblast,    reduction    =    "tsne",    repel    =
TRUE,pt.size=1)+theme(panel.background                                =
element_blank(),panel.grid.major   =   element_blank(),panel.border   =
```

```
element_rect(colour="black",fill=NA))
p4 + p3

#We can explore these marker genes for each cluster and use them to annotate our clusters as specific cell types.
DefaultAssay(Fibroblast) <- "RNA"

markers.to.plot <-c("COL1A1","COL3A1","MMP2","MMP14",
                    "ACTA2","MYH11",

"IGF1","FGF7","VEGFA","VEGFB","CCL13","CCL11","CXCL1")

DotPlot(Fibroblast, features = markers.to.plot, dot.scale = 8) +
  theme(panel.background = element_blank(),panel.grid.major = element_blank(),panel.border = element_rect(colour="black",fill=NA))+coord_flip()+
  RotatedAxis()

VlnPlot(Fibroblast, features = markers.to.plot,pt.size = 0)



VlnPlot(Fibroblast, features = c("VEGFB"),pt.size = 0,group.by = "orig.ident",slot = "counts")+
  stat_compare_means(label ="p.signif" ,method="t.test",hide.ns = FALSE)

VlnPlot(Fibroblast, features = c("CCL13"),pt.size = 0,group.by = "orig.ident",slot = "counts")+
  stat_compare_means(label ="p.signif" ,method="t.test",hide.ns = FALSE)

VlnPlot(Fibroblast, features = c("CXCL1"),pt.size = 0,group.by = "orig.ident",slot = "counts")+
  stat_compare_means(label ="p.signif" ,method="t.test",hide.ns = FALSE)


#cell component
#proportion
Fibroblast <- readRDS(file="F:/scRNA/JCML/analysis3/202/celltype/Fibroblast/15 0.3/celltype/Fibroblast_15_0.3_celltype.RDS")
table(Fibroblast$orig.ident)
table(Idents(Fibroblast))
prop.table(table(Idents(Fibroblast)))
table(Idents(Fibroblast), Fibroblast$orig.ident)
prop.table(table(Idents(Fibroblast), Fibroblast$orig.ident), margin = 2)
```

```
Fibroblast_p<-as.data.frame(prop.table(table(Idents(Fibroblast), Fibroblast@meta.data[,"orig.ident"]), margin = 2))
#堆砌条形图
ggplot(Fibroblast_p,aes(x=Fibroblast_p[,2],y=Fibroblast_p[,3],fill=Fibroblast_p[,1]))+
  geom_bar(position = 'stack',stat="identity")+
  labs(x="Sample",y="Cell proportion")+
  theme(panel.background=element_rect(fill='transparent', color='black'),panel.border =element_rect(fill=NA,color='black'),
        legend.key=element_rect(fill='transparent', color='transparent'),axis.text = element_text(color="black"))+
  scale_y_continuous(expand=c(0.001,0.001))+
  guides(fill = guide_legend(keywidth = 1, keyheight = 1,ncol=1,title = 'Cell types'))
```
