# Supplementary material for: Single-cell analysis reveals cellular reprogramming in advanced colon cancer following FOLFOX-bevacizumab treatment
Source: Front Oncol. 2023 Jul 28;13:1219642. doi: 10.3389/fonc.2023.1219642 (PMC10421721; doi:10.3389/fonc.2023.1219642)
Supplement: Supplementary file 3 [file DataSheet_1.zip › PDF/Figure7 and figureS5.pdf]

```
library(Seurat)
library(tidyverse)
library(patchwork)
rm(list=ls())

library(ComplexHeatmap)
library(CellChat)
library(patchwork)
library(ggplot2)
library(ggalluvial)
library(svglite)
library(Seurat)
library(SeuratData)
library(mindr)
library(NMF)
library(future)
options(stringsAsFactors = FALSE)


CML.combined <-
readRDS(file="F:/scRNA/JCML/analysis3/JCML_combined_celltype.rds")
JCML.combined
table(JCML.combined@meta.data$orig.ident)
table(JCML.combined@meta.data$Cluster)

#一.Naive
Naive <- subset(JCML.combined,subset = orig.ident == "Naive")
Naive
table(Naive@meta.data$orig.ident)
table(Naive@meta.data$Cluster)
Naive@meta.data

data.input  <- Naive@assays$RNA@data
identity = data.frame(group =Naive$Cluster , row.names =
names(Naive$Cluster)) # create a dataframe consisting of the cell labels
unique(identity$group) # check the cell labels
cellchat_Naive <- createCellChat(data.input,Naive@meta.data,group.by =
"Cluster")
cellchat_Naive
summary(cellchat_Naive)

cellchat_Naive <- addMeta(cellchat_Naive, meta = identity, meta.name =
"labels")
cellchat_Naive <- setIdent(cellchat_Naive, ident.use = "labels") # set
```

```
"labels" as default cell identity
levels(cellchat_Naive@idents) # show factor levels of the cell labels
cellchat_Naive@idents <-
factor(cellchat_Naive@idents,level=c("Cancer_cell",

"T_cell","B_cell","Myeloid_cell",

"Fibroblast","Endotheliocyte"))

groupSize <- as.numeric(table(cellchat_Naive@idents)) # number of cells
in each cell group

#CellChatDB <- CellChatDB.human
#showDatabaseCategory(CellChatDB)
options(stringsAsFactors = FALSE)
interaction_input <- read.csv(file = 'E:/single cell sequence/Cellchat
代 码 /Update CellChatDB/human/interaction_input_CellChatDB.csv',
row.names = 1)
complex_input <- read.csv(file = 'E:/single cell sequence/Cellchat 代码
/Update CellChatDB/human/complex_input_CellChatDB.csv', row.names = 1)
cofactor_input <- read.csv(file = 'E:/single cell sequence/Cellchat 代
码/Update CellChatDB/human/cofactor_input_CellChatDB.csv', row.names =
1)
geneInfo <- read.csv(file = 'E:/single cell sequence/Cellchat 代码
/Update CellChatDB/human/geneInfo_input_CellChatDB.csv', row.names = 1)
CellChatDB <- list()
CellChatDB$interaction <- interaction_input
CellChatDB$complex <- complex_input
CellChatDB$cofactor <- cofactor_input
CellChatDB$geneInfo <- geneInfo


# Show the structure of the database
dplyr::glimpse(CellChatDB$interaction)

I <- dplyr::glimpse(CellChatDB$interaction)
view(I)


# use all CellChatDB for cell-cell communication analysis
CellChatDB.use <- CellChatDB # simply use the default CellChatD

cellchat_Naive@DB <- CellChatDB.use # set the used database in the
object
```

```
cellchat_Naive <- subsetData(cellchat_Naive) # subset the expression data of signaling genes for saving computation cost
future::plan("multiprocess", workers = 4)

cellchat_Naive <- identifyOverExpressedGenes(cellchat_Naive)
cellchat_Naive <- identifyOverExpressedInteractions(cellchat_Naive)
cellchat_Naive <- projectData(cellchat_Naive, PPI.human)

#cellchat_Naive3
cellchat_Naive <- computeCommunProb(cellchat_Naive,population.size = TRUE)
cellchat_Naive <- computeCommunProbPathway(cellchat_Naive)
cellchat_Naive <- aggregateNet(cellchat_Naive)


#cellchat_Naive3:cellchat_Naive <- computeCommunProb(cellchat_Naive,population.size = TRUE)
saveRDS(cellchat_Naive,file="F:/scRNA/JCML/analysis3/202/celltype/Cellchat/cellchat_Naive3.RDS")
cellchat_Naive <- readRDS(file="F:/scRNA/JCML/analysis3/202/celltype/Cellchat/cellchat_Naive3.RDS")



#二.Treat
Treat <- subset(JCML.combined,subset = orig.ident == "Treat")
Treat
table(Treat@meta.data$orig.ident)
table(Treat@meta.data$Cluster)

data.input  <- Treat@assays$RNA@data
identity = data.frame(group =Treat$Cluster, row.names = names(Treat$Cluster)) # create a dataframe consisting of the cell labels
unique(identity$group) # check the cell labels
cellchat_Treat <- createCellChat(data.input,Treat@meta.data,group.by = "Cluster")
cellchat_Treat
summary(cellchat_Treat)

cellchat_Treat <- addMeta(cellchat_Treat, meta = identity, meta.name = "labels")
cellchat_Treat <- setIdent(cellchat_Treat, ident.use = "labels") # set "labels" as default cell identity
```

```
levels(cellchat_Treat@idents) # show factor levels of the cell labels
cellchat_Treat@idents <-
factor(cellchat_Treat@idents,level=c("Cancer_cell",

"T_cell","B_cell","Myeloid_cell",

"Fibroblast","Endotheliocyte"))

groupSize <- as.numeric(table(cellchat_Treat@idents)) # number of cells in each cell group


options(stringsAsFactors = FALSE)
interaction_input <- read.csv(file = 'E:/single cell sequence/Cellchat 代码/Update CellChatDB/human/interaction_input_CellChatDB.csv', row.names = 1)
complex_input <- read.csv(file = 'E:/single cell sequence/Cellchat 代码/Update CellChatDB/human/complex_input_CellChatDB.csv', row.names = 1)
cofactor_input <- read.csv(file = 'E:/single cell sequence/Cellchat 代码/Update CellChatDB/human/cofactor_input_CellChatDB.csv', row.names = 1)
geneInfo <- read.csv(file = 'E:/single cell sequence/Cellchat 代码/Update CellChatDB/human/geneInfo_input_CellChatDB.csv', row.names = 1)
CellChatDB <- list()
CellChatDB$interaction <- interaction_input
CellChatDB$complex <- complex_input
CellChatDB$cofactor <- cofactor_input
CellChatDB$geneInfo <- geneInfo


CellChatDB.use <- CellChatDB # simply use the default CellChatD

cellchat_Treat@DB <- CellChatDB.use # set the used database in the object

cellchat_Treat <- subsetData(cellchat_Treat) # subset the expression data of signaling genes for saving computation cost
future::plan("multiprocess", workers = 4)

cellchat_Treat <- identifyOverExpressedGenes(cellchat_Treat)
cellchat_Treat <- identifyOverExpressedInteractions(cellchat_Treat)
cellchat_Treat <- projectData(cellchat_Treat, PPI.human)

#cellchat_Treat3
```

```
cellchat_Treat <- computeCommunProb(cellchat_Treat,population.size = TRUE)

cellchat_Treat <- computeCommunProbPathway(cellchat_Treat)
cellchat_Treat <- aggregateNet(cellchat_Treat)

#cellchat_Treat3:cellchat_Treat <- computeCommunProb(cellchat_Treat,population.size = TRUE)
saveRDS(cellchat_Treat,file="F:/scRNA/JCML/analysis3/20
2/celltype/Cellchat/cellchat_Treat3.RDS")
cellchat_Treat <- readRDS(file="F:/scRNA/JCML/analysis3/20
2/celltype/Cellchat/cellchat_Treat3.RDS")



#三。compare
library(igraph)
cellchat_Naive <- readRDS(file="F:/scRNA/JCML/analysis3/20
2/celltype/Cellchat/cellchat_Naive3.RDS")
cellchat_Treat <- readRDS(file="F:/scRNA/JCML/analysis3/20
2/celltype/Cellchat/cellchat_Treat3.RDS")


levels(cellchat_Treat@idents)
levels(cellchat_Naive@idents)
object.list <- list(Naive = cellchat_Naive, Treat = cellchat_Treat)
cellchat <- mergeCellChat(object.list, add.names = names(object.list))
# Define the cell labels to lift up
cellchat@idents <- factor(cellchat@idents,level=c("Cancer_cell",

"T_cell","B_cell","Myeloid_cell",

"Fibroblast","Endotheliocyte"))

group.new = levels(cellchat@idents)
cellchat_Naive <- liftCellChat(cellchat_Naive, group.new)
cellchat_Treat <- liftCellChat(cellchat_Treat, group.new)
levels(cellchat_Treat@idents)
levels(cellchat_Naive@idents)

object.list <- list(Naive = cellchat_Naive, Treat = cellchat_Treat)
cellchat <- mergeCellChat(object.list, add.names = names(object.list))
#> Merge the following slots: 'data.signaling','net', 'netP','meta', 'idents', 'var.features' , 'DB', and 'LR'.
```

```
cellchat

#cellchat_Naive3\cellchat_Treat3
saveRDS(cellchat,file="F:/scRNA/JCML/analysis3/20
2/celltype/Cellchat/cellchat3_merge.RDS")
cellchat         <-         readRDS(file="F:/scRNA/JCML/analysis3/20
2/celltype/Cellchat/cellchat3_merge.RDS")



#Part I: Predict general principles of cell-cell communication

#Compare the total number of interactions and interaction strength
gg1 <- compareInteractions(cellchat, show.legend = F, group = c(1,2))
gg2 <- compareInteractions(cellchat, show.legend = F, group = c(1,2),
measure = "weight")
gg1 + gg2


#Differential number of interactions or interaction strength among
different cell populations
#The differential number of interactions or interaction strength in the
cell-cell communication network between two datasets can be visualized
using circle plot, where red (or blue) colored edges represent increased
(or decreased) signaling in the second dataset compared to the first
one.

#treat vs naive
par(mfrow = c(1,2), xpd=TRUE)
netVisual_diffInteraction(cellchat, weight.scale = T,comparison =
c("Naive","Treat"))
netVisual_diffInteraction(cellchat, weight.scale = T, measure =
"weight",comparison = c("Naive","Treat"))

#We can also show differential number of interactions or interaction
strength in a greater details using a heatmap. The top colored bar plot
represents the sum of column of values displayed in the heatmap (incoming
signaling). The right colored bar plot represents the sum of row of
values (outgoing signaling). In the colorbar, red (or blue) represents
increased (or decreased) signaling in the second dataset compared to
the first one.
#treat vs naive
gg1 <- netVisual_heatmap(cellchat,comparison = c("Naive","Treat"))
#> Do heatmap based on a merged object
```

```
gg2 <- netVisual_heatmap(cellchat, measure = "weight",comparison = c("Naive","Treat"))
#> Do heatmap based on a merged object
gg1 + gg2


#The differential network analysis only works for pairwise datasets.
#If there are more datasets for comparison, we can directly show the number of interactions or interaction strength between any two cell populations in each dataset.

#To better control the node size and edge weights of the inferred networks across different datasets, we compute the maximum number of cells per cell group and the maximum number of interactions (or interaction weights) across all datasets.
weight.max <- getMaxWeight(object.list, attribute = c("idents","count"))
par(mfrow = c(1,2), xpd=TRUE)
for (i in 1:length(object.list)) {
  netVisual_circle(object.list[[i]]@net$count, weight.scale = T, label.edge= F, edge.weight.max = weight.max[2], edge.width.max = 12, title.name = paste0("Number of interactions - ", names(object.list)[i]))
}

weight.max <- getMaxWeight(object.list, attribute = c("idents","weight"))
par(mfrow = c(1,2), xpd=TRUE)
for (i in 1:length(object.list)) {
  netVisual_circle(object.list[[i]]@net$weight, weight.scale = T, label.edge= F, edge.weight.max = weight.max[2], edge.width.max = 12, title.name = paste0("Interaction strength - ", names(object.list)[i]))
}

#Compare the major sources and targets in 2D space
num.link <- sapply(object.list, function(x) {rowSums(x@net$count) + colSums(x@net$count)-diag(x@net$count)})
weight.MinMax <- c(min(num.link), max(num.link)) # control the dot size in the different datasets
gg <- list()
for (i in 1:length(object.list)) {
  gg[[i]] <- netAnalysis_signalingRole_scatter(object.list[[i]], title = names(object.list)[i], weight.MinMax = weight.MinMax)
}
#> Signaling role analysis on the aggregated cell-cell communication network from all signaling pathways
```

```
patchwork::wrap_plots(plots = gg)



#Part IV: Visually compare cell-cell communication using Hierarchy plot, Circle plot or Chord diagram

pathways.show <- c("VEGF")
weight.max <- getMaxWeight(object.list, slot.name = c("netP"), attribute = pathways.show) # control the edge weights across different datasets
#circle
par(mfrow = c(1,2), xpd=TRUE)
for (i in 1:length(object.list)) {
  netVisual_aggregate(object.list[[i]], signaling = pathways.show, layout = "circle", edge.weight.max = weight.max[1], edge.width.max = 10, signaling.name = paste(pathways.show, names(object.list)[i]))
}

#heatmap
ht <- list()
for (i in 1:length(object.list)) {
  ht[[i]] <- netVisual_heatmap(object.list[[i]], signaling = pathways.show, color.heatmap = "Reds",title.name = paste(pathways.show, "signaling ",names(object.list)[i]))
}
ComplexHeatmap::draw(ht[[1]] + ht[[2]], ht_gap = unit(0.5, "cm"))

# Chord diagram
for (i in 1:length(object.list)) {
  netVisual_aggregate(object.list[[i]], signaling = pathways.show, layout = "chord", signaling.name = paste(pathways.show, names(object.list)[i]))
}



#

netVisual_bubble(cellchat, signaling = "VEGF",sources.use = c("Cancer_cell",

"B_cell","Myeloid_cell",

"Fibroblast","Endotheliocyte"),
```

```
                 targets.use ="Endotheliocyte",angle.x = 45,comparison
= c(1, 2),remove.isolate = FALSE,n.colors = 3)

#Part V: Compare the signaling gene expression distribution between
different datasets
cellchat@meta$datasets  =  factor(cellchat@meta$datasets,  levels  =
c("Naive","Treat")) # set factor level

plotGeneExpression(cellchat, signaling = "VEGF",split.by = "datasets",
colors.ggplot = T,
                   idents =c("Cancer_cell",
                             "B_cell","Myeloid_cell",
                             "Fibroblast","Endotheliocyte"))
```

```
library(Seurat)
library(tidyverse)
library(patchwork)
rm(list=ls())

#Others
JCML.combined                                                     <-
readRDS(file="F:/scRNA/JCML/analysis3/JCML_combined_20_2_celltype_D.RD
S")
table(Idents(JCML.combined))
others           <-           subset(JCML.combined,idents            =
c("Fibroblast","Endotheliocyte","Cancer_cell"))
others@meta.data
Cluster <- c("Fibroblast","Endotheliocyte","Cancer_cell")
Celltype <- c("Fibroblast","Endotheliocyte","Cancer_cell")
others@meta.data$Celltype        <-        plyr::mapvalues(x         =
others@meta.data$Cluster,from = Cluster, to = Celltype)
head(others@meta.data)
table(others@meta.data$Celltype)
table(Idents(others))
Idents(others)<-"Celltype"
saveRDS(others,file="F:/scRNA/JCML/analysis3/20
2/celltype/others_annotation")
others     <-     readRDS(file     =     "F:/scRNA/JCML/analysis3/20
2/celltype/others_annotation")
```

```
JCML.combined <-
readRDS(file="F:/scRNA/JCML/analysis3/JCML_combined_20_2_celltype_D.RD
S")
TIL <- readRDS(file="F:/scRNA/JCML/analysis3/20 2/celltype/T_cell/15
0.5/celltype/T_cell_15_0.5_celltype2.RDS")
TIL_DELETE <- readRDS(file = "F:/scRNA/JCML/analysis3/20
2/celltype/T_cell_DELETE.RDS")
Myeloid <- readRDS(file = "F:/scRNA/JCML/analysis3/20
2/celltype/Myeloid/15 0.1/celltype/Myeloid_15_0.1_Celltype2")
BIL <- readRDS(file="F:/scRNA/JCML/analysis3/20 2/celltype/B_cell/15
0.1/celltype/B_cell_15_0.1_celltype.RDS")
others <- readRDS(file = "F:/scRNA/JCML/analysis3/20
2/celltype/others_annotation")

celltype_TIL = data.frame(ClusterID=rownames(TIL@meta.data),
celltype=TIL@meta.data$Celltype, stringsAsFactors = F)
celltype_TIL_DELETE =
data.frame(ClusterID=rownames(TIL_DELETE@meta.data),
celltype=TIL_DELETE@meta.data$Celltype, stringsAsFactors = F)
celltype_Myeloid = data.frame(ClusterID=rownames(Myeloid@meta.data),
celltype=Myeloid@meta.data$Celltype, stringsAsFactors = F)
celltype_BIL = data.frame(ClusterID=rownames(BIL@meta.data),
celltype=BIL@meta.data$Celltype, stringsAsFactors = F)
celltype_others = data.frame(ClusterID=rownames(others@meta.data),
celltype=others@meta.data$Celltype, stringsAsFactors = F)

celltype <-
rbind(celltype_TIL,celltype_TIL_DELETE,celltype_Myeloid,celltype_BIL,c
elltype_others)

JCML.combined@meta.data$CB = "NA"
#celltype_all = data.frame(ClusterID=rownames(JCML.combined@meta.data),
celltype=JCML.combined@meta.data$celltype_HumanPrimaryCellAtlasData,
stringsAsFactors = F)
celltype_all = data.frame(ClusterID=rownames(JCML.combined@meta.data),
celltype=JCML.combined@meta.data$celltype, stringsAsFactors = F)

JCML.combined@meta.data <- cbind(JCML.combined@meta.data,celltype_all)
for(i in 1:nrow(celltype_all)){
  JCML.combined@meta.data[which(JCML.combined@meta.data$ClusterID ==
celltype_all$ClusterID[i]),'celltype'] <- celltype_all$celltype[i]}
for(i in 1:nrow(celltype)){
  JCML.combined@meta.data[which(JCML.combined@meta.data$ClusterID ==
```

```
celltype$ClusterID[i]),'celltype'] <- celltype$celltype[i]}

view(JCML.combined@meta.data)

JCML.combined$celltype <-
factor(JCML.combined$celltype,level=c("Cancer_cell",

"CD4_T","Treg_FOXP3","CD8_T","NKT","DNT","T_DELETE",

"B_cell","Plasma_cell","Cycling_cell",

"TAM","Neutrophil","Mast_cell",

"Fibroblast","Endotheliocyte"))

Idents(JCML.combined) <- "celltype"
JCML.combined <- subset(JCML.combined,idents=c("T_DELETE"),invert=T)
JCML.combined <- subset(JCML.combined,subset= celltype ==
"T_DELETE",invert=T)
table(JCML.combined@meta.data$celltype)
table(Idents(JCML.combined))
saveRDS(JCML.combined,
file="F:/scRNA/JCML/analysis3/JCML_combined_celltype.rds")


JCML.combined <-
readRDS(file="F:/scRNA/JCML/analysis3/JCML_combined_celltype.rds")
Idents(JCML.combined) <- "Cluster"
# Visualization
p1 <- DimPlot(JCML.combined, reduction = "umap", group.by =
"orig.ident")+theme(panel.background =
element_blank(),panel.grid.major = element_blank(),panel.border =
element_rect(colour="black",fill=NA))
p2 <- DimPlot(JCML.combined, reduction = "umap", repel =
TRUE)+theme(panel.background = element_blank(),panel.grid.major =
element_blank(),panel.border = element_rect(colour="black",fill=NA))
p1 + p2

p3 <- DimPlot(JCML.combined, reduction = "tsne", group.by =
"orig.ident")+theme(panel.background =
element_blank(),panel.grid.major = element_blank(),panel.border =
element_rect(colour="black",fill=NA))
p4 <- DimPlot(JCML.combined, reduction = "tsne",repel =
TRUE,pt.size=1)+theme(panel.background =
```

```
element_blank(),panel.grid.major  =  element_blank(),panel.border  =
element_rect(colour="black",fill=NA))
p3 + p4

DefaultAssay(JCML.combined) <- "RNA"

markers.to.plot <- c("EPCAM","KRT19","KRT18",
                     "CD3D","CD3E","CD2",
                     "MS4A1","CD79A","MZB1",
                     "CD14", "CD68","TYROBP",
                     "DCN","COL1A1","THY1",
                     "RAMP2","CD34","CDH5")

VlnPlot(JCML.combined, features = markers.to.plot,ncol=5,pt.size = 0)

DotPlot(JCML.combined, features = markers.to.plot, dot.scale = 8) +
  theme(panel.background   =   element_blank(),panel.grid.major   =
element_blank(),panel.border                                      =
element_rect(colour="black",fill=NA))+coord_flip()+
  RotatedAxis()


#cell component
#proportion
JCML.combined                                                     <-
readRDS(file="F:/scRNA/JCML/analysis3/JCML_combined_celltype.rds")
Idents(JCML.combined) <- "Cluster"

table(JCML.combined$orig.ident)
table(Idents(JCML.combined))
prop.table(table(Idents(JCML.combined)))
table(Idents(JCML.combined), JCML.combined$orig.ident)
prop.table(table(Idents(JCML.combined),     JCML.combined$orig.ident),
margin = 2)
JCML.combined_p<-as.data.frame(prop.table(table(Idents(JCML.combined),
JCML.combined@meta.data[,"orig.ident"]), margin = 2))

ggplot(JCML.combined_p,aes(x=JCML.combined_p[,2],y=JCML.combined_p[,3]
,fill=JCML.combined_p[,1]))+
  geom_bar(position = 'stack',stat="identity")+
  labs(x="Sample",y="Cell proportion")+
  theme(panel.background=element_rect(fill='transparent',
color='black'),panel.border =element_rect(fill=NA,color='black'),
        legend.key=element_rect(fill='transparent',
```

```
color='transparent'),axis.text = element_text(color="black"))+
  scale_y_continuous(expand=c(0.001,0.001))+
  guides(fill = guide_legend(keywidth = 1, keyheight = 1,ncol=1,title =
'Cell types'))

ggplot(JCML.combined_p,aes(x=JCML.combined_p[,1],y=JCML.combined_p[,3]
))+

geom_bar(stat="identity",aes(fill=JCML.combined[,2]),position=position
_dodge(0.9))+
  labs(x="celltype",y="Cell proportion")+
  theme(panel.background=element_rect(fill='transparent',
color='black'),panel.border =element_rect(fill=NA,color='black'),
        legend.key=element_rect(fill='transparent',
color='transparent'),axis.text = element_text(color="black"))+
  scale_y_continuous(expand=c(0.001,0.001))+
  guides(fill = guide_legend(keywidth = 1, keyheight = 1,ncol=1,title =
'Sample'))

#cell number
JCML.combined_n<-as.data.frame(table(Idents(JCML.combined),
JCML.combined@meta.data[,"orig.ident"]), margin = 2)
ggplot(JCML.combined_n,aes(x=JCML.combined_n[,2],y=JCML.combined_n[,3]
,fill=JCML.combined_n[,1]))+
  geom_bar(position = 'stack',stat="identity")+
  labs(x="Sample",y="Cell Number")+
  theme(panel.background=element_rect(fill='transparent',
color='black'),panel.border =element_rect(fill=NA,color='black'),
        legend.key=element_rect(fill='transparent',
color='transparent'),axis.text = element_text(color="black"))+
  scale_y_continuous(expand=c(0.001,0.001))+
  guides(fill = guide_legend(keywidth = 1, keyheight = 1,ncol=1,title =
'Cell types'))

library(ComplexHeatmap)
library(CellChat)
library(patchwork)
library(ggplot2)
library(ggalluvial)
library(svglite)
library(Seurat)
library(SeuratData)
library(mindr)
library(NMF)
```

```
library(future)
options(stringsAsFactors = FALSE)


setwd("F:/scRNA/JCML/analysis3/20 2/celltype/Cellchat/")
JCML.combined                                                   <-
readRDS(file="F:/scRNA/JCML/analysis3/JCML_combined_celltype.rds")
JCML.combined
table(JCML.combined@meta.data$orig.ident)
table(JCML.combined@meta.data$celltype)

#一.Naive
Naive <- subset(JCML.combined,subset = orig.ident == "Naive")
Naive
table(Naive@meta.data$orig.ident)
table(Naive@meta.data$celltype)
Naive@meta.data

data.input  <- Naive@assays$RNA@data
identity    =    data.frame(group    =Naive$celltype    ,    row.names    =
names(Naive$celltype)) # create a dataframe consisting of the cell
labels
unique(identity$group) # check the cell labels
cellchat_Naive <- createCellChat(data.input,Naive@meta.data,group.by =
"celltype")
cellchat_Naive
summary(cellchat_Naive)

cellchat_Naive <- addMeta(cellchat_Naive, meta = identity, meta.name =
"labels")
cellchat_Naive <- setIdent(cellchat_Naive, ident.use = "labels") # set
"labels" as default cell identity
levels(cellchat_Naive@idents) # show factor levels of the cell labels
cellchat_Naive@idents                                           <-
factor(cellchat_Naive@idents,level=c("Cancer_cell",

"CD4_T","Treg_FOXP3","CD8_T","NKT","DNT",

"B_cell","Plasma_cell","Cycling_cell",

"TAM","Neutrophil","Mast_cell",

"Fibroblast","Endotheliocyte"))
```

```
groupSize <- as.numeric(table(cellchat_Naive@idents)) # number of cells in each cell group

options(stringsAsFactors = FALSE)
interaction_input <- read.csv(file = 'E:/single cell sequence/Cellchat 代码/Update CellChatDB/human/interaction_input_CellChatDB.csv', row.names = 1)
complex_input <- read.csv(file = 'E:/single cell sequence/Cellchat 代码/Update CellChatDB/human/complex_input_CellChatDB.csv', row.names = 1)
cofactor_input <- read.csv(file = 'E:/single cell sequence/Cellchat 代码/Update CellChatDB/human/cofactor_input_CellChatDB.csv', row.names = 1)
geneInfo <- read.csv(file = 'E:/single cell sequence/Cellchat 代码/Update CellChatDB/human/geneInfo_input_CellChatDB.csv', row.names = 1)
CellChatDB <- list()
CellChatDB$interaction <- interaction_input
CellChatDB$complex <- complex_input
CellChatDB$cofactor <- cofactor_input
CellChatDB$geneInfo <- geneInfo


# Show the structure of the database
dplyr::glimpse(CellChatDB$interaction)

I <- dplyr::glimpse(CellChatDB$interaction)
view(I)


CellChatDB.use <- CellChatDB # simply use the default CellChatD

cellchat_Naive@DB <- CellChatDB.use # set the used database in the object

cellchat_Naive <- subsetData(cellchat_Naive) # subset the expression data of signaling genes for saving computation cost
future::plan("multiprocess", workers = 4)
cellchat_Naive <- identifyOverExpressedGenes(cellchat_Naive)
cellchat_Naive <- identifyOverExpressedInteractions(cellchat_Naive)
cellchat_Naive <- projectData(cellchat_Naive, PPI.human)

#cellchat_Naive1
cellchat_Naive <- computeCommunProb(cellchat_Naive,population.size = TRUE)
cellchat_Naive <- computeCommunProbPathway(cellchat_Naive)
```

```
cellchat_Naive <- aggregateNet(cellchat_Naive)

# Compute the network centrality scores
cellchat_Naive <- netAnalysis_computeCentrality(cellchat_Naive, slot.name = "netP") # the slot 'netP' means the inferred intercellular communication network of signaling pathways

#cellchat_Naive1:cellchat_Naive <- computeCommunProb(cellchat_Naive)
saveRDS(cellchat_Naive,file="F:/scRNA/JCML/analysis3/20
2/celltype/Cellchat/cellchat_Naive1.RDS")
cellchat_Naive <- readRDS(file="F:/scRNA/JCML/analysis3/20
2/celltype/Cellchat/cellchat_Naive1.RDS")



pathways.show <-
c("PERIOSTIN","ncWNT","CD86","TWEAK","GAS","CD47","THY1","CCL","CSF","
ANGPT",
                   "PROS","FASLG","APELIN")

#
netVisual_bubble(cellchat_Naive, signaling = pathways.show, remove.isolate = FALSE)

pathways.show <- c("PERIOSTIN")
pathways.show <- c("ncWNT")
pathways.show <- c("CD86")
pathways.show <- c("TWEAK")
pathways.show <- c("GAS")
pathways.show <- c("CD47")
pathways.show <- c("THY1")
pathways.show <- c("CCL")
pathways.show <- c("CSF")
pathways.show <- c("ANGPT")
pathways.show <- c("PROS")
pathways.show <- c("FASLG")
pathways.show <- c("APELIN")


# Chord diagram
par(mfrow=c(1,3))
netVisual_aggregate(cellchat_Naive, signaling = pathways.show, layout = "chord")
```

```
# Circle plot
par(mfrow=c(1,1))
netVisual_aggregate(cellchat_Naive, signaling = pathways.show, layout = "circle")

# Heatmap
par(mfrow=c(3,1))
netVisual_heatmap(cellchat_Naive, signaling = pathways.show, color.heatmap = "Reds")

#
netVisual_bubble(cellchat_Naive, signaling = pathways.show, remove.isolate = FALSE)

#二.Treat
Treat <- subset(JCML.combined,subset = orig.ident == "Treat")
Treat
table(Treat@meta.data$orig.ident)
table(Treat@meta.data$celltype)


data.input  <- Treat@assays$RNA@data
identity = data.frame(group =Treat$celltype   , row.names = names(Treat$celltype)) # create a dataframe consisting of the cell labels
unique(identity$group) # check the cell labels
cellchat_Treat <- createCellChat(data.input,Treat@meta.data,group.by = "celltype")
cellchat_Treat
summary(cellchat_Treat)

cellchat_Treat <- addMeta(cellchat_Treat, meta = identity, meta.name = "labels")
cellchat_Treat <- setIdent(cellchat_Treat, ident.use = "labels") # set "labels" as default cell identity
levels(cellchat_Treat@idents) # show factor levels of the cell labels
cellchat_Treat@idents <- factor(cellchat_Treat@idents,level=c("Cancer_cell",

"CD4_T","Treg_FOXP3","CD8_T","NKT","DNT",

"B_cell","Plasma_cell","Cycling_cell",

"TAM","Neutrophil","Mast_cell",
```

```
"Fibroblast","Endotheliocyte"))

groupSize <- as.numeric(table(cellchat_Treat@idents)) # number of cells
in each cell group


options(stringsAsFactors = FALSE)
interaction_input <- read.csv(file = 'E:/single cell sequence/Cellchat
代 码 /Update CellChatDB/human/interaction_input_CellChatDB.csv',
row.names = 1)
complex_input <- read.csv(file = 'E:/single cell sequence/Cellchat 代码
/Update CellChatDB/human/complex_input_CellChatDB.csv', row.names = 1)
cofactor_input <- read.csv(file = 'E:/single cell sequence/Cellchat 代
码/Update CellChatDB/human/cofactor_input_CellChatDB.csv', row.names =
1)
geneInfo <- read.csv(file = 'E:/single cell sequence/Cellchat 代码
/Update CellChatDB/human/geneInfo_input_CellChatDB.csv', row.names = 1)
CellChatDB <- list()
CellChatDB$interaction <- interaction_input
CellChatDB$complex <- complex_input
CellChatDB$cofactor <- cofactor_input
CellChatDB$geneInfo <- geneInfo



#use all CellChatDB for cell-cell communication analysis
CellChatDB.use <- CellChatDB # simply use the default CellChatD

cellchat_Treat@DB <- CellChatDB.use # set the used database in the
object

cellchat_Treat <- subsetData(cellchat_Treat) # subset the expression
data of signaling genes for saving computation cost
future::plan("multiprocess", workers = 4)

cellchat_Treat <- identifyOverExpressedGenes(cellchat_Treat)
cellchat_Treat <- identifyOverExpressedInteractions(cellchat_Treat)
cellchat_Treat <- projectData(cellchat_Treat, PPI.human)

#cellchat_Treat1
cellchat_Treat <- computeCommunProb(cellchat_Treat,population.size =
TRUE)
```

```
cellchat_Treat <- computeCommunProbPathway(cellchat_Treat)
cellchat_Treat <- aggregateNet(cellchat_Treat)

# Compute the network centrality scores
cellchat_Treat    <-    netAnalysis_computeCentrality(cellchat_Treat,
slot.name = "netP") # the slot 'netP' means the inferred intercellular
communication network of signaling pathways

#cellchat_Treat1:cellchat_Treat                              <-
computeCommunProb(cellchat_Treat,population.size = TRUE)
saveRDS(cellchat_Treat,file="F:/scRNA/JCML/analysis3/20
2/celltype/Cellchat/cellchat_Treat1.RDS")
cellchat_Treat      <-      readRDS(file="F:/scRNA/JCML/analysis3/20
2/celltype/Cellchat/cellchat_Treat1.RDS")




#三。compare
library(igraph)

#Comparison analysis of multiple datasets using CellChat

cellchat_Naive      <-      readRDS(file="F:/scRNA/JCML/analysis3/20
2/celltype/Cellchat/cellchat_Naive1.RDS")
cellchat_Treat      <-      readRDS(file="F:/scRNA/JCML/analysis3/20
2/celltype/Cellchat/cellchat_Treat1.RDS")


levels(cellchat_Treat@idents)
levels(cellchat_Naive@idents)
object.list <- list(Naive = cellchat_Naive, Treat = cellchat_Treat)
cellchat <- mergeCellChat(object.list, add.names = names(object.list))
# Define the cell labels to lift up
cellchat@idents <- factor(cellchat@idents,level=c("Cancer_cell",

"CD4_T","Treg_FOXP3","CD8_T","NKT","DNT",

"B_cell","Plasma_cell","Cycling_cell",

"TAM","Neutrophil","Mast_cell",

"Fibroblast","Endotheliocyte"))
```

```
group.new = levels(cellchat@idents)
cellchat_Naive <- liftCellChat(cellchat_Naive, group.new)
cellchat_Treat <- liftCellChat(cellchat_Treat, group.new)
levels(cellchat_Treat@idents)
levels(cellchat_Naive@idents)

object.list <- list(Naive = cellchat_Naive, Treat = cellchat_Treat)
cellchat <- mergeCellChat(object.list, add.names = names(object.list))
#> Merge the following slots: 'data.signaling','net', 'netP','meta',
'idents', 'var.features' , 'DB', and 'LR'.
cellchat

#cellchat_Naive1\cellchat_Treat1
saveRDS(cellchat,file="F:/scRNA/JCML/analysis3/20
2/celltype/Cellchat/cellchat1_merge.RDS")
cellchat <- readRDS(file="F:/scRNA/JCML/analysis3/20
2/celltype/Cellchat/cellchat1_merge.RDS")


#Part I: Predict general principles of cell-cell communication

#Compare the total number of interactions and interaction strength
gg1 <- compareInteractions(cellchat, show.legend = F, group = c(1,2))
gg2 <- compareInteractions(cellchat, show.legend = F, group = c(1,2),
measure = "weight")
gg1 + gg2


#Differential number of interactions or interaction strength among
different cell populations
#The differential number of interactions or interaction strength in the
cell-cell communication network between two datasets can be visualized
using circle plot, where red (or blue) colored edges represent increased
(or decreased) signaling in the second dataset compared to the first
one.

#treat vs naive
par(mfrow = c(1,2), xpd=TRUE)
netVisual_diffInteraction(cellchat, weight.scale = T,comparison =
c("Naive","Treat"))
netVisual_diffInteraction(cellchat, weight.scale = T, measure =
"weight",comparison = c("Naive","Treat"))
```

#We can also show differential number of interactions or interaction strength in a greater details using a heatmap. The top colored bar plot represents the sum of column of values displayed in the heatmap (incoming signaling). The right colored bar plot represents the sum of row of values (outgoing signaling). In the colorbar, red (or blue) represents increased (or decreased) signaling in the second dataset compared to the first one.
#treat vs naive
gg1 <- netVisual_heatmap(cellchat,comparison = c("Naive","Treat"))
#> Do heatmap based on a merged object
gg2 <- netVisual_heatmap(cellchat, measure = "weight",comparison = c("Naive","Treat"))
#> Do heatmap based on a merged object
gg1 + gg2

#The differential network analysis only works for pairwise datasets.
#If there are more datasets for comparison, we can directly show the number of interactions or interaction strength between any two cell populations in each dataset.

#To better control the node size and edge weights of the inferred networks across different datasets, we compute the maximum number of cells per cell group and the maximum number of interactions (or interaction weights) across all datasets.
weight.max <- getMaxWeight(object.list, attribute = c("idents","count"))
par(mfrow = c(1,2), xpd=TRUE)
for (i in 1:length(object.list)) {
  netVisual_circle(object.list[[i]]@net$count, weight.scale = T, label.edge= F, edge.weight.max = weight.max[2], edge.width.max = 12, title.name = paste0("Number of interactions - ", names(object.list)[i]))
}

weight.max <- getMaxWeight(object.list, attribute = c("idents","weight"))
par(mfrow = c(1,2), xpd=TRUE)
for (i in 1:length(object.list)) {
  netVisual_circle(object.list[[i]]@net$weight, weight.scale = T, label.edge= F, edge.weight.max = weight.max[2], edge.width.max = 12, title.name = paste0("Interaction strength - ", names(object.list)[i]))
}

#Compare the major sources and targets in 2D space
num.link <- sapply(object.list, function(x) {rowSums(x@net$count) +

```
colSums(x@net$count)-diag(x@net$count)})
weight.MinMax <- c(min(num.link), max(num.link)) # control the dot size in the different datasets
gg <- list()
for (i in 1:length(object.list)) {
  gg[[i]] <- netAnalysis_signalingRole_scatter(object.list[[i]], title = names(object.list)[i], weight.MinMax = weight.MinMax)
}
#> Signaling role analysis on the aggregated cell-cell communication network from all signaling pathways
patchwork::wrap_plots(plots = gg)
```

```
#This bar graph can be plotted in a stacked mode or not. Significant signaling pathways were ranked based on differences in the overall information flow within the inferred networks between NL and LS skin. The top signaling pathways colored red are enriched in NL skin, and these colored green were enriched in the LS skin.
#all
gg1 <- rankNet(cellchat, mode = "comparison", stacked = T, do.stat = TRUE)
gg2 <- rankNet(cellchat, mode = "comparison", stacked = F, do.stat = TRUE)
gg1 + gg2
```
