# Supplementary material for: Single-cell analysis reveals cellular reprogramming in advanced colon cancer following FOLFOX-bevacizumab treatment
Source: Front Oncol. 2023 Jul 28;13:1219642. doi: 10.3389/fonc.2023.1219642 (PMC10421721; doi:10.3389/fonc.2023.1219642)
Supplement: Supplementary file 3 [file DataSheet_1.zip › PDF/FigureS6.pdf]

```
library(Seurat)
library(dplyr)
library(patchwork)
library(tidyverse)
library(stringr)
library(metap)
library(ggpubr)
rm(list=ls())

getwd()
setwd("F:/scRNA/JCML/GSE178318/analysis2/")

JCML.data <- Read10X(data.dir = "F:/scRNA/JCML/GSE178318/raw_data/")
JCML   <-   CreateSeuratObject(counts   =   JCML.data,min.cells   =   10,
min.features = 200, project = "JCML")
View(JCML@meta.data)
JCML@meta.data$Cell.Barcode <- rownames(JCML@meta.data)

JCML@meta.data$orig.ident                                          <-
sapply(strsplit(as.character(JCML@meta.data$Cell.Barcode), '_'), "[", 2)
JCML@meta.data$site                                                <-
sapply(strsplit(as.character(JCML@meta.data$Cell.Barcode), '_'), "[", 3)
View(JCML@meta.data)

table(JCML$site)

JCML_CRC <- subset(JCML, subset=site=="CRC")
#saveRDS(JCML_CRC,file="F:/scRNA/JCML/GSE178318/analysis2/JCML_CRC.RDS
")
JCML_CRC                                                           <-
readRDS(file="F:/scRNA/JCML/GSE178318/analysis2/JCML_CRC.RDS")
table(JCML_CRC$orig.ident)

JCML_CRC[["percent.mt"]]  <-  PercentageFeatureSet(JCML_CRC,  pattern  =
"^MT-")
VlnPlot(JCML_CRC,    features    =    c("nFeature_RNA",    "nCount_RNA",
"percent.mt"), ncol = 3)
JCML_CRC <- subset(JCML_CRC, subset = nFeature_RNA > 200 & nFeature_RNA
< 6000 & percent.mt < 25)
JCML_CRC


# split the dataset into a list of two seurat objects (Naive and Treat)
JCML.list <- SplitObject(JCML_CRC, split.by = "orig.ident")
```

```
# normalize and identify variable features for each dataset independently
JCML.list <- lapply(X = JCML.list, FUN = function(x) {
  x <- NormalizeData(x)
  x <- FindVariableFeatures(x, selection.method = "vst", nfeatures = 2000)
})

# select features that are repeatedly variable across datasets for integration
features <- SelectIntegrationFeatures(object.list = JCML.list)

### Integration ----
JCML.anchors <- FindIntegrationAnchors(object.list = JCML.list, anchor.features = features)

# this command creates an 'integrated' data assay
JCML.combined <- IntegrateData(anchorset = JCML.anchors)
JCML.combined
#saveRDS(JCML.combined,file="F:/scRNA/JCML/GSE178318/analysis2/JCML_combined.RDS")
JCML.combined <- readRDS(file="F:/scRNA/JCML/GSE178318/analysis2/JCML_combined.RDS")


# specify that we will perform downstream analysis on the corrected data note that the original
# unmodified data sNKTl resides in the 'RNA' assay
DefaultAssay(JCML.combined) <- "integrated"

# Run the standard workflow for visualization and clustering
JCML.combined <- ScaleData(JCML.combined, verbose = FALSE)
JCML.combined <- RunPCA(JCML.combined,verbose = FALSE)
ElbowPlot(JCML.combined)

JCML.combined <- RunUMAP(JCML.combined, reduction = "pca", dims = 1:20)
JCML.combined <- RunTSNE(JCML.combined, reduction = "pca", dims = 1:20)
JCML.combined <- FindNeighbors(JCML.combined, reduction = "pca", dims = 1:20)
#saveRDS(JCML.combined,file="F:/scRNA/JCML/GSE178318/analysis2/JCML_combined_20.RDS")
JCML.combined <- readRDS(file="F:/scRNA/JCML/GSE178318/analysis2/JCML_combined_20.RDS")
```

```
JCML.combined <- FindClusters(JCML.combined, resolution = 0.8)
#saveRDS(JCML.combined,file="F:/scRNA/JCML/GSE178318/analysis2/JCML_combined_20_0.8.RDS")
JCML.combined <- readRDS(file="F:/scRNA/JCML/GSE178318/analysis2/JCML_combined_20_0.8.RDS")

table(Idents(JCML.combined))
# Visualization
p1 <- DimPlot(JCML.combined, reduction = "umap", group.by = "orig.ident")+theme(panel.background = element_blank(),panel.grid.major = element_blank(),panel.border = element_rect(colour="black",fill=NA))
p2 <- DimPlot(JCML.combined, reduction = "umap", label = TRUE, repel = TRUE)+theme(panel.background = element_blank(),panel.grid.major = element_blank(),panel.border = element_rect(colour="black",fill=NA))
p1 + p2

p3 <- DimPlot(JCML.combined, reduction = "tsne", group.by = "orig.ident")+theme(panel.background = element_blank(),panel.grid.major = element_blank(),panel.border = element_rect(colour="black",fill=NA))
p4 <- DimPlot(JCML.combined, reduction = "tsne",label = TRUE, repel = TRUE,pt.size=1)+theme(panel.background = element_blank(),panel.grid.major = element_blank(),panel.border = element_rect(colour="black",fill=NA))
p3 + p4


#To visualize the two conditions side-by-side, we can use the split.by argument to show each condition colored by cluster.

DimPlot(JCML.combined, reduction = "umap", split.by = "orig.ident",label = TRUE)+theme(panel.background = element_blank(),panel.grid.major = element_blank(),panel.border = element_rect(colour="black",fill=NA))
DimPlot(JCML.combined, reduction = "tsne", split.by = "orig.ident",label = TRUE)+theme(panel.background = element_blank(),panel.grid.major = element_blank(),panel.border = element_rect(colour="black",fill=NA))

DimPlot(JCML_CRC, reduction = "umap", label = TRUE)+theme(panel.background = element_blank(),panel.grid.major = element_blank(),panel.border = element_rect(colour="black",fill=NA))
```

```
#We can explore these marker genes for each cluster and use them to annotate our clusters as specific cell types.
DefaultAssay(JCML.combined) <- "RNA"

VlnPlot(JCML.combined, features = c("EPCAM","KRT19","KRT18",

"PTPRC","CD14","CD68","LYZ","KRT18",

"CD3D","GNLY","KLRF1","KLRD1","MS4A1","CD79A","MZB1",
                                    "CSF3R","S100A8","G0S2",
                                    "KIT","GATA2","TPSAB1",
                                    "DCN","LUM","THY1",
                                    "VWF","RAMP2","CD34",
                                    "MKI67"),pt.size = 0)

markers <- FindAllMarkers(JCML.combined,only.pos = TRUE, min.pct = 0.25, logfc.threshold =0.25)
write.csv(markers,file="F:/scRNA/JCML/GSE178318/analysis2/20
0.8/markers.csv")
write.csv(markers,file="F:/scRNA/JCML/GSE178318/analysis2/20
0.8/celltype/markers.csv")


top10 <- markers %>% group_by(cluster) %>% top_n(n = 10, wt = avg_log2FC)
DefaultAssay(JCML.combined) <- "integrated"
DoHeatmap(JCML.combined, features = top10$gene)
DoHeatmap(JCML.combined, features = top10$gene) + NoLegend()

JCML.combined <-
readRDS(file="F:/scRNA/JCML/GSE178318/analysis2/JCML_combined_20_0.8.RDS")
DimPlot(JCML.combined, reduction = "tsne",label = TRUE, repel = TRUE,pt.size=1)+theme(panel.background = element_blank(),panel.grid.major = element_blank(),panel.border = element_rect(colour="black",fill=NA))
DimPlot(JCML.combined, reduction = "umap",label = TRUE, repel = TRUE,pt.size=1)+theme(panel.background = element_blank(),panel.grid.major = element_blank(),panel.border = element_rect(colour="black",fill=NA))

JCML.combined@meta.data
```

```
Myeloid_cell=c(6,8,15,17,18,20)
Cancer_cell=c(4,10,13,14)
Fibroblast=c(16,21)
T_cell=c(0,1,2,5,9,11,12)
B_cell=c(3,7,22)
Endotheliocyte=c(19)


current.cluster.ids                                                    <-
c(Myeloid_cell,Cancer_cell,Fibroblast,T_cell,B_cell,Endotheliocyte)
new.cluster.ids <- c(rep("Myeloid_cell",length(Myeloid_cell)),
                     rep("Cancer_cell",length(Cancer_cell)),
                     rep("Fibroblast",length(Fibroblast)),
                     rep("T_cell",length(T_cell)),
                     rep("B_cell",length(B_cell)),
                     rep("Endotheliocyte",length(Endotheliocyte)))

JCML.combined@meta.data$Cluster       <-       plyr::mapvalues(x       =
as.integer(as.character(JCML.combined@meta.data$seurat_clusters)),
from = current.cluster.ids, to = new.cluster.ids)
head(JCML.combined@meta.data)
table(JCML.combined@meta.data$Cluster)


JCML.combined$Cluster                                                  <-
factor(JCML.combined$Cluster,level=c("Cancer_cell",

"T_cell","B_cell","Myeloid_cell",

"Fibroblast","Endotheliocyte"))
Idents(JCML.combined) <- "Cluster"
table(Idents(JCML.combined))


orig.ident <- c("COL07","COL12","COL15","COL16","COL17","COL18")
Treatment1<- c("Naive","Naive","Treat","Naive","Treat","Treat")
Treatment2 <- c("Naive","Naive","PC_CAP","Naive","PC_CAP","PC_Bev")

JCML.combined@meta.data$Treatment1      <-      plyr::mapvalues(x      =
JCML.combined@meta.data$orig.ident, from = orig.ident, to = Treatment1)
JCML.combined@meta.data$Treatment2      <-      plyr::mapvalues(x      =
JCML.combined@meta.data$orig.ident, from = orig.ident, to = Treatment2)

head(JCML.combined@meta.data)
```

```
table(JCML.combined@meta.data$Treatment1)
table(JCML.combined@meta.data$Treatment2)
table(Idents(JCML.combined))

JCML.combined$orig.ident <- factor(JCML.combined$orig.ident, levels = c("COL07","COL12","COL16",

"COL18","COL15","COL17"))

#saveRDS(JCML.combined,file="F:/scRNA/JCML/GSE178318/analysis2/JCML_combined_20_0.8_celltype_all.RDS")
JCML.combined <- readRDS(file="F:/scRNA/JCML/GSE178318/analysis2/JCML_combined_20_0.8_celltype_all.RDS")

JCML.combined <- subset(JCML.combined,subset=Treatment2=="PC_CAP",invert=TRUE)
table(JCML.combined@meta.data$Treatment2)
#saveRDS(JCML.combined,file="F:/scRNA/JCML/GSE178318/analysis2/JCML_combined_20_0.8_celltype_used.RDS")
JCML.combined <- readRDS(file="F:/scRNA/JCML/GSE178318/analysis2/JCML_combined_20_0.8_celltype_used.RDS")

# Visualization
p1 <- DimPlot(JCML.combined, reduction = "umap", group.by = "orig.ident")+theme(panel.background = element_blank(),panel.grid.major = element_blank(),panel.border = element_rect(colour="black",fill=NA))
p2 <- DimPlot(JCML.combined, reduction = "umap", repel = TRUE)+theme(panel.background = element_blank(),panel.grid.major = element_blank(),panel.border = element_rect(colour="black",fill=NA))
p1 + p2

p3 <- DimPlot(JCML.combined, reduction = "tsne", group.by = "orig.ident")+theme(panel.background = element_blank(),panel.grid.major = element_blank(),panel.border = element_rect(colour="black",fill=NA))
p4 <- DimPlot(JCML.combined, reduction = "tsne",repel = TRUE,pt.size=1)+theme(panel.background = element_blank(),panel.grid.major = element_blank(),panel.border = element_rect(colour="black",fill=NA))
p3 + p4
```

```
DefaultAssay(JCML.combined) <- "RNA"

markers <- FindAllMarkers(JCML.combined,only.pos = TRUE, min.pct = 0.25,
logfc.threshold =0.25)

top10 <- markers %>% group_by(cluster) %>% top_n(n = 10, wt = avg_log2FC)
DefaultAssay(JCML.combined) <- "integrated"
DoHeatmap(JCML.combined, features = top10$gene)
DoHeatmap(JCML.combined, features = top10$gene) + NoLegend()




###Cancer cell

library(ggplot2)
library(cowplot)
library(Seurat)
library(dplyr)
library(patchwork)
library(ggpubr)
library(reshape2)
theme_set(theme_cowplot())
rm(list=ls())

JCML.combined                                                        <-
readRDS(file="F:/scRNA/JCML/GSE178318/analysis2/JCML_combined_20_0.8_c
elltype_used.RDS")

tumor <- subset(JCML.combined,idents = "Cancer_cell")

tumor <- RunPCA(tumor)
ElbowPlot(tumor)

tumor <- RunUMAP(tumor, reduction = "pca", dims = 1:15)
tumor <- RunTSNE(tumor, reduction = "pca", dims = 1:15)
tumor <- FindNeighbors(tumor, reduction = "pca", dims = 1:15)
#saveRDS(tumor,file       =       "F:/scRNA/JCML/GSE178318/analysis2/20
0.8/celltype/cancer_cell/Cancer_cell_15.RDS")
tumor    <-    readRDS(file    =    "F:/scRNA/JCML/GSE178318/analysis2/20
0.8/celltype/cancer_cell/Cancer_cell_15.RDS")

DefaultAssay(tumor) <- "integrated"
```

```
tumor <- FindClusters(tumor, resolution = 0.2)

DimPlot(tumor, reduction = "umap",label = TRUE)+theme(panel.background = element_blank(),panel.grid.major = element_blank(),panel.border = element_rect(colour="black",fill=NA))

# Visualization
p1 <- DimPlot(tumor, reduction = "umap", group.by = "Treatment1")+theme(panel.background = element_blank(),panel.grid.major = element_blank(),panel.border = element_rect(colour="black",fill=NA))
p2 <- DimPlot(tumor, reduction = "umap",label = TRUE, repel = TRUE)+theme(panel.background = element_blank(),panel.grid.major = element_blank(),panel.border = element_rect(colour="black",fill=NA))
p1 + p2

p3 <- DimPlot(tumor, reduction = "tsne", group.by = "Treatment1")+theme(panel.background = element_blank(),panel.grid.major = element_blank(),panel.border = element_rect(colour="black",fill=NA))
p4 <- DimPlot(tumor, reduction = "tsne",label = TRUE,repel = TRUE,pt.size=1)+theme(panel.background = element_blank(),panel.grid.major = element_blank(),panel.border = element_rect(colour="black",fill=NA))
p3 + p4



#15 0.2
tumor <- readRDS(file = "F:/scRNA/JCML/GSE178318/analysis2/200.8/celltype/cancer_cell/Cancer_cell_15_0.2.RDS")
table(Idents(tumor))

Sensitive=c(0,2,3)
Non_sensitive=c(1)

current.cluster.ids <- c(Sensitive,Non_sensitive)
new.cluster.ids <- c(rep("Sensitive",length(Sensitive)),
                     rep("Non_sensitive",length(Non_sensitive)))

tumor@meta.data$Celltype <- plyr::mapvalues(x = as.integer(as.character(tumor@meta.data$seurat_clusters)), from = current.cluster.ids, to = new.cluster.ids)
```

```
table(tumor@meta.data$Celltype)

tumor$Celltype <-
factor(tumor$Celltype ,level=c("Sensitive","Non_sensitive"))
Idents(tumor)<-"Celltype"
table(Idents(tumor))

saveRDS(tumor,file="F:/scRNA/JCML/GSE178318/analysis2/20
0.8/celltype/cancer_cell/Cancer_cell_15_0.2_celltype.RDS")
tumor <- readRDS(file="F:/scRNA/JCML/GSE178318/analysis2/20
0.8/celltype/cancer_cell/Cancer_cell_15_0.2_celltype.RDS")
View(tumor@meta.data)
DimPlot(tumor, reduction = "umap",label = FALSE, split.by =
"Treatment1")+theme(panel.background =
element_blank(),panel.grid.major = element_blank(),panel.border =
element_rect(colour="black",fill=NA))
DimPlot(tumor, reduction = "tsne",label = FALSE, split.by =
"Treatment1")+theme(panel.background =
element_blank(),panel.grid.major = element_blank(),panel.border =
element_rect(colour="black",fill=NA))


library(Seurat)
str(cc.genes)

tumor <- readRDS(file = "F:/scRNA/JCML/GSE178318/analysis2/20
0.8/celltype/cancer_cell/Cancer_cell_15_0.2.RDS")
DefaultAssay(tumor) <- "RNA"

tumor <- CellCycleScoring(tumor,
                          s.features = cc.genes$s.genes,
                          g2m.features = cc.genes$g2m.genes,
                          seed = 1,
                          set.ident =TRUE)


head(x = tumor@meta.data)

plot(tumor$S.Score,tumor$G2M.Score,
     col=factor(tumor$Phase),
     main="CellCycleScoring")
legend("topright",inset=.05,
       title = "cell cycle",
       c("G1","S","G2M"), pch = c(1),col=c("black","green","red"))
```

```
DefaultAssay(tumor) <- "integrated"

saveRDS(tumor,file="F:/scRNA/JCML/GSE178318/analysis2/20
0.8/celltype/cancer_cell/Cancer_cell_15_0.2_cellcycle.RDS")

DimPlot(tumor, reduction = "tsne",group.by = "Phase")+
  theme(panel.background    =    element_blank(),panel.grid.major    =
element_blank(),panel.border = element_rect(colour="black",fill=NA))

DimPlot(tumor,   reduction   =   "umap",group.by   =   "Phase",split.by   =
"Treatment1")+
  theme(panel.background    =    element_blank(),panel.grid.major    =
element_blank(),panel.border = element_rect(colour="black",fill=NA))

DimPlot(tumor, reduction = "umap",
        group.by = "Phase",
        shape.by = "Treatment1",
        pt.size =2)

DimPlot(tumor, reduction = "pca",
        group.by = "Treatment1",
        shape.by = "Phase",
        pt.size =2)

DimPlot(tumor, reduction = "tsne",
        group.by = "Treatment1",
        shape.by = "Phase")

DimPlot(tumor, reduction = "umap",
        group.by = "Treatment1",
        shape.by = "Phase",
        pt.size =2)

#cell component
#proportion
tumor    <-    readRDS(file    =    "F:/scRNA/JCML/GSE178318/analysis2/20
0.8/celltype/cancer_cell/Cancer_cell_15_0.2_cellcycle.RDS")
#Treatment1
table(tumor$Treatment1)
table(Idents(tumor))
prop.table(table(Idents(tumor)))
table(Idents(tumor), tumor$Treatment1)
```

```
prop.table(table(Idents(tumor), tumor$Treatment1), margin = 2)
tumor_p<-as.data.frame(prop.table(table(Idents(tumor),
tumor@meta.data[,"Treatment1"]), margin = 2))
#纵向
ggplot(tumor_p,aes(x=tumor_p[,2],y=tumor_p[,3],fill=tumor_p[,1]))+
  geom_bar(position = 'stack',stat="identity")+
  labs(x="Treatment1",y="Cell proportion")+
  theme(panel.background=element_rect(fill='transparent',
color='black'),panel.border =element_rect(fill=NA,color='black'),
        legend.key=element_rect(fill='transparent',
color='transparent'),axis.text = element_text(color="black"))+
  scale_y_continuous(expand=c(0.001,0.001))+
  guides(fill = guide_legend(keywidth = 1, keyheight = 1,ncol=1,title =
'Cell types'))



#GSEA
library(presto)
library(msigdbr)
library(fgsea)
library(dplyr)
library(ggplot2)
library(tibble)

scRNAsub <- readRDS(file = "F:/scRNA/JCML/GSE178318/analysis2/20
0.8/celltype/cancer_cell/Cancer_cell_15_0.2_celltype.RDS")
scRNAsub <- tumor
table(Idents(scRNAsub))
DefaultAssay(scRNAsub) <- "RNA"

scRNAsub.markers <- FindAllMarkers(scRNAsub, only.pos = TRUE, min.pct =
0.25, logfc.threshold = 0.25)

scRNAsub.genes <- wilcoxauc(scRNAsub, 'Celltype')
head(scRNAsub.genes)
dplyr::count(scRNAsub.genes, group)

scRNAsub.genes %>%
  dplyr::filter(group == "Sensitive") %>%
  arrange(desc(logFC), desc(auc)) %>%
  head(n = 10)       #进行降序排序

Sensitive.genes<-  scRNAsub.genes  %>%  dplyr::filter(group  ==
```

```
"Sensitive") %>% arrange(desc(auc)) %>% dplyr::select(feature, auc)
ranks<- deframe(Sensitive.genes)
head(ranks)


msigdbr_species()
m_df<- msigdbr(species = "Homo sapiens", category = "H")

head(m_df)

fgsea_sets<- m_df %>% split(x = .$gene_symbol, f = .$gs_name)

summary(fgsea_sets)

fgseaRes<- fgsea(fgsea_sets, stats = ranks, nperm = 1000)
fgseaResTidy <- fgseaRes %>%  as_tibble() %>% arrange(desc(NES))
fgseaResTidy  %>%  dplyr::select(-leadingEdge,  -ES,  -nMoreExtreme)  %>%
arrange(padj) %>% head()
View(fgseaResTidy)

ggplot(fgseaResTidy   %>%   filter(pval   <   0.05)   %>%   head(n=   50),
aes(reorder(pathway, NES), NES)) +
  geom_col(aes(fill= NES < 0)) +
  coord_flip() +
  labs(x="Pathway", y="Normalized Enrichment Score",
       title="Hallmark pathways NES from GSEA") +
  theme_minimal() ####以 7.5 进行绘图填色
ggplot(fgseaResTidy   %>%   filter(padj   <   0.01)   %>%   head(n=   50),
aes(reorder(pathway, NES), NES)) +
  geom_col(aes(fill= NES < 0)) +
  coord_flip() +
  labs(x="Pathway", y="Normalized Enrichment Score",
       title="Hallmark pathways NES from GSEA") +
  theme_minimal() ####以 7.5 进行绘图填色

ggplot(fgseaResTidy   %>%   filter(pval   <   0.05)   %>%   head(n=   38),
aes(reorder(pathway, NES), NES)) +
  geom_col(aes(fill= NES < 0)) +
  coord_flip() +
  labs(x="Pathway", y="Normalized Enrichment Score",
       title="Hallmark pathways NES from GSEA") +
  theme_minimal() ####以 7.5 进行绘图填色

ggplot(fgseaResTidy %>% filter(pval < 0.01), aes(reorder(pathway, NES),
```

```
-log10(pval))) +
  geom_col(aes(fill= NES < 0)) +
  coord_flip() +
  labs(x="Pathway", y="-log10(pval)",
       title="Hallmark pathways NES from GSEA") +
  theme_minimal() ####以 7.5 进行绘图填色

Sensitive <- fgseaResTidy %>% filter(pval < 0.01)
View(Sensitive)
pathway <- Sensitive$pathway
pval<-Sensitive$pval
padj<-Sensitive$padj
ES <-Sensitive$ES
NES<-Sensitive$NES
LeadingEdge<-Sensitive$leadingEdge
size<-Sensitive$size

df<-data.frame(pathway,pval,padj,ES,NES,size)
#2
write.csv(df,file        =        "F:/scRNA/JCML/GSE178318/analysis2/20
0.8/celltype/cancer_cell/Sensitive_VS_NonSensitive_p0.01.csv",row.name
s = TRUE)

library(ggplot2)
#used
DF       <-        read.csv(file="F:/scRNA/JCML/GSE178318/analysis2/20
0.8/celltype/cancer_cell/Sensitive_VS_NonSensitive_p0.01_used.csv")

ggplot(DF, aes(reorder(pathway, NES), -log10(pval))) +
  geom_col(aes(fill= NES < 0)) +
  coord_flip() +
  labs(x="Pathway", y="-log10(pval)") +
  theme_minimal() ####以 7.5 进行绘图填色


####Myeloid cell

library(ggplot2)
library(cowplot)
library(Seurat)
library(dplyr)
library(patchwork)
library(ggpubr)
library(reshape2)
```

```
rm(list=ls())

JCML.combined <-
readRDS(file="F:/scRNA/JCML/GSE178318/analysis2/JCML_combined_20_0.8_celltype_used.RDS")
table(JCML.combined@meta.data$Treatment1)
Myeloid_cell <- subset(JCML.combined,idents="Myeloid_cell")
DefaultAssay(Myeloid_cell) <- "integrated"
Myeloid_cell <- RunPCA(Myeloid_cell,verbose = FALSE)
ElbowPlot(Myeloid_cell)

Myeloid_cell <- RunUMAP(Myeloid_cell, reduction = "pca", dims = 1:15)
Myeloid_cell <- RunTSNE(Myeloid_cell, reduction = "pca", dims = 1:15)
Myeloid_cell <- FindNeighbors(Myeloid_cell, reduction = "pca", dims =
1:15)
#saveRDS(Myeloid_cell,file = "F:/scRNA/JCML/GSE178318/analysis2/20
0.8/celltype/Myeloid/Myeloid_cell_15.RDS")
Myeloid_cell <- readRDS(file = "F:/scRNA/JCML/GSE178318/analysis2/20
0.8/celltype/Myeloid/Myeloid_cell_15.RDS")

Myeloid_cell <- FindClusters(Myeloid_cell, resolution = 0.1)

p3 <- DimPlot(Myeloid_cell, reduction = "tsne", group.by =
"Treatment1")+theme(panel.background =
element_blank(),panel.grid.major = element_blank(),panel.border =
element_rect(colour="black",fill=NA))
p4 <- DimPlot(Myeloid_cell, reduction = "tsne",label = TRUE, repel =
TRUE,pt.size=1)+theme(panel.background =
element_blank(),panel.grid.major = element_blank(),panel.border =
element_rect(colour="black",fill=NA))
p4 + p3

p3 <- DimPlot(Myeloid_cell, reduction = "umap", group.by =
"Treatment1")+theme(panel.background =
element_blank(),panel.grid.major = element_blank(),panel.border =
element_rect(colour="black",fill=NA))
p4 <- DimPlot(Myeloid_cell, reduction = "umap",label = TRUE, repel =
TRUE,pt.size=1)+theme(panel.background =
element_blank(),panel.grid.major = element_blank(),panel.border =
element_rect(colour="black",fill=NA))
p4 + p3

table(Idents(Myeloid_cell))
```

```
DefaultAssay(Myeloid_cell) <- "RNA"

Myeloid_cell.markers <- FindAllMarkers(Myeloid_cell, only.pos = TRUE, min.pct = 0.25, logfc.threshold =0.25)
write.csv(Myeloid_cell.markers,file="F:/scRNA/JCML/GSE178318/analysis2/20 0.8/celltype/Myeloid/Myeloid_marker.csv")

top10 <- Myeloid_cell.markers %>% group_by(cluster) %>% top_n(n = 10, wt = avg_log2FC)
DefaultAssay(Myeloid_cell) <- "integrated"
DoHeatmap(Myeloid_cell, features = top10$gene)

DefaultAssay(Myeloid_cell) <- "RNA"


#DC:"CD1A","CD1C","FCER1A","CLEC9A","CCR7"
#pDC: IGJ,"LILRA4", "CLEC4C/DLEC/BDCA2",IL3RA/CD123,NRP1/BDCA4
#Langerhans cell:FCER1A
#migratory DCs:CCR7,AXL
#CD83 is a specific marker of mature DCs;CR2/CD21,CLU/clusterin for follicular DCs
#classical DC1 marker gene THBD(CD141)
#DC2 cells (CD1C and CLEC10A),
#DC3 cells (CCL19,LAMP3, and CCR7) could represent a Myeloid_cell-specific DC subpopulation, because they were also detected in our reanalysis of melanoma , head andneck head cancer,but were not found in PBMCs
VlnPlot(Myeloid_cell, features = c("CD1A","CD1C","FCER1A","CLEC9A","CCR7",

"IGJ","LILRA4","CLEC4C","DLEC","BDCA2","IL3RA","NRP1",

"AXL","THBD","CLEC10A","CD83","CR2","CLU","CCL19","LAMP3","IDO1"))
VlnPlot(Myeloid_cell, features = c("IFNB1"))


#neutrophils characterized by S100A8, S100A9, and GOS2
VlnPlot(Myeloid_cell, features = c("CSF3R","S100A8", "S100A9","GOS2","MPO","CD15","FUT4","CD32","CD66b","CEACAM8","SELL","ELANE","BPI"))

#MAST CELL
VlnPlot(Myeloid_cell, features =
```

```
c("KIT","MS4A2","GATA2","IL2RA","PTPRC","FCER2","TPSAB1","FCER1A"))



#15 0.1
Myeloid_cell <- readRDS(file = "F:/scRNA/JCML/GSE178318/analysis2/20
0.8/celltype/Myeloid/Myeloid_cell_15_0.1.RDS")

TAM_M1=c(1)
TAM_M1M2=c(0)
pDC=c(3)
Mast_cell=c(2)
DC_LAMP3=c(4)
DC_CLEC9A=c(5)

current.cluster.ids <-
c(TAM_M1,TAM_M1M2,pDC,Mast_cell,DC_LAMP3,DC_CLEC9A)
new.cluster.ids <- c(rep("TAM_M1",length(TAM_M1)),
                     rep("TAM_M1M2",length(TAM_M1M2)),
                     rep("pDC",length(pDC)),
                     rep("Mast_cell",length(Mast_cell)),
                     rep("DC_LAMP3",length(DC_LAMP3)),
                     rep("DC_CLEC9A",length(DC_CLEC9A)))

Myeloid_cell@meta.data$Celltype <- plyr::mapvalues(x =
as.integer(as.character(Myeloid_cell@meta.data$seurat_clusters)), from
= current.cluster.ids, to = new.cluster.ids)
head(Myeloid_cell@meta.data)
table(Myeloid_cell@meta.data$Celltype)
table(Idents(Myeloid_cell))

Myeloid_cell$Celltype <-
factor(Myeloid_cell$Celltype ,level=c("TAM_M1","TAM_M1M2","pDC","DC_LA
MP3","DC_CLEC9A","Mast_cell"))

Idents(Myeloid_cell)<-"Celltype"
table(Idents(Myeloid_cell))

#saveRDS(Myeloid_cell,file="F:/scRNA/JCML/GSE178318/analysis2/20
0.8/celltype/Myeloid/Myeloid_15_0.1_Celltype")
Myeloid_cell <- readRDS(file = "F:/scRNA/JCML/GSE178318/analysis2/20
0.8/celltype/Myeloid/Myeloid_15_0.1_Celltype")

p3 <- DimPlot(Myeloid_cell, reduction = "tsne", group.by =
```

```
"Treatment1")+theme(panel.background =
element_blank(),panel.grid.major = element_blank(),panel.border =
element_rect(colour="black",fill=NA))
p4 <- DimPlot(Myeloid_cell, reduction = "tsne", repel =
TRUE,pt.size=1)+theme(panel.background =
element_blank(),panel.grid.major = element_blank(),panel.border =
element_rect(colour="black",fill=NA))
p4 + p3

p3 <- DimPlot(Myeloid_cell, reduction = "umap", group.by =
"Treatment1")+theme(panel.background =
element_blank(),panel.grid.major = element_blank(),panel.border =
element_rect(colour="black",fill=NA))
p4 <- DimPlot(Myeloid_cell, reduction = "umap", repel =
TRUE,pt.size=1)+theme(panel.background =
element_blank(),panel.grid.major = element_blank(),panel.border =
element_rect(colour="black",fill=NA))
p4 + p3

#macrophage
library(Seurat)
?AddModuleScore
library(tidyverse)
library(Matrix)
library(cowplot)
library(readxl)
#Macrophage
Myeloid_cell <- readRDS(file = "F:/scRNA/JCML/GSE178318/analysis2/20
0.8/celltype/Myeloid/Myeloid_15_0.1_Celltype")
table(Idents(Myeloid_cell))

macrophage <- subset(Myeloid_cell,idents=c("TAM_M1","TAM_M1M2"))

macrophage <- subset(Myeloid_cell,idents=c(0,1))
macrophage <- subset(Myeloid_cell,idents=c(0))
table(Idents(macrophage))
DefaultAssay(macrophage) <- "RNA"

#M1
M1 <- readxl::read_xlsx("E:/single cell sequence/Score gene
sets/immunocyte/M1.xlsx")
#View(M1)

gene <- as.list(M1)
```

```
macrophage <- AddModuleScore(
  object = macrophage,
  features = gene,
  ctrl = 100,
  name = 'M1_Score')

#M2
M2 <- readxl::read_xlsx("E:/single cell sequence/Score gene sets/immunocyte/M2.xlsx")
#View(M2)
gene <- as.list(M2)
macrophage <- AddModuleScore(
  object = macrophage,
  features = gene,
  ctrl = 100,
  name = 'M2_Score')

#Pro_inflammatory
Pro_inflammatory <- readxl::read_xlsx("E:/single cell sequence/Score gene sets/immunocyte/Pro_inflammatory.xlsx")
#View(Pro_inflammatory)
gene <- as.list(Pro_inflammatory)
macrophage <- AddModuleScore(
  object = macrophage,
  features = gene,
  ctrl = 100,
  name = 'Pro_inflammatory_Score')


#Anti_inflammatory
Anti_inflammatory <- readxl::read_xlsx("E:/single cell sequence/Score gene sets/immunocyte/Anti_inflammatory.xlsx")
#View(Anti_inflammatory)

gene <- as.list(Anti_inflammatory)
macrophage <- AddModuleScore(
  object = macrophage,
  features = gene,
  ctrl = 100,
  name = 'Anti_inflammatory_Score')

#KEGG_antigen_process_present_geneset
APP <- read.csv("E:/single cell sequence/Score gene sets/immunocyte/KEGG_antigen_process_present.csv")
```

```r
#View(APP)
gene <- as.list(APP)
macrophage <- AddModuleScore(
  object = macrophage,
  features = gene,
  ctrl = 100,
  name = 'APP_score')


#co-stimulatory:OX40L/TNFSF4,4-1BBL/TNFSF9.CD40LG/CD154,"CD27","CD40","CD70"
Costimulatory <- read.csv("E:/single cell sequence/Score gene sets/immunocyte/Costimulatory ligand used.csv")
#View(Costimulatory)
#"CD80","CD86","ICOSLG","TNFSF4","TNFSF9","TNFSF14"
gene <- as.list(Costimulatory)
macrophage <- AddModuleScore(
  object = macrophage,
  features = gene,
  ctrl = 100,
  name = 'Costimulatory_score')

MHC_I <- readxl::read_xlsx("E:/single cell sequence/Score gene sets/HLA_classI.xlsx",col_names = FALSE)
#View(MHC_I)
gene <- as.list(MHC_I)
macrophage <- AddModuleScore(
  object = macrophage,
  features = gene,
  ctrl =100,
  name = 'MHC_I',
  seed=1)

MHC_II <- readxl::read_xlsx("E:/single cell sequence/Score gene sets/HLA_classII.xlsx",col_names = FALSE)
#View(APP)
gene <- as.list(MHC_II)
macrophage <- AddModuleScore(
  object = macrophage,
  features = gene,
  ctrl =100,
  name = 'MHC_II',
  seed=1)
```

```
#IFNγ_response
IFNγ_response <- readxl::read_xlsx("E:/single cell sequence/Score gene sets/HALLMARK_INTERFERON_GAMMA_RESPONSE.xlsx",col_names = FALSE)
#View(IFNγ_response)

gene <- as.list(IFNγ_response)
macrophage <- AddModuleScore(
  object = macrophage,
  features = gene,
  ctrl =100,
  name = 'IFNγ_response',
  seed=1)



#HALLMARK_ANGIOGENESIS
Angiogenesis <- readxl::read_xlsx("E:/single cell sequence/Score gene sets/HALLMARK_ANGIOGENESIS.xlsx",col_names = FALSE)
#View(Angiogenesis)
gene <- as.list(Angiogenesis)
macrophage <- AddModuleScore(
  object = macrophage,
  features = gene,
  ctrl =100,
  name = 'Angiogenesis',
  seed=1)

colnames(macrophage@meta.data)
colnames(macrophage@meta.data)[14] <- 'M1_score'
colnames(macrophage@meta.data)[15] <- 'M2_score'
colnames(macrophage@meta.data)[16] <- 'Pro_inflammatory_score'
colnames(macrophage@meta.data)[17] <- 'Anti_inflammatory_score'
colnames(macrophage@meta.data)[18] <- 'APP_score'
colnames(macrophage@meta.data)[19] <- 'Costimulatory_score'
colnames(macrophage@meta.data)[20] <- 'MHC_I'
colnames(macrophage@meta.data)[21] <- 'MHC_II'
colnames(macrophage@meta.data)[22] <- 'IFNγ_response'
colnames(macrophage@meta.data)[23] <- 'Angiogenesis'
colnames(macrophage@meta.data)

library(ggpubr)
library(reshape2)
#celltype
data1<- FetchData(macrophage,vars = c("Treatment1","Treatment1",
```

```
"M1_score","M2_score","Pro_inflammatory_score","Anti_inflammatory_scor
e",

"APP_score","Costimulatory_score",
                                      "MHC_I","MHC_II",
                                      "IFNγ_response",
                                      "Angiogenesis"))
write.csv(data1,file="F:/scRNA/JCML/GSE178318/analysis2/20
0.8/celltype/Myeloid/SCORE.csv")
view(data1)

data1<-           read.csv(file="F:/scRNA/JCML/GSE178318/analysis2/20
0.8/celltype/Myeloid/SCORE.csv",header = TRUE)


ggviolin(data1, x = "Treatment1", y = "M1_score",
         fill = "Treatment1", add = "boxplot",
         ylab = "M1_score", xlab = "sample")+
  stat_compare_means(label ="p.signif" ,method="t.test",hide.ns = FALSE)
ggviolin(data1, x = "Treatment1", y = "M2_score",
         fill = "Treatment1", add = "boxplot",
         ylab = "M2_score", xlab = "sample")+
  stat_compare_means(label ="p.signif" ,method="t.test",hide.ns = FALSE)
#Pro_inflammatory_score
ggviolin(data1, x = "Treatment1", y = "Pro_inflammatory_score",
         fill = "Treatment1", add = "boxplot",
         ylab = "Pro_inflammatory_score", xlab = "sample")+
  stat_compare_means(label ="p.signif" ,method="t.test",hide.ns = FALSE)
ggviolin(data1, x = "Treatment1", y = "Anti_inflammatory_score",
         fill = "Treatment1", add = "boxplot",
         ylab = "Anti_inflammatory_score", xlab = "sample")+
  stat_compare_means(label ="p.signif" ,method="t.test",hide.ns = FALSE)


ggviolin(data1, x = "Treatment1", y = "Angiogenesis",
         fill = "Treatment1", add = "boxplot",
         ylab = "Angiogenesis", xlab = "sample")+
  stat_compare_means(label ="p.signif" ,method="t.test",hide.ns = FALSE)




##Fibroblast
```

```
library(ggplot2)
library(cowplot)
library(Seurat)
library(dplyr)
library(patchwork)
library(ggpubr)
rm(list=ls())

JCML.combined                                                    <-
readRDS(file="F:/scRNA/JCML/GSE178318/analysis2/JCML_combined_20_0.8_c
elltype_used.RDS")
Fibroblast <- subset(JCML.combined, idents = "Fibroblast")
Fibroblast

DefaultAssay(Fibroblast) <- "integrated"
Fibroblast <- RunPCA(Fibroblast,verbose = FALSE)
ElbowPlot(Fibroblast)

Fibroblast <- RunUMAP(Fibroblast, reduction = "pca", dims = 1:15)
Fibroblast <- RunTSNE(Fibroblast, reduction = "pca", dims = 1:15)
Fibroblast <- FindNeighbors(Fibroblast, reduction = "pca", dims = 1:15)

#saveRDS(Fibroblast,file    =    "F:/scRNA/JCML/GSE178318/analysis2/20
0.8/celltype/Fibroblast/Fibroblast_15.RDS")
Fibroblast  <-  readRDS(file  =  "F:/scRNA/JCML/GSE178318/analysis2/20
0.8/celltype/Fibroblast/Fibroblast_15.RDS")

Fibroblast <- FindClusters(Fibroblast, resolution = 0.3)


p1   <-   DimPlot(Fibroblast,   reduction   =   "umap",   group.by   =
"Treatment1")+theme(panel.background                                 =
element_blank(),panel.grid.major   =   element_blank(),panel.border   =
element_rect(colour="black",fill=NA))
p2 <- DimPlot(Fibroblast,  reduction  =  "umap",label  =  TRUE,  repel  =
TRUE,pt.size=1)+theme(panel.background                               =
element_blank(),panel.grid.major   =   element_blank(),panel.border   =
element_rect(colour="black",fill=NA))
p2 + p1
p3   <-   DimPlot(Fibroblast,   reduction   =   "tsne",   group.by   =
"Treatment1")+theme(panel.background                                 =
element_blank(),panel.grid.major   =   element_blank(),panel.border   =
element_rect(colour="black",fill=NA))
p4 <- DimPlot(Fibroblast,  reduction  =  "tsne",label  =  TRUE,  repel  =
```

```
TRUE,pt.size=1)+theme(panel.background =
element_blank(),panel.grid.major = element_blank(),panel.border =
element_rect(colour="black",fill=NA))
p4 + p3

DefaultAssay(Fibroblast) <- "RNA"

markers.to.plot <- c("EPCAM","KRT19","KRT8",
                     "CD3D","CD3G","TRAC",
                     "CD14","CD68","LYZ","CD163",
                     "FAP","COL1A1","COL3A1","DCN","ACTA2")

DotPlot(Fibroblast, features = markers.to.plot, dot.scale = 8) +
  theme(panel.background = element_blank(),panel.grid.major =
element_blank(),panel.border =
element_rect(colour="black",fill=NA))+coord_flip()+
  RotatedAxis()
VlnPlot(Fibroblast, features = markers.to.plot,pt.size = 0)

#15 0.3
Fibroblast <- readRDS(file = "F:/scRNA/JCML/GSE178318/analysis2/20
0.8/celltype/Fibroblast/Fibroblast_15_0.3.RDS")
table(Idents(Fibroblast))

CAF_ECM=c(1,3)
CAF_contractile= c(2)
CAF_secretory= c(0)
Doublets= c(4)

current.cluster.ids <-
c(CAF_ECM,Doublets,CAF_contractile,CAF_secretory)
new.cluster.ids <- c(rep("CAF_ECM",length(CAF_ECM)),
                     rep("Doublets",length(Doublets)),
                     rep("CAF_contractile",length(CAF_contractile)),
                     rep("CAF_secretory",length(CAF_secretory)))

Fibroblast@meta.data$Celltype <- plyr::mapvalues(x =
as.integer(as.character(Fibroblast@meta.data$seurat_clusters)), from =
current.cluster.ids, to = new.cluster.ids)

table(Fibroblast@meta.data$Celltype)

Fibroblast$Celltype <-
factor(Fibroblast$Celltype ,level=c("CAF_secretory","CAF_ECM","CAF_con
```

```
tractile","Doublets"))
Idents(Fibroblast)<-"Celltype"
table(Idents(Fibroblast))

Fibroblast <- subset(Fibroblast, idents = "Doublets",invert=TRUE)
table(Idents(Fibroblast))

#saveRDS(Fibroblast,file="F:/scRNA/JCML/GSE178318/analysis2/20
0.8/celltype/Fibroblast/Fibroblast_15_0.3_celltype.RDS")
Fibroblast <- readRDS(file="F:/scRNA/JCML/GSE178318/analysis2/20
0.8/celltype/Fibroblast/Fibroblast_15_0.3_celltype.RDS")

p1 <- DimPlot(Fibroblast, reduction = "umap", group.by =
"Treatment1")+theme(panel.background =
element_blank(),panel.grid.major = element_blank(),panel.border =
element_rect(colour="black",fill=NA))
p2 <- DimPlot(Fibroblast, reduction = "umap", repel =
TRUE,pt.size=1)+theme(panel.background =
element_blank(),panel.grid.major = element_blank(),panel.border =
element_rect(colour="black",fill=NA))
p2 + p1
p3 <- DimPlot(Fibroblast, reduction = "tsne", group.by =
"Treatment1")+theme(panel.background =
element_blank(),panel.grid.major = element_blank(),panel.border =
element_rect(colour="black",fill=NA))
p4 <- DimPlot(Fibroblast, reduction = "tsne", repel =
TRUE,pt.size=1)+theme(panel.background =
element_blank(),panel.grid.major = element_blank(),panel.border =
element_rect(colour="black",fill=NA))
p4 + p3

#We can explore these marker genes for each cluster and use them to
annotate our clusters as specific cell types.
DefaultAssay(Fibroblast) <- "RNA"

VlnPlot(Fibroblast, features = c("VEGFC"),pt.size = 0,group.by =
"Treatment1")+
  stat_compare_means(label ="p.signif" ,method="t.test",hide.ns = FALSE)
VlnPlot(Fibroblast, features = c("VEGFB"),pt.size = 0,group.by =
"Treatment1")+
  stat_compare_means(label ="p.signif" ,method="t.test",hide.ns = FALSE)

#cell component
#proportion
```

```
Fibroblast    <-    readRDS(file="F:/scRNA/JCML/GSE178318/analysis2/200.8/celltype/Fibroblast/Fibroblast_15_0.3_celltype.RDS")

table(Fibroblast$Treatment1)
table(Idents(Fibroblast))
prop.table(table(Idents(Fibroblast)))
table(Idents(Fibroblast), Fibroblast$Treatment1)
prop.table(table(Idents(Fibroblast), Fibroblast$Treatment1), margin = 2)

Fibroblast_p<-as.data.frame(prop.table(table(Idents(Fibroblast),
Fibroblast@meta.data[,"Treatment1"]), margin = 2))

ggplot(Fibroblast_p,aes(x=Fibroblast_p[,2],y=Fibroblast_p[,3],fill=Fibroblast_p[,1]))+
  geom_bar(position = 'stack',stat="identity")+
  labs(x="Sample",y="Cell proportion")+
  theme(panel.background=element_rect(fill='transparent',
color='black'),panel.border =element_rect(fill=NA,color='black'),
        legend.key=element_rect(fill='transparent',
color='transparent'),axis.text = element_text(color="black"))+
  scale_y_continuous(expand=c(0.001,0.001))+
  guides(fill = guide_legend(keywidth = 1, keyheight = 1,ncol=1,title =
'Cell types'))
```
